# Methylation profile of individuals with sickle cell trait

**DOI:** 10.1080/15592294.2025.2539234

**Published:** 2025-08-04

**Authors:** Ana Gabriela Vasconcelos, Mari Johnson, Yanwei Cai, Li Hsu, Nora Franceschini, Paul L. Auer, Charles Kooperberg, Laura M. Raffield, Alex P. Reiner

**Affiliations:** a Department of Biostatistics, University of Washington, Seattle, WA, USA; b Division of Public Health Sciences, Fred Hutchinson Cancer Center, Seattle, WA, USA; c Department of Epidemiology, Gillings School of Global Public Health University of North Carolina, Chapel Hill, NC, USA; d Joseph J. Zilber College of Public Health, University of Wisconsin-Milwaukee, Milwaukee, WI, USA; e Department of Genetics, University of North Carolina at Chapel Hill, Chapel Hill, NC, USA; f Department of Epidemiology, University of Washington, Seattle, WA, USA

**Keywords:** Sickle cell trait, DNA methylation, epigenetic clock, epigenetic age acceleration

## Abstract

Sickle cell trait (SCT) is due to heterozygosity for the β-globin sickle cell mutation. SCT recently has been associated with increased risk of various adverse health outcomes. DNA methylation (DNAm) is one potential mechanism by which SCT may impact disease risk. To identify DNAm sites associated with SCT, we conducted an epigenome-wide association (EWAS) meta-analysis using whole blood Illumina EPIC array data available in a total of 3,677 African American participants (including 1,071 with SCT) from the Women’s Health Initiative and Jackson Heart Study. We identified 103 differentially methylated CpGs and 119 differentially methylated regions associated with SCT. The strongest signals were hypermethylated cis loci within predicted regulatory elements within or near the β-globin gene cluster on chromosome 11. Beyond the globin locus, SCT-associated DMPs were enriched in genes involved in redox regulation and oxidative stress. We also demonstrate an association of SCT with differences in biological age and epigenetic age acceleration, though the pattern and strength of association differ according to the epigenetic clock used. Specifically, more recent epigenetic clocks that incorporate clinical phenotypes or laboratory biomarkers related to adverse health outcomes are associated with accelerated aging among individuals with SCT compared to African American controls. Our results lay the groundwork for future study of the role of DNAm in biologic aging and related health outcomes among individuals with SCT.

## Introduction

Sickle cell trait (SCT) is defined as the heterozygous carrier state for the β-globin missense mutation (Glu6Val) encoded by *HBB* rs334 on chromosome 11 that causes sickle cell anemia. SCT is common in populations with historical endemic malaria exposure, including many populations of African ancestry (e.g., 8% of African Americans), due to the protective effects of this carrier state on malaria. While SCT was traditionally considered a benign condition, recent studies show that SCT has been associated with several clinical sequelae, including increased risk of chronic kidney disease, anemia, pneumonia, diabetes, renal medullary carcinoma, and venous thromboembolic disease [1,2]. However, the biological mechanisms underlying these associations are still poorly understood.

DNA methylation is an epigenetic chemical modification in which a methyl group is added to a cytosine base of a DNA molecule at a cytosine-guanine (CpG) site. DNA methylation is involved in diverse biologic processes including regulating gene expression, maintaining genomic stability, cellular differentiation, and cell identity. During development, DNA methylation has been implicated in fetal globin gene silencing that occurs during the switch from fetal to adult erythropoiesis, with potential therapeutic implications for sickle cell anemia [3]. DNA methylation changes can also reflect the accumulation of cellular damage and adverse environmental exposures that accelerate the aging process, which, together with the availability of large-scale genome-wide DNA methylation profiling, has led to growing interest in the role of epigenetic biomarkers in disease susceptibility and progression. For example, global measures of epigenetic age acceleration were recently demonstrated in patients with Sickle Cell Disease (SCD) compared with African Americans without SCD, which may contribute to SCD morbidity and mortality [4,5].

To date, there is little information on how epigenetics relates to SCT, in part due to the limited availability of genome-wide methylation array data for subjects with African ancestry. Therefore, the goals for this project are to use genome-wide DNA methylation data to (1) gain insight on how SCT may affect DNA methylation by performing an epigenome-wide association study of SCT versus controls; (2) assess the relationship of various measures of epigenetic age acceleration to SCT.

## Material and methods

### Study cohorts

This study utilized data from two population-based cohorts: the Women’s Health Initiative (WHI) and the Jackson Heart Study (JHS).

The Women’s Health Initiative (WHI) is a large, prospective national health study that recruited 161,808 post-menopausal women aged 50–79 y between 1993 and 1998, including 14,618 African American (AA) women [6]. Of these, 11,559 consenting AA WHI participants have been genotyped for the sickle cell mutation (rs334) through either whole genome sequencing or genome-wide genotyping, with imputation to the Trans-Omics for Precision Medicine (TOPMed) reference panel [7]. The current DNA methylation study is comprised of 1,944 of these AA women, including 917 participants with SCT (heterozygous for the rs334 minor allele) and 1,027 age-matched non-carriers (homozygous for the major rs334 allele).

The Jackson Heart Study (JHS), initiated in 2000, is a community-based, longitudinal study focused on cardiovascular health among 5,306 African American adults in Jackson, Mississippi [8]. Methylation data was obtained from a subset of 1,729 baseline exam samples without regard to rs334 genotype. The 1,729 JHS samples include 156 participants with SCT and 1,573 non-carriers.

### Sickle cell mutation genotyping and quality control

WHI participants were genotyped for the sickle cell mutation (rs334) using either genome-wide genotyping with imputation or whole genome sequencing (WGS). In brief, WHI samples were genotyped with either the Illumina Multi-Ethnic Genotyping Array (MEGA), Illumina Omni array, or Affymetrix 6.0 array. Following QC, WHI samples were phased with SHAPEIT2 and imputed to the 1000 Genomes Project Phase 3 data release using IMPUTE2 (version 2.3.2) [9–11]. The average imputation quality info score for rs334 was 0.84. For samples genotyped using WGS, mean genome coverage ≥ 30x sequencing (TOPMed freeze 8) was performed at the Broad Institute and sequence data files were transferred to the TOPMed Informatics Research Center (IRC), where reads were aligned to human genome build GRCh38 using a common pipeline (GATK and Burrows-Wheeler Aligner). Variant-level QC consisted of a machine learning based support vector machine approach, using variants present on genotyping arrays as positive controls and variants with many Mendelian inconsistencies as negative controls. For both genotyping array data and WGS data, sample level QC consisted of detection and removal of sex discrepancies, Mendelian inconsistencies, sample swaps, or evidence of DNA mixture. The genotype for rs334 in JHS was obtained using TOPMed WGS, as previously described [12].

### Methylation data and quality control

The Illumina Infinium MethylationEPIC v1.0 BeadChip was used to collect data in both WHI and JHS. Raw IDAT files were processed using the *minfi* R package [13]. For both cohorts normalization with respect to background color intensity using the normal-exponential out-of-band (NOOB) preprocessing method was performed. Cell type composition (CD8T, CD4T, NK cells, B cells, Monocytes, and Neutrophils) were estimated based on reference using *FlowSorted.Blood.EPIC* [14].

In WHI, further quality control was done using R package *ChAMP* [15] to filter probes with detection p-value greater than 0.01, probes not on CpG sites, multi hits [16], on XY chromosome and those overlapping common African ancestry SNPs (1000 Genomes Project), as suggested by Zhou, Laird, and Shen [17]. Samples with more than 10% of missing values were excluded. Missing probes were imputed with KNN and BMIQ normalization was performed for type I and II.

For JHS, DNA methylation data available from previous studies [18,19] was adjusted for batch effects (sample batch, plate, and plate position) using *ComBat* as implemented in the *sva* [20] and *ChAMP* [21] R packages. Since detection p-values were not available no filtering on that was performed, but the same set of 718,639 CpGs as WHI were considered to filter out probes not on CpG sites, multi hits and those related to SNPs.

### Epigenome-wide association analysis (EWAS)

Differentially Methylated Positions (DMP) analysis was performed for each probe and tested the association between the *M* values (outcome) and SCT, while accounting for age, cell type composition and the first 10 ancestry principal components. For the WHI cohort, a linear mixed model was considered, including a random effect for batch, while for the JHS cohort sex was also accounted for using a linear model, since batch effect was already corrected for. A random effect meta analysis weighted by the inverse of the variance was performed to combine results from both cohorts with package metafor [22]. Following the recommendations from Mansell et al. [23] a genome wide significance value of $9\times 10^{-8}$ was considered. To investigate if the association between SCT and a methylation site is influenced by known SCT-related phenotypes, sensitivity analysis for each DMP was further conducted adjusting for hemoglobin concentration and estimated glomerular filtration rate (eGFR). Owing to the missingness of hemoglobin or eGFR measurements in a small number of WHI and JHS participants (n = 109 and 46, respectively), the initial model was run again, considering the same samples as the sensitivity model.

Differentially methylated regions (DMRs) were identified using the DMRcate R package [24]. The analysis was based on meta-analysis results of differentially methylated positions (DMPs), considering CpGs with FDR-adjusted p-values < 0.01. Neighboring CpGs (within 1000 base pairs; lambda = 1000) were grouped into candidate regions using a smoothing approach, with the default scaling factor (C = 2) applied to control the extent of regional aggregation. After smoothing, DMRs were deemed significantly associated with SCT if they met the following criteria: an FDR-adjusted p-value < 0.05, inclusion of at least four CpGs, and a maximum absolute effect size difference >0.1 across the region.

### Functional annotation and downstream analyses

#### Genomic annotation of SCT-associated DMPs and DMRs

CpG sites were annotated using the Illumina EPIC array reference annotation (*IlluminaHumanMethylationEPICanno.ilm10b4.hg19* [25]). Gene mappings for each CpG were obtained from two sources: UCSC RefGene and GENCODE Basic v12. Differentially methylated regions (DMRs) were annotated based on their genomic coordinates using the *GenomicRanges* package in R [26]. Each DMR was overlapped with known genes from UCSC and/or GENCODE.

Loci containing SCT-associated DMPs and DMRs were visualized with the UCSC Genome Browser GRCh37 [27] and functionally annotated utilizing a variety of regulatory tracks including GeneHancer enhancers/promoters and interactions between regulatory elements and genes; candidate cis-regulatory elements (cCRE) and transcription factor CHIP-seq peaks from ENCODE. Additional hematopoiesis cCRE based on epigenetic segmentation (IDEAS) and HbF regulation at the β-globin gene cluster on chromosome 11 and the *BCL11A* locus [28] was obtained using VISION (ValIdated Systematic IntegratiON of epigenomic data in mouse and human blood cells) hematopoiesis on the PSU Genome Browser [29].

#### Pathway enrichment analysis

We performed gene set enrichment analysis using the *missMethyl* package to evaluate the overall biologic relevance of differentially methylated CpGs and regions [30]. For CpG site-level analysis, we used the *gsameth* function on the set of DMPs with FDR < 0.05. Enrichment testing was performed against curated gene sets from KEGG, Reactome, and Gene Ontology Biological Processes (GO:BP, C5 collection). For region-level analysis, we applied the *gsaregion* function to the set of differentially methylated regions (DMRs). Pathways were deemed significantly enriched at FDR-adjusted p-value < 0.05.

#### Transcription factor motif enrichment analysis

To identify transcription factor (TF) binding motifs enriched around DMPs, we performed a position weight matrix (PWM)-based motif enrichment analysis using the PWMEnrich [31] package in R. PWMs were obtained from the MotifDb collection [32], restricted to high-confidence human motifs from the JASPAR 2022 [33] release and HOCOMOCO v11 core A/B/C databases [34]. For each DMP, the surrounding ± 100 bp DNA sequence was extracted from the hg19 reference genome. To control for sequence composition bias, a background set of sequences was constructed from random, non-significant CpG sites matched to the DMPs by GC content in a 3:1 ratio. Motif enrichment was assessed using a log-normal background model, and statistical significance was determined using FDR-adjusted p-values. Motifs with FDR < 0.05 were considered significantly enriched.

#### eQTM analysis

We performed an expression quantitative trait methylation (eQTM) analysis to investigate associations between DNA methylation and gene expression. Because gene expression data are not available at baseline in the Women’s Health Initiative (WHI), we used available DNA methylation and gene expression profiling from whole blood collected during the WHI Long Life Study (LLS) visit, which occurred in 2012–2013 (approximately 15 y after WHI recruitment). Whole blood DNA methylation and transcriptomic data at the LLS visit are available in a subset of 726 of the original 1,944 SCT case-control study participants.

DNA methylation data from the LLS visit were derived from the Illumina EPIC array and preprocessed following the same quality control and normalization procedures as the WHI baseline visit (described above), including probe filtering, background correction, and conversion to M-values.

For gene expression profiling, following collection into PreAnalytiX PAXgene tubes, WHI-LLS RNA samples underwent total RNA extraction, agarose gel electrophoresis to assess RNA integrity, and RNA quantification. Poly-A selection and cDNA synthesis were performed using the TruSeq Stranded mRNA kit, followed by library and insert size quantification. RNA sequencing was carried out on an Illumina NovaSeq6000 with target sequencing coverage of ≥75 million mapped reads. Demultiplexed, unaligned BAM files were converted to FASTQ format and sequence read and base quality were checked using the FASTX-toolkit (v0.0.13). Raw RNA sequencing reads were mapped and aligned to Hg38 per TOPMed consortium protocol [35]. After quality control, expression levels for 14,288 genes were retained for analysis.

We tested the 103 differentially methylated CpG sites (DMPs) for association with gene expression. For each CpG–gene pair, we fitted a linear mixed model, with log-transformed CPM gene expression as the outcome and DNA methylation M-values as the predictor. Models were adjusted for age, SCT status, measured blood cell counts (neutrophils, basophils, eosinophils, monocytes, and lymphocytes), first genetic PC, batch effects for both methylation and gene expression (included as random effects). We also included the top five methylation PCs and 10 gene expression PCs to account for global technical or biological variation.

Each CpG–gene pair was classified as either cis or trans based on the genomic distance between the CpG site and the gene’s transcription start site (TSS). Pairs were defined as cis if the CpG was located within ± 1 megabase (Mb) of the gene’s TSS. All other pairs, including interchromosomal and >1 Mb apart, were defined as trans.

To assess statistical significance, we applied an epigenome-wide p-value threshold of $1\times 10^{-7}$ for cis-eQTM pairs, consistent with previous recommendations for epigenome-wide studies [36]. For trans-eQTMs, we adopted a more stringent threshold of $2\times 10^{-12}$, following a conservative approach used in the Framingham Heart Study [19]. This threshold corresponds to a Bonferroni correction of the epigenome-wide threshold $\left(1\times 10^{-7}\right)$ for approximately 50,000 gene transcripts tested per CpG site. The estimate of 50,000 transcripts reflects a conservative upper bound, accounting for both protein-coding and non-coding genes, transcript isoforms, and potential redundancy in gene annotations.

#### Overlap with EWAS catalog

To assess whether the identified DMPs had been previously linked to other traits, we compared our results with the Epigenome-Wide Association Studies (EWAS) Catalog (https://www.ewascatalog.org) [37]. We tested for overlap between significant SCT-associated CpGs and trait-associated sites from studies in the catalog that included more than 100 participants and reported p-values less than $9\times 10^{-8}$. Enrichment was evaluated using Fisher’s exact tests, and multiple testing correction was applied using the Benjamini–Hochberg method, with statistical significance defined as FDR < 0.05.

#### Overlap with GWAS loci for sickle cell and other or related hematologic traits

To evaluate whether the SCT associated CpGs overlap with genome-wide association study (GWAS) loci associated with sickle cell disease (SCD) and related hematological traits, we downloaded the GWAS catalog v1.0.2 from the NHGRI-EBI database [38]. We filtered for traits related to SCD and hematological parameters using keywords such as ‘sickle cell,’ ‘anemia,’ ‘hemoglobin,’ ‘erythrocyte,’ ‘hematocrit,’ ‘reticulocyte,’ ‘iron,’ ‘corpuscular,’ and ‘red blood.’ We retained SNPs reaching genome-wide significance $\left(p<5\times 10^{-8}\right)$ and extracted their genomic coordinates along with the mapped genes provided in the catalog. For each significant DMP identified in our SCT analysis, we defined a 10 Kb window up and downstream centered on the CpG site. Using the *GenomicRanges* R package, we determined which GWAS SNPs fell within these regions. Genomic coordinates were harmonized to the GRCh37 reference genome.

### Epigenetic age acceleration analysis

Epigenetic age calculations were performed separately for WHI and JHS using the R package *ENmix* [39]. These calculations included estimates from the first-generation Horvath skin and blood tissue and Hannum blood-based clocks, second-generation PhenoAge and GrimAge clocks, as well as third-generation DunedinPACE aging rates, using a modified normalization procedure to minimize batch effects [40]. All epigenetic age calculations were based on using principal components derived from all individual-level CpGs, which has been shown to improve reliability compared to estimation from individual CpGs [41]. Epigenetic age estimates, along with residuals from regressing epigenetic age on chronological age (commonly referred to as epigenetic age acceleration), were obtained. Association between estimates and residuals with SCT status was assessed separately in WHI and JHS using linear regression models. Two sensitivity analyses were conducted, the first adjusting for age (except for residuals), cell type composition, hemoglobin, eGFR, sex (for JHS) and the first 10 genetic ancestry PCs, and the second with an additional adjustment for income and education. Finally, results from both cohorts were pooled using a random-effects meta-analysis.

## Results

### Participant characteristics

All WHI and JHS participants in the current analyses self-identified as Black or African American. All WHI participants were female by design, while for JHS, 63% of non SCT and 57% of SCT were female (Table 1). Both studies have comparable median BMI levels of approximately 30 kg/m^2^ and median age around 60 y old. The WHI cohort had differences in percentage of African ancestry, income and education between SCT cases and controls, with SCT cases having higher African ancestry, lower income and education levels. For JHS, there were no differences in socioeconomic status by SCT status. Smoking history differed slightly between the cohorts: a higher percentage of WHI participants were ever-smokers compared with JHS, though smoking status did not significantly vary by SCT status within either study. In both WHI and JHS, participants with SCT had a higher frequency of chronic kidney disease (CKD) and lower levels of hematocrit, hemoglobin, and estimated CD8+ T-cell proportions compared with non-SCT participants (Table 1). In JHS, SCT participants additionally exhibited lower estimated proportions of natural killer (NK) cells and a higher proportion of neutrophils.

**Table 1.** Participant characteristics by sickle cell trait (SCT) status, shown separately for WHI and JHS cohorts. Values are presented as mean (standard deviation) for continuous variables and count (percentage) for categorical variables. P-values correspond to comparisons between SCT and non-SCT individuals within each cohort.

Characteristic	WHI			JHS		
	Non SCT, *N* = 1,026	SCT, *N* = 915	*p*-value	Non SCT, *N* = 1,580	SCT, *N* = 156	*p*-value
Sex (Female)	1,026 (100%)	915 (100%)	–	1,000 (63%)	89 (57%)	0.124
BMI (kg/m2)	30.6 (6.4)	31.2 (7.7)	0.094	32.0 (7.4)	32.0 (7.8)	0.688
Age (Years)	61.1 (7.0)	61.5 (7.2)	0.209	55.6 (12.4)	56.7 (12.3)	0.186
Percent AFR (%)	74.2 (16.6)	79.8 (12.8)	< 0.001	82.6 (10.1)	83.2 (8.4)	0.938
Missing	45	232		2	0	
Income			< 0.001			0.917
Less than $10,000	70 (7.3%)	115 (14%)		396 (29%)	40 (30%)	
$10,000 to $34,999	341 (35%)	388 (46%)		301 (22%)	33 (24%)	
$35,000 to $49,999	164 (17%)	135 (16%)		215 (16%)	22 (16%)	
$50,000 to $74,999	221 (23%)	136 (16%)		244 (18%)	20 (15%)	
$75,000 to $99,999	91 (9.4%)	37 (4.4%)		102 (7.4%)	8 (5.9%)	
$100,000 or more	76 (7.9%)	36 (4.3%)		113 (8.2%)	12 (8.9%)	
Unknown	64	70		209	21	
Education			0.001			0.259
Less than high school	79 (7.8%)	103 (11%)		265 (17%)	34 (22%)	
High school graduate/GED	102 (10%)	135 (15%)		344 (22%)	30 (19%)	
Attended vocational school, trade school, or college	838 (82%)	666 (74%)		967 (61%)	91 (59%)	
Unknown	8	13		4	1	
Smoker ever	514 (51%)	449 (50%)	0.739	548 (35%)	65 (42%)	0.083
SBP (mmHg)	130.8 (17.3)	131.4 (18.3)	0.747	128.1 (16.5)	126.7 (14.8)	0.655
DBP (mmHg)	78.0 (9.1)	78.0 (9.4)	0.812	76.0 (8.9)	75.1 (8.6)	0.321
eGFR CKD-EPI (ml/min)	84.3 (15.7)	81.3 (18.0)	0.001	94.1 (22.0)	89.8 (24.9)	0.074
Prevalent CKD (Yes)	72 (7.0%)	102 (12%)	< 0.001	237 (15%)	39 (25%)	0.001
Hypertension (Yes)	656 (65%)	599 (67%)	0.242	934 (59%)	88 (56%)	0.513
Hematocrit (%)	39.2 (3.7)	38.5 (3.1)	< 0.001	39.3 (4.3)	38.7 (4.2)	0.049
Hemoglobin (g/dL)	13.0 (1.4)	12.7 (1.2)	< 0.001	13.1 (1.5)	12.9 (1.4)	0.048
Platelet count (103/mm3)	248.9 (58.2)	245.6 (60.8)	0.297	253.3 (66.2)	249.2 (62.0)	0.69
Cell type proportions						
CD8T (%)	11.5 (4.8)	10.7 (4.4)	< 0.001	8.6 (5.0)	7.1 (4.7)	< 0.001
CD4T (%)	21.1 (6.8)	21.3 (7.1)	0.902	18.0 (6.9)	16.9 (6.8)	0.093
NK (%)	6.3 (2.5)	6.4 (2.8)	0.225	3.5 (3.4)	2.4 (2.8)	< 0.001
B cells (%)	8.0 (3.6)	8.0 (3.2)	0.595	8.1 (3.8)	7.4 (3.0)	0.054
Monocytes (%)	8.9 (2.8)	9.0 (3.0)	0.484	7.2 (2.6)	7.1 (2.7)	0.504
Neutrophils (%)	44.5 (12.9)	45.2 (13.1)	0.100	58.9 (11.1)	63.4 (10.7)	< 0.001

### Differential DNA methylation associated with SCT

#### Genome-wide differentially methylated CpG sites (DMPs)

**Figure 1.**
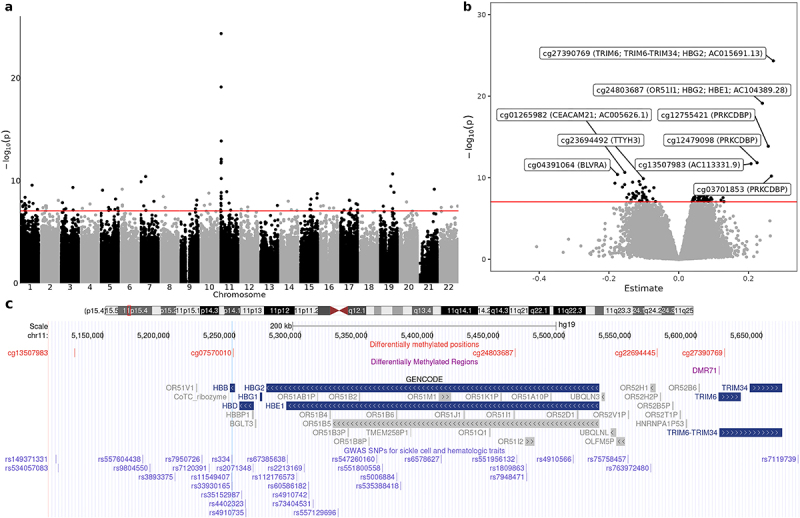
Meta analysis of differentially methylated positions (DMPs). (a) Manhattan plot. (b) Volcano plot highlighting top 10 significant CpGs (annotated with mapped genes). Horizontal line indicates the genome-wide significance threshold (*p* = 9 ×10^−8^). (c) Zoomed-in view of the genomic region surrounding the sickle cell mutation (rs334) on chromosome 11, highlighting 5 genome-wide significant DMPs and 1 significant differentially methylated regions (DMRs).

We identified a total of 103 CpG sites significantly associated with sickle cell trait (SCT) through an epigenome-wide meta analysis across the WHI and JHS cohorts $\left(p<9\times 10^{-8}\right)$ (Figure 1(a,b) and Supplemental Table S1). In cohort specific analyses, 55 DMPs reached genome-wide significance in WHI and 12 in JHS (Supplemental Figure S1a-d). Of the 103 meta-analysis DMPs, 63 were hypermethylated and 40 were hypomethylated in SCT compared to non-carriers. All sites showed consistent direction across cohorts. The most significant CpGs within the top 20 of these 83 distinct 1 Mb regions are shown in Table 2.

**Table 2.** Top 20 differentially methylated positions (DMPs) associated with SCT, selected as the most significant CpG within non-overlapping ±1Mb genomic regions. Results are based on meta-analysis of WHI and JHS cohorts. For each DMP, the table includes the CpG ID, chromosome, position (GRCh37), effect estimate, standard error, and p-value, along with the direction of association in each cohort to assess consistency. Genes located within the ± 1Mb region are also listed.

cpg	chr	pos	WHI/JHS	Estimate	Std Err	p-value	Genes in loci region
cg27390769	11	5621691	+/+	0.271	0.026	4.70E–25	*OR51S1;MMP26;AC113331.9;HBB;OR51I1;HBG2;HBE1;AC104389.28;TRIM6;HBG2;AC015691.13;TRIM6-TRIM34;CAVIN3*
cg01265982	19	42070875	-/-	−0.155	0.023	2.20E–11	*AC005626.1;CEACAM21*
cg04391064	7	43797839	-/-	−0.175	0.027	4.00E–11	*BLVRA*
cg23694492	7	2672929	-/-	−0.101	0.016	1.30E–10	*TTYH3*
cg20294789	1	151513456	-/-	−0.114	0.018	2.80E–10	*TUFT1;RP11-74C1.4*
cg17810176	19	36035831	-/-	−0.132	0.021	3.50E–10	*AD000090.2;TMEM147;GAPDHS*
cg14654533	3	131080755	-/-	−0.183	0.029	4.70E–10	*RP11-933H2.4;NUDT16P*
cg24196354	6	13445503	-/-	−0.111	0.018	6.90E–10	*GFOD1*
cg23057648	21	40984763	-/-	−0.155	0.025	6.90E–10	*C21orf88;B3GALT5;TMPRSS3*
cg01872122	5	16940481	+/+	0.116	0.019	8.70E–10	
cg17614165	11	47468663	+/+	0.074	0.012	1.20E–09	*RAPSN*
cg17929770	19	46318514	-/-	−0.161	0.027	1.40E–09	*RSPH6A*
cg15279476	10	62704447	-/-	−0.071	0.012	1.70E–09	*RHOBTB1*
cg07892295	15	98503714	-/-	−0.102	0.017	1.90E–09	*ARRDC4*
cg05290748	8	21668909	+/+	0.052	0.009	3.60E–09	*GFRA2*
cg06358171	1	54822008	-/-	−0.084	0.014	4.30E–09	*SSBP3*
cg26677315	15	97038051	-/-	−0.121	0.021	5.00E–09	
cg15084949	15	70128869	+/+	0.049	0.008	6.30E–09	*C15orf50;LINC00593*
cg15407091	5	148211186	+/+	0.053	0.009	6.30E–09	
cg06321925	9	127420734	-/-	−0.132	0.023	8.10E–09	*MIR181A2HG;NR6A1*

The 103 identified DMPs were distributed across 83 distinct genomic regions (defined as ±1Mb on either side of the most significant CpG). Genomic context annotation revealed that 38 CpGs (36.9%) were located within gene bodies, 28 (27.2%) in promoter regions, 24 (23.3%) in intergenic regions, and 13 (12.6%) mapped to both promoters and gene bodies. Relative to CpG island architecture, 18 CpGs (17.5%) were in CpG islands, 22 (21.4%) in shores, and the remaining 63 (61.2%) in open sea regions.

We performed sensitivity analyses adjusting for hemoglobin concentration and estimated glomerular filtration rate (eGFR). While these adjustments did not substantially change the effect estimates for most DMPs in the overlapping sample (Supplemental Figure S2 and Supplemental Table S2), the reduction in sample size due to missing covariate data led to a loss of power, and some associations were attenuated.

#### Differentially methylated regions (DMRs)

A total of 119 differentially methylated regions (DMRs) were identified using *DMRcate*. Significant DMRs contained an average of 10 CpGs (range 4 to 33) per region and length varying from 59 to 2,228 base pairs (Supplemental Table S3). Of the 119 regions, 37 were located within 1 Mb of at least one DMP, and 23 directly overlapped DMPs, suggesting coordinated regional methylation. The DMRs were most commonly located on Chromosome 6 (12.6%), followed by Chromosomes 1 and 3 with 8.4% each.

#### SCT-associated methylation at the β-globin locus

The most significant DMPs are located on Chromosome 11 within a ~1 Mb region surrounding the sickle cell mutation (*rs334*) in the β-globin gene cluster (Figure 1(c)). This cluster, which is developmentally regulated, includes the embryonic (*HBE*), fetal (*HBG1, HBG2*) and adult (*HBB, HBD*) genes, along with the locus control region (LCR), a key distal regulatory element for the cluster. The most strongly SCT-associated signal is the hypermethylated probe cg27390769 (meta-analysis $p=4.7\times 10^{-25}$) located ~300 kb telomeric to the β-globin cluster in a region containing several Tripartite motif (*TRIM*) family genes (*TRIM5*, *TRIM6*, *TRIM34*) which encode E3 ubiquitin ligases involved in innate immunity and antiviral responses [42]. The SCT-associated cg27390769 DMP is located within a predicted hematopoietic cell regulatory element (Supplemental Table S4) [43,44] and is also proximal to a candidate fetal hemoglobin (*HbF*) cis-regulatory element in adult erythroid cells [28]. Adjacent to cg27390769 is a DMR that includes a cluster of six CpG sites (DMR 71, $\mathrm{FDR}=1.3\times 10^{-9}$), located near the *TRIM6* promoter that demonstrates physical interaction with *HBG2*. The second most significant CpG site, *cg24803687* (meta-analysis $p=7.5\times 10^{-20}$), is located ~200 kb upstream of the fetal (*HBG1* and *HBG2*) and embryonic (*HBE1*) globin genes, while another hypermethylated site associated with SCT (cg07570010, $p=5.9\times 10^{-9}$) is located proximal to the *HBB* promoter region (Figure 1(c)).

#### Trans-acting SCT-associated regulatory regions involved in β-globin expression and heme metabolism

In addition to the *cis* signals at the β-globin locus on chromosome 11, several *trans* signals were found within or adjacent to genes involved in regulating chromatin structure at the β-globin locus and γ-globin gene silencing during fetal-to-adult hemoglobin switching (Supplemental Table S4). A site on chromosome 2 hypomethylated in SCT encompasses a predicted hematopoietic regulatory element in an intron of *BCL11A* (cg23718924, $p=2.9\times 10^{-8}$), which plays a key role in regulation of the γ-globin to β-globin switch in concert with other transcription factors and epigenetic regulators [45]. Two sites hypomethylated in SCT on chromosome 1p32.3 are located within a predicted regulatory element present in hematopoietic cells intragenic to *SSBP3*, which encodes part of an erythroid DNA-binding complex that includes the transcription factors TAL1 and GATA1 [46].

Additional SCT-associated CpG sites were mapped to genes involved in epigenetic transcriptional regulation of the β-globin locus. A DMR located on chromosome 2 overlaps *DNMT3A* (DMR 12, $\mathrm{FDR}=7.2\times 10^{-20}$), which catalyzes DNA methylation at CpG dinucleotides. *DNMT3A* interacts with transcription factors resulting in hypermethylation and silencing of the γ-globin gene promoter during adult erythropoiesis [47]. *JARID2* (cg00969154, $p=2.4\times 10^{-8}$, and cg22570970, $p=3.0\times 10^{-8}$) and *BAHCC1* (cg06459984, $p=2.2\times 10^{-8}$) encode transcriptional repressors that regulate epigenetic gene silencing and histone modification through interaction with the Polycomb Repressive Complex 2 (PRC2) [48,49], which is critical for the repression of fetal hemoglobin expression [50].

**Figure 2.**
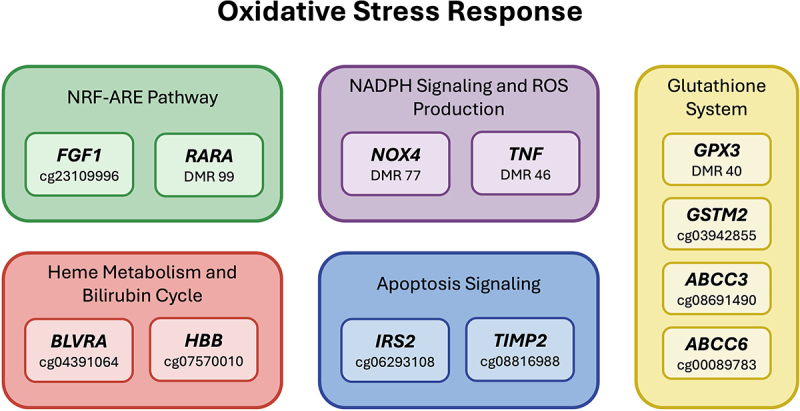
Oxidative stress-related pathways associated with differential DNA methylation in individuals with sickle cell trait (SCT). Genes implicated through epigenome-wide association study (EWAS) are grouped by biological function: NRF2-ARE signaling, glutathione metabolism, heme metabolism and bilirubin cycle, NADPH/ROS production, and apoptosis. Each gene is labeled with the associated DMP or DMR.

In addition to the regulation of globin expression and hemoglobin synthesis, hypomethylated CpG cg04391064 on chromosome 7 is located within the promoter of *BLVRA* (biliverdin reductase), which catalyzes the enzymatic reduction of biliverdin to bilirubin, the final product of heme metabolism [51]. In the setting of hemolysis due to red cell sickling, the heme degradation pathway is essential for protection from oxidative stress. Other genes located within or adjacent to SCT-associated differentially methylated CpGs or regions are involved in oxidative stress and antioxidant or ROS detoxification pathways (Figure 2), including FGF1, which enhances antioxidant defenses by activating the NRF2-ARE pathway [52], and RARA, which suppresses NRF2 signaling and contributes to increased oxidative stress [53]; GPX3 and GSTM2, key enzymes in glutathione-mediated ROS detoxification [54,55], and ABCC3, and ABCC6, members of the ABC transporter family involved in glutathione conjugate export and oxidative stress homeostasis [56,57]; BLVRA and HBB, which participate in heme metabolism and the bilirubin antioxidant cycle [51,58]; NOX4 and TNF, associated with NADPH signaling and ROS production [59,60]; and IRS2 and TIMP2, which are implicated in apoptosis signaling and redox-sensitive stress responses.

### Pathway analyses of SCT-associated CpG sites

#### Pathway enrichment analysis

To gain further insights into the biologic relevance of the SCT-associated genome-wide methylation patterns, we performed pathway enrichment analysis using the missMethyl package to account for probe number bias in Illumina array data. Analyses were performed separately for the 103 significant DMPs (annotated to 102 human genes, Supplemental Table S1) and the 119 DMRs (annotated to 144 genes, Supplemental Table S3).

**Figure 3.**
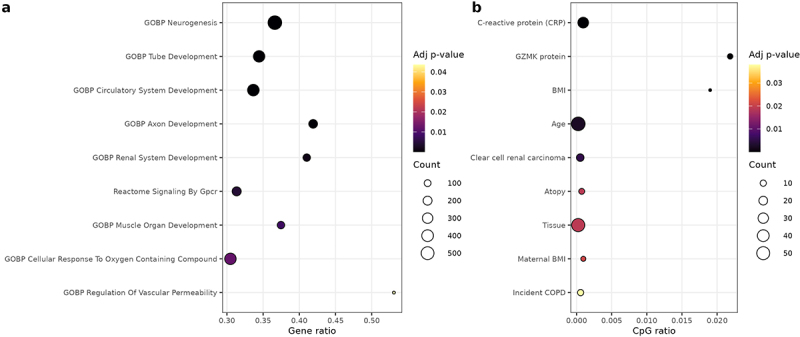
Enrichment analysis results. (a) Selected enriched biological pathways based on genes mapped to significant DMPs. (b) Enrichment of EWAS catalog phenotypes among SCT-associated CpG sites traits.

In the DMP gene enrichment analysis, 122 pathways were significantly enriched at an FDR < 0.05 (Figure 3(a), Supplemental Table S5). The top categories were related to organ and tissue development and morphogenesis, particularly of the nervous, circulatory, renal, and muscular systems. Analysis of the DMR-associated genes yielded three significantly enriched pathways: embryo development, organ growth, and tube development (Supplemental Table S5). While these pathways may not directly reflect SCT biology, they likely represent shared developmental regulatory networks affected by epigenetic changes. Other significantly enriched DMP pathways may be more directly related to pathophysiology of red cell sickling and hemolysis, including those involving responses to oxygen-containing compounds, regulation of vascular permeability, and adenylate cyclase and receptor protein tyrosine kinase signaling (Figure 3(a)) [61–63]. Although not specific to erythroid cells, these latter categories may reflect general stress-responsive or signaling mechanisms potentially relevant in the context of red cell sickling, hemolysis, and vaso-occlusion.

#### Transcription factor motif enrichment

To further explore upstream regulatory influences on SCT-associated methylation, we performed transcription factor (TF) motif enrichment analysis using the 103 significant DMPs with PNWenrich (Supplemental Table S6). Motifs significantly overrepresented include transcription factors with established roles in globin gene regulation and/or erythroid lineage specification and differentiation, such as CTCF, NFE2, MAFF, CEBPA, and IKZF1 [64,65]. Notably, CTCF (adjusted $\mathrm{FDR}=5.8\times 10^{-14}$), a key architectural protein, mediates chromatin looping and maintains histone modifications at the β-globin locus, thereby facilitating long-range enhancer–promoter interactions [66]. NFE2 ($\mathrm{FDR}=9.1\times 10^{-6}$), an erythroid-specific activator, binds both the LCR and promoter of the β-globin gene and is essential for transcriptional activation during erythropoiesis [65,67]. Additionally, we identified significant overrepresentation of motifs linked to redox regulation and antioxidative stress response, including BACH2, MAFF, ATF2, JDP2, MLX and NFKB1 [68–72], suggesting potential links between oxidative stress pathways and SCT-associated epigenetic regulation.

### Relationship of SCT-associated CpG sites to gene expression and other complex traits

#### Relationship of SCT-associated CpGs to gene expression (eQTM analysis)

We next performed eQTM analysis using DNA methylation array and RNA sequencing data from 762 participants (including 141 with SCT) using blood obtained during the WHI LLS exam (see Methods). We tested associations between the 103 SCT-associated DMPs and whole blood expression levels of 14,288 genes. We identified 12 significant *cis* eQTM pairs (defined as CpG site and gene transcription start site within 1Mb), from 11 unique DMPs, and 9 unique genes (Supplemental Table S7). Four of these *cis* associations involved *PRKCDBP*/*CAVIN3*, and one each involved *GSTM2, PLEKHG5, COA1, CSNK1G1, ADRB2, CCR4, UBQLNL,* and *TRIM34*. Several of these genes are involved in biological processes relevant to SCT. *GSTM2* (cg03942855,$p=3.7\times 10^{-17}$) encodes a glutathione S-transferase important for cellular detoxification and redox balance [54]. *ADRB2* (cg15407091, $p=7.6\times 10^{-11}$) encodes the β2-adrenergic receptor, which regulates vascular tone and is implicated in endothelial responses to oxidative stress [73]. *TRIM34* (cg27390769, $p=4.5\times 10^{-8}$) encodes an E3 ubiquitin ligase of the tripartite motif family with known roles in innate immune signaling and antiviral defense [44].

In total, 65 significant *trans* eQTM associations were detected, involving 40 unique genes and 13 unique DMPs (Supplemental Table S7). Among trans-associated genes, we identified multiple immune -related targets, including *GZMH* (cg06293108 and cg06358171), *CD8A* (cg23057648), both involved in cytotoxic T cell activity [74,75], as well as *CCL5* known to mediate chemotaxis (cg06358171) [76]. We also identified *MSC* (cg09082287), a transcriptional regulator involved in lymphocyte development [77]; notably, *MSC* binding motifs were significantly enriched near SCT-associated DMPs ($p=2.0\times 10^{-4}$; see Supplemental Table S6). Several additional trans-eQTM targets with potential roles in immune signaling were also identified (see Supplemental Table S7), though their relevance to SCT requires further investigation.

#### Relationship of SCT-associated CpGs with other complex traits

To gain further biologic insights into the 103 SCT-associated CpG sites, we examined their relationships with other complex phenotypes and exposures in previously published data sets using the EWAS catalog (Supplemental Table S8). Cross-referencing with the EWAS catalog revealed that the cis-associated CpG cg03942855 is associated with expression of *GSTM2* (and with neighboring *GSTM1*) [78]. More broadly, 33 of the 103 SCT-associated CpGs have been previously associated with C-reactive protein (CRP) levels [79], spanning multiple chromosomes, with a concentration on chromosome 1 (Supplemental Figure 3a). Another 8 CpGs – located on chromosomes 1, 6, 9, 15, and 19 – were associated with Granzyme K (GZMK) protein levels [80], all of which showed decreased methylation levels in SCT individuals (Supplemental Figure 3d). Trait enrichment analysis confirmed that SCT-associated CpGs were significantly enriched for C-reactive protein (CRP) levels ($\mathrm{FDR}=2.6\times 10^{-15}$) and GZMK protein levels ($\mathrm{FDR}=8.9\times 10^{-13}$), along with BMI ($\mathrm{FDR}=3.3\times 10^{-7}$), age (FDR=0.002), clear cell renal carcinoma (FDR=0.005) [81], atopy (FDR=0.019), tissue (FDR=0.019), maternal BMI (FDR=0.021) and incident COPD (FDR=0.038) (Figure 3(b)).

#### Overlap of SCT-associated CpGs with GWAS loci

We next explored whether the SCT differentially methylated loci overlap with known GWAS loci for sickle cell disease phenotypes and other erythrocyte-related traits, by identifying SNPs located within 10 kb of significant DMPs (Supplemental Table S9). Multiple GWAS signals for hemoglobin/hematocrit, red cell count, and red cell indices overlap with DMPs located in the β-globin gene cluster. These included cg07570010, located within 5kb of rs334 (the sickle cell causal variant) in the *HBB* promoter, as well as seven other red cell trait-associated SNPs (Figure 1(c)). Additionally, in the β-globin region cg24803687 and cg22694445 overlap with three SNPs (rs1809863, rs551956132, and rs7948471) and two SNPs (rs763972480 and rs11038031), respectively – all previously associated with hemoglobin or red cell indices.

Beyond the β-globin cluster, other erythrocyte trait-associated GWAS signals are located within 10 kb of DMPs. These include cg23718924, located in the *BCL11A* promoter and overlapping with rs1397318 and rs2665668, previously associated with mean corpuscular hemoglobin and red cell count, respectively. Additional overlaps were observed at cg06459984, near rs34386017, located within the histone methylation enzyme *BAHCC1*; and at cg04391064, near rs1181602 in the promoter of *BLVRA*, a gene central to antioxidant defense through its role in the heme degradation pathway and redox signaling [82]. The cg00089783 probe located intronic to *ABCC6*, is located near rs41278174, a missense variant (p.Arg1064Trp) associated with hemoglobin and other erythrocyte quantitative traits, as well as with calcium levels and kidney stones. ABCC6 belongs to the multidrug resistance-associated family of ATP-binding cassette transporters and is best known for its role in pseudoxanthoma elasticum (PXE), an autosomal recessive disorder characterized by ectopic calcification [83].

### Epigenetic age acceleration in SCT

We next examined the relationship between SCT status with five previously published clocks estimated from EPIC array data. When pooling results from WHI and JHS, the first-generation multi-tissue Horvath and Hannum biologic age and epigenetic age acceleration metrics showed no significant differences in epigenetic age acceleration between SCT cases and controls (Table 3, see Supplemental Table S10 for WHI and JHS results). There was one exception to this pattern in which SCT individuals had lower age acceleration for the Horvath residuals in the unadjusted analysis (p=0.001), but the difference was not significant after adjustment for cell type composition, hemoglobin, eGFR, sex for JHS and genetic PCs (p=0.156).

**Table 3.** Average epigenetic clock values and residuals by SCT status, and estimates of differences in mean between SCT vs non-SCT individuals. Results are shown for unadjusted analysis and adjusted for age (except for residuals), cell type composition, hemoglobin, eGFR, sex (for JHS) and the first 10 genetic ancestry PCs.

	Sample average (SD)		Unadjusted		Adjusted	
	SCT	No SCT	Estimate (CI)	p-value	Estimate (CI)	p-value
**PC Horvath**	54.61 (4.89)	54.87 (4.89)	−0.40 (−1.01, 0.20)	0.192	−0.27 (−0.64, 0.10)	0.158
**PC Hannum**	59.67 (5.19)	59.46 (5.19)	0.05 (−0.56, 0.67)	0.871	−0.17 (−0.52, 0.18)	0.339
**PC PhenoAge**	58.83 (6.22)	57.47 (6.22)	1.17 (0.53, 1.82)	** < 0.001**	0.52 (0.16, 0.89)	**0.005**
**PC GrimAge**	71.36 (5.04)	70.54 (5.04)	0.68 (0.17, 1.19)	**0.010**	0.29 (0.02, 0.56)	**0.033**
**DunedinPACE**	1.03 (0.08)	1.01 (0.08)	0.02 (0.01, 0.03)	** < 0.001**	0.02 (0.01, 0.02)	**0.001**
**PC Horvath residuals**	−0.37 (3.35)	0.19 (3.35)	−0.69 (−1.06, −0.32)	** < 0.001**	−0.27 (−0.64, 0.10)	0.156
**PC Hannum residuals**	−0.06 (3.23)	0.08 (3.23)	−0.26 (−0.64, 0.12)	0.184	−0.17 (−0.52, 0.18)	0.336
**PC PhenoAge residuals**	0.70 (4.04)	−0.22 (4.04)	0.81 (0.37, 1.26)	** < 0.001**	0.52 (0.15, 0.89)	**0.006**
**PC GrimAge residuals**	0.32 (2.17)	−0.10 (2.17)	0.36 (0.06, 0.66)	**0.019**	0.29 (0.03, 0.55)	**0.030**

In contrast, using the newer second and third generation epigenetic clock metrics, which incorporate additional biological parameters and clinical biomarkers related to disease, SCT individuals had higher epigenetic ages and faster age acceleration for PhenoAge, GrimAge, and DunedinPACE in both unadjusted and adjusted analyses. For PhenoAge, the unadjusted and adjusted p-values were <0.001 and 0.005, respectively, while for GrimAge, the corresponding p-values were 0.010 and 0.033. The DunedinPACE rate of aging was significantly higher (0.24 months more per chronological year) in SCT individuals than African Americans without SCT, with p-values of <0.001 in unadjusted analysis and 0.001 for the adjusted analysis. Residuals from these metrics also reflected significant associations. PhenoAge residuals showed higher age acceleration for SCT individuals, with p-values of <0.001 (unadjusted) and 0.006 (adjusted). GrimAge residuals similarly indicated faster acceleration, with p-values of 0.019 (unadjusted) and 0.030 (adjusted). With further adjustment for education and income, the association was attenuated, but the overall conclusion remained unchanged: second- and third-generation clocks still showed nominal significance, and SCT was associated with higher epigenetic age (Supplemental Table S10).

## Discussion

We report the first epigenomic study of SCT compared with non-SCT in African American individuals. Through our epigenome-wide association study (EWAS), we identified a total of 103 CpGs and 119 DMRs associated with SCT. The relationship of these differentially methylated loci to sickle cell-related phenotypes and biology is discussed further below. We also showed that SCT is associated with differences in biological age and epigenetic age acceleration, although the pattern and strength of the association differ according to the epigenetic clock used.

### DNA methylation and SCT-related phenotypes

#### Methylation and regulation of hemoglobin gene expression

Epigenetic modifications including DNA methylation, histone modifications, and chromatin remodeling play important roles in controlling adult erythropoiesis, terminal erythroid maturation, and regulation of globin gene expression [84,85]. Changes in methylation levels at the chromosome 11 β-globin gene cluster contribute to globin gene regulation and fetal to adult hemoglobin switching by influencing access of trans-acting DNA binding proteins such as GATA1, TAL1, SSBP3, LMO2, LDB1, BCL11A, KLF1, and ZBTB7 [45]. In particular, the LDB1-binding protein SSBP3 facilitates chromatin looping and physical interactions between distant regulatory enhancer elements (such as the β-globin LCR) and globin gene promoters [46]. The hypermethylation of CpGs at the β-globin gene cluster we observed among individuals with SCT is consistent with the pattern of hypermethylation at the γ-globin gene promoter reported in erythroid cells from children and adults with SCD [86].

HbF levels are variably increased in nearly all HbS homozygotes and to a lesser extent in SCT [87]. In SCD patients, DNA methylation levels are inversely correlated with levels of fetal hemoglobin (HbF), which are a primary modulator of red cell sickling, hemolysis, and clinical severity [86]. Thus, the differential methylation patterns observed in SCT may contribute to low-grade sickling and clinically milder red cell phenotypic alterations [88]. Additional Mendelian randomization studies involving genetically predicted methylation levels may clarify the causal relationship of differential methylation in blood to HbF levels and other clinical or laboratory features of SCT and SCD.

#### Heme metabolism, hemolysis, and oxidative stress pathways

Notably, other SCT-associated differentially methylated loci observed in the current study involve genes related to red cell structure or heme metabolism. Some of these CpG sites are located near GWAS loci for erythrocyte-related quantitative traits (Supplemental Table S9). The hypermethylated CpG site cg05261851 on chromosome 16 is located within the promoter region of *SLC12A4*, which encodes the KCC1 red cell KCl co-transporter – critical for cation and volume regulation during reticulocyte maturation and dehydration of sickle reticulocytes [89,90]. On chromosome 7, the hypomethylated CpG cg04391064 is located within the promoter of *BLVRA* (biliverdin reductase), which together with heme oxygenase-1, are critical enzymes involved in degradation of heme and protection from oxidative stress during hemolysis [51,82]. Consistent with the presence of low-grade chronic hemolysis in SCT, higher plasma levels of heme oxygenase-1 have been reported in SCT individuals [91].

The primary mechanism for handling cellular oxidative stress is activation of antioxidant genes by the transcription factor NRF2 through binding to antioxidant response elements (ARE). In the current study, we observed overrepresentation of TF motifs linked to antioxidative stress response across the SCT-associated CpG sites. In addition, several genes located within or adjacent to SCT-associated differentially methylated regions are related to oxidative stress pathways including regulation of NRF2 transcription, the glutathione system, NADPH oxidase signaling, and ROS-mediated apoptosis (see Figure 2).

The hypermethylated CpG on chromosome 16 (cg00089783) is located within the anion transporter *ABCC6*, which is mainly expressed in the liver and whose tissue-specific transcription is strongly regulated by DNA methylation [92,93]. Mutations in *ABCC6* cause pseudoxanthoma elasticum (PXE), a hereditary connective tissue disorder disease characterized by ectopic tissue calcification [83]. A common missense variant of *ABCC6* (p.Arg1064Trp) has previously been associated with hemoglobin and other erythrocyte quantitative traits, as well as with calcium levels and kidney stones. Interestingly, a PXE-like phenotype develops in a subset of patients with SCD and other beta-hemoglobinopathies and may exacerbate vascular endothelial damage and severity of vascular complications in SCD [94]. Reciprocally, hemoglobin A2 levels are increased in patients with PXE [95]. The co-occurrence of PXE-like phenotype with beta-hemoglobinopathies has been attributed to epigenetic downregulation of hepatic *ABCC6* expression by the erythroid transcription factor NF-E2 [95,96].

#### Other hematologic and immune alterations in SCT

Other SCT-associated DMRs are located near genes that encode transcription factors involved in hematopoiesis and lineage specification including ETV7, MEIS1, HOXB3, and MEF2C. These alterations in DNA methylation patterns may be related to SCT-related alterations of other blood cell counts, such as lower lymphocyte count, higher neutrophil count [97], and higher proinflammatory chemokines [91]. In addition, the differentially methylated *TRIM6* locus associated with SCT is involved in type 1 interferon signaling and the immune response to viral infection [43,44] and may have implications for susceptibility of individuals with SCT to infectious pathogens [98,99].

#### Sickle nephropathy

The hypoxic microenvironment of the renal medulla and resultant red cell sickling, together with oxidative stress due to hemolysis and release of hemoglobin and free heme, likely contribute to the predisposition of SCD and SCT individuals to development of chronic or end-stage kidney disease. As noted above, several genes located within or adjacent to SCT-associated differentially methylated regions are related to antioxidant pathways, ROS detoxification, or response to hypoxia or oxidative stress, including *TRIM6*, which participates in progression of renal fibrosis [100,101]. Additionally, three of the hypermethylated SCT-associated CpGs in the current study were previously reported to be associated with kidney disease, including cg18385689 (intragenic, nearest gene *LEFTY2*), cg19206131 (intragenic between RAB10 and KIF3C), and cg03701853 on chromosome 11 within the *PRKCDBP*/*CAVIN3* promoter. Several others (cg03562766, cg23694492, cg22694445, cg15407257) overlap GWAS loci for eGFR or cystatin C levels. Given that current analyses are limited to blood-based methylation profiles, future studies should explore SCT-associated methylation signatures in kidney tissue.

Renal medullary carcinoma (RMC) is a rare aggressive kidney cancer characterized by the loss of SMARCB1 tumor suppressor and predominantly affects young male adults with SCT and other hemoglobinopathies [102]. It has been hypothesized that hypoxia-induced regional ischemia due to erythrocyte sickling in the renal medulla is involved in pathogenesis [103], which may involve oncogenic and ferroptosis resistance pathways [104]. Although we did not detect any differentially methylated loci at the *SMARCB1* locus on chromosome 22, we did observe an enrichment of SCT-associated DMPs at 15 CpG sites previously associated with clear cell renal carcinoma (a more common type of kidney cancer) [81]. Among these 15 CpG sites, 6 are located on chromosome 11, including 4 (cg12755421, cg12479098, cg16245261, cg16776065) within the DMR upstream of the *PRKCDBP/CAVIN3* tumor suppressor locus.

### Sickle cell-associated epigenetic age acceleration

The widespread availability of DNA methylation profiling has led to the association between biological age and a variety of chronic diseases, as estimated from epigenetic clocks. Epigenetic age acceleration was recently demonstrated in SCD patients compared to African Americans without SCD [5] and may contribute to sickle cell-related morbidity and mortality [105]. In the current study, we provide further evidence for epigenetic age acceleration in individuals with SCT compared with controls matched for self-reported race/ethnicity. The association of epigenetic age acceleration with SCT persisted even after adjustment for ancestry and major SCT comorbidities (kidney function, white blood cell composition, hemoglobin, and chronological age), suggesting that the presence of the sickle cell mutation may have a more general impact on processes related to cellular aging. Notably, in both SCD and SCT, an association with epigenetic age acceleration was observed with later-generation epigenetic age clocks that incorporate a broader panel of age-related health parameters and molecular surrogates of disease risk [106–108], but not with earlier versions such as Horvath and Hannum. The consistent associations across these later generation clocks suggest that SCT may be linked to more subtle, systemic aging processes not captured by first-generation clocks trained solely on chronological age. In fact, we found some evidence that SCT may be associated with a younger biological age using first-generation epigenetic clocks. Since HbF levels vary among individuals with SCT and SCD, one possible explanation is that epigenetic programs for fetal and embryonic development are more activated among adults with SCT. Indeed, DNA sequences bound by the Polycomb Repressive Complex, which is involved in fetal to adult globin gene switching, are overrepresented in the Horvath clock [50,109]. Additional methylation studies involving a larger number of individuals with SCT and SCD are required to confirm these observations.

### Study limitations

We report the first epigenomic study of SCT compared with non-SCT in African American individuals from the same cohort, but our study has several limitations. A key limitation of this study is the lack of other DNA methylation array-based epigenetic analyses of blood cells in SCT (or SCD) cohorts, limiting our ability to validate our association findings. In addition, our overlap analysis with public resources such as the EWAS and GWAS catalogs is constrained by a dominance of studies derived from European ancestry or high-income populations, potentially overlooking SCT-specific biology in underrepresented groups.

Interpretation of functional relevance is also limited. We annotated CpGs and DMRs to nearby genes based on genomic proximity, but this approach may misrepresent regulatory relationships – particularly for regions distal to transcription start sites or acting through long-range chromatin interactions. Without supporting tissue-specific gene expression or chromatin conformation data, such assignments remain speculative. Our eQTM analysis was performed in a separate sample at a later time point, and included only the subset of 103 DMPs, with a sample size of 726 AA participants, which likely limited statistical power. Moreover, the physical proximity between CpGs and GWAS loci (±10 kb) does not capture long-range chromatin interactions or linkage disequilibrium and therefore does not imply functional regulation.

Finally, our study is cross-sectional, preventing us from distinguishing whether methylation changes are a cause or consequence of other SCT-related traits such as kidney function, hemolysis, or other alterations in hematologic parameters. DNAm was measured using genomic DNA extracted from whole blood, and although we adjusted for estimated leukocyte proportions, (including B cells, NK cells, CD4 T cells, and CD8 T cells), residual confounding by cell composition remains possible. We conducted sensitivity analyses including leukocyte fractions and eGFR as covariates; while associations were generally consistent in the overlapping sample, the reduced sample size led to a loss of power and attenuation of some signals. These results suggest that while cell-type composition and renal function may contribute to variation, they are unlikely to fully explain the observed associations. Together, these limitations highlight the need for future studies with longitudinal designs, diverse cohorts, and matched multi-omic data to more precisely elucidate the epigenetic consequences of SCT.

## Conclusions

We observed that SCT is associated with differential whole blood methylation patterns at 103 CpGs from 83 epigenome-wide loci, with signals at both the β-globin gene cluster locus and other hematopoiesis-relevant loci. SCT was associated with higher epigenetic age using newer generation clocks. Future work should explore these associations in larger datasets and in additional SCT-relevant tissues, such as the kidney. Finally, additional studies that include larger numbers of SCD patients with DNA methylation profiling are needed to further characterize the methylation patterns in blood as well as their relationship to HbF levels and disease severity.

## Supplementary Material

Supplemental Material

## Data Availability

Supplementary Tables S1–S10 associated with this study are publicly available via Zenodo at https://doi.org/10.5281/zenodo.15980411. Individual level data has been submitted or is pending submission to dbGaP (phs000200 and phs001237 WHI, phs000964 and phs000286 JHS, phs001211) or BioLINCC (JHS HLB00891219a); data is also available through study coordinating centers (https://www.whi.org/WHI, https://www.jacksonheartstudy.org/JHS) with approved manuscript proposals.
