# Supplementary figures and images for: Methylation profile of individuals with sickle cell trait
Source: Epigenetics. 2025 Aug 4;20(1):2539234. doi: 10.1080/15592294.2025.2539234 (PMC12323419; doi:10.1080/15592294.2025.2539234)

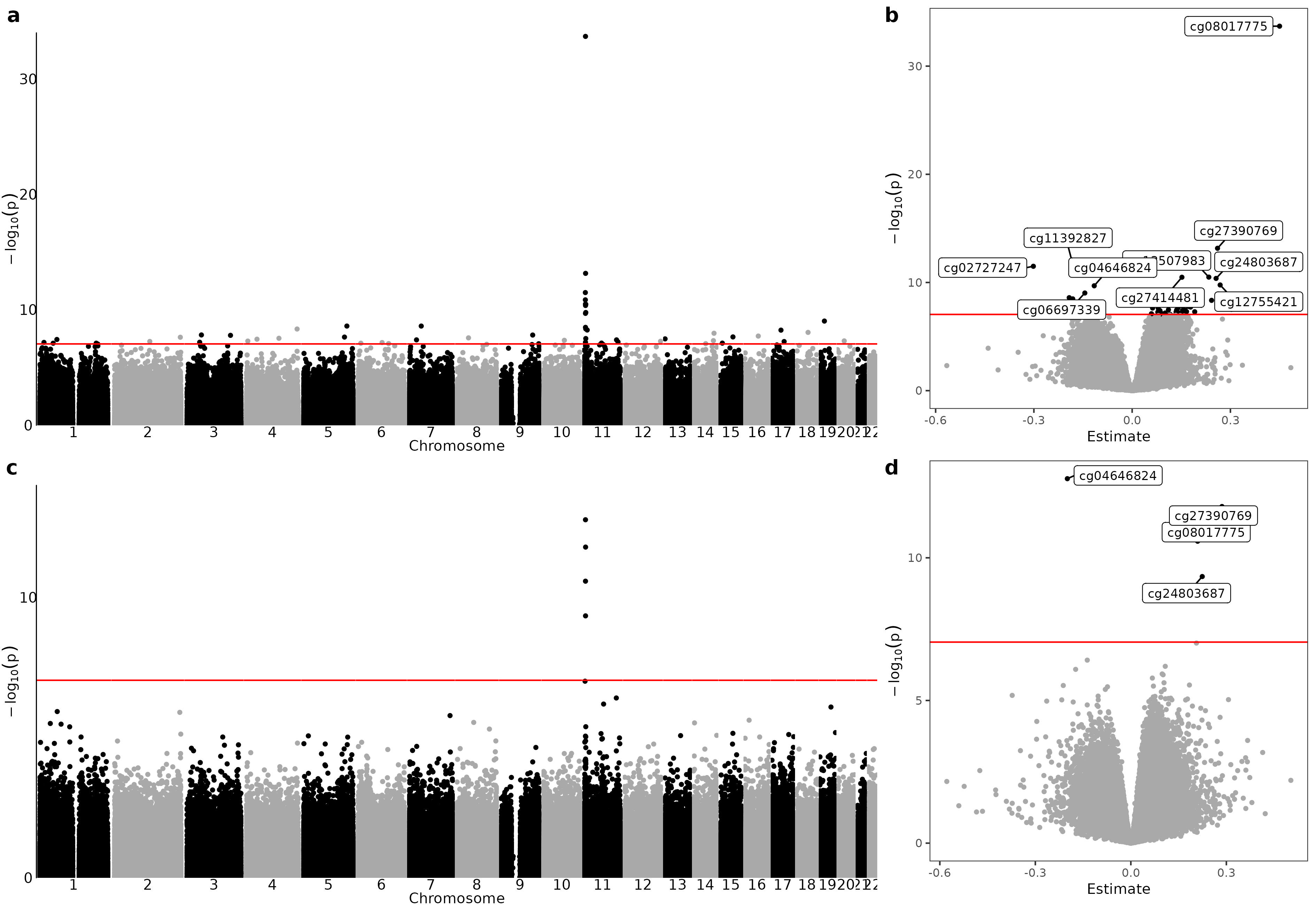

Supplement: Supplemental Material [file KEPI_A_2539234_SM0609.zip › Supplementary files/SupplementalFigure1.jpeg]

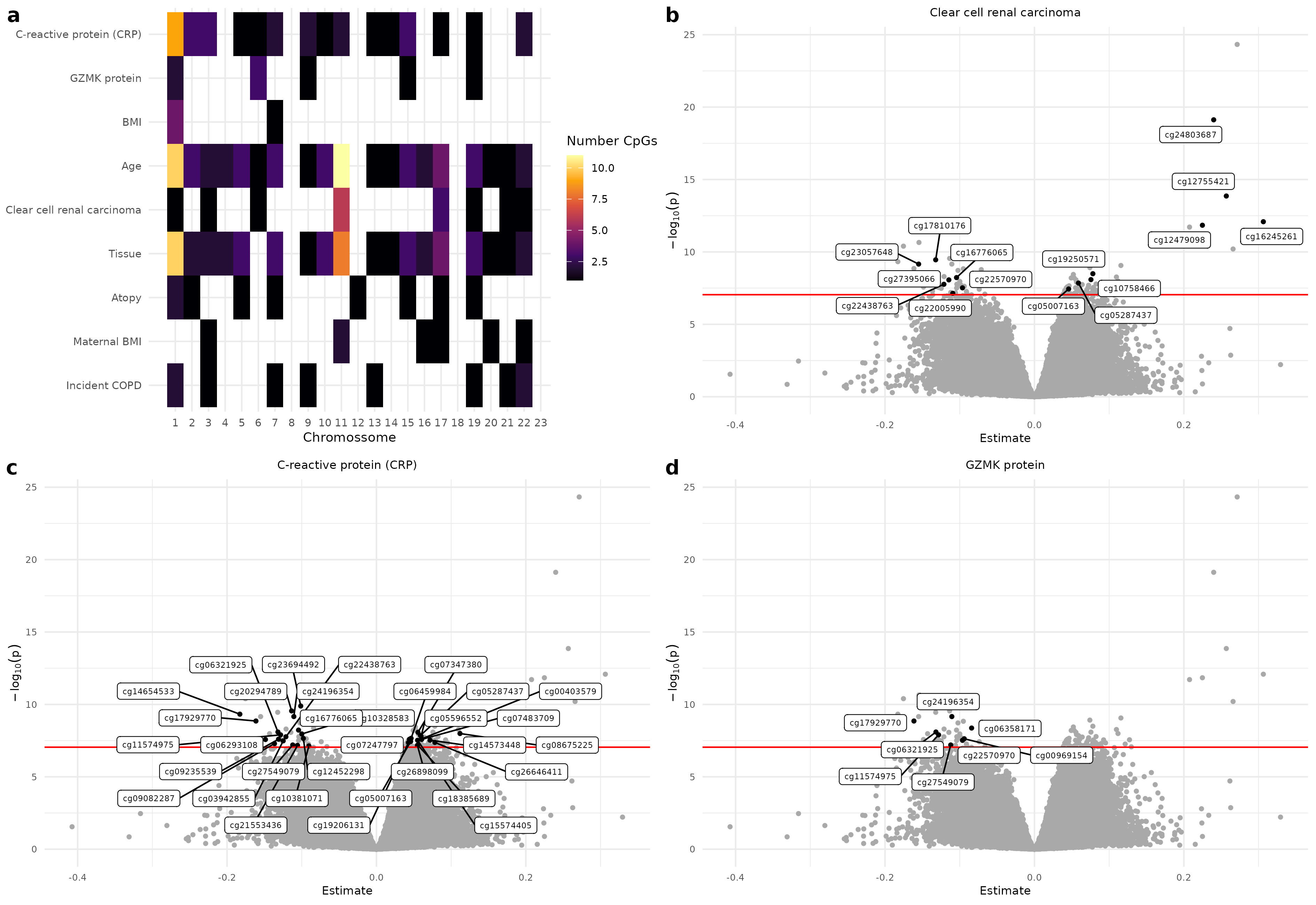

Supplement: Supplemental Material [file KEPI_A_2539234_SM0609.zip › Supplementary files/SupplementalFigure2.jpeg]

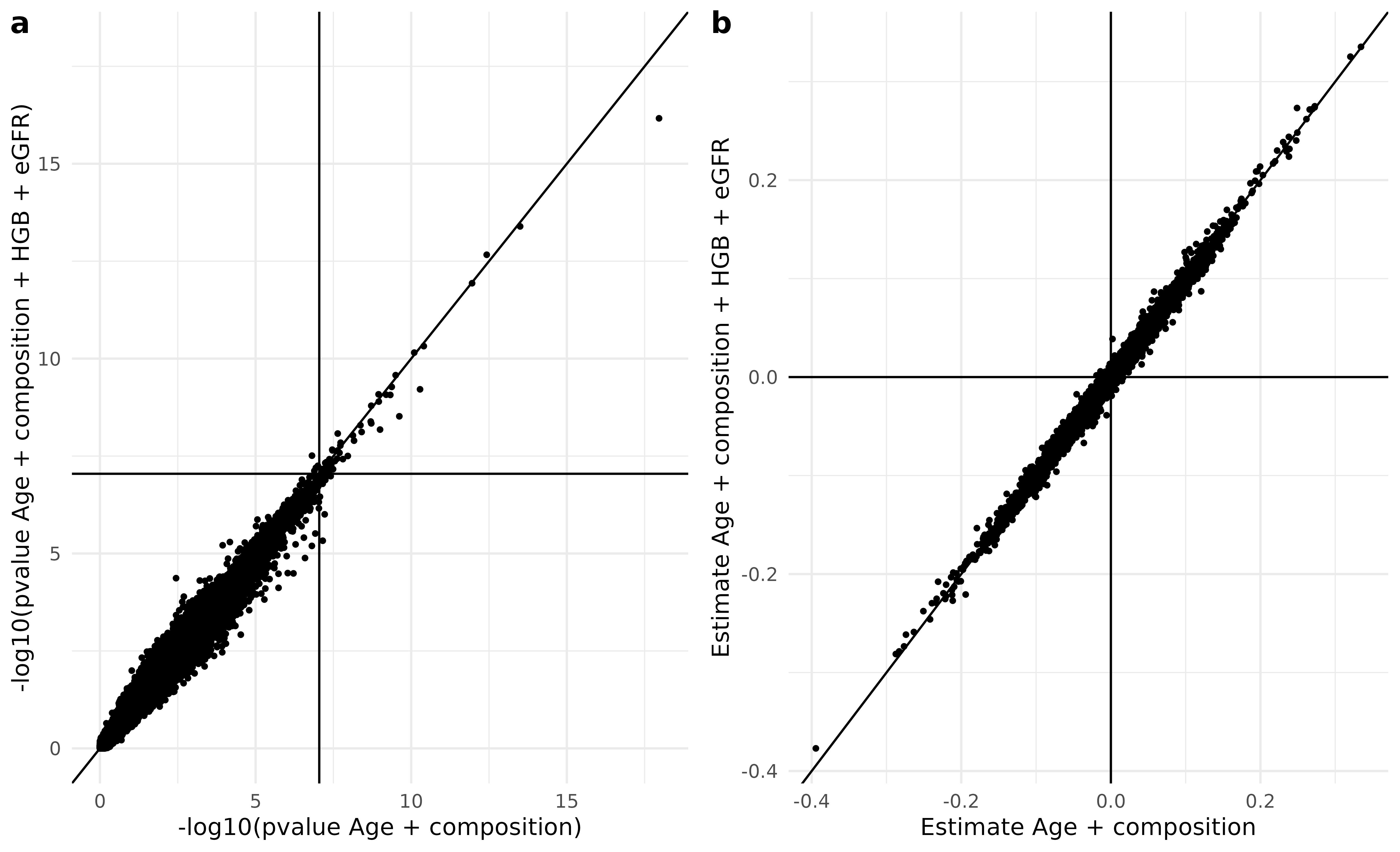

Supplement: Supplemental Material [file KEPI_A_2539234_SM0609.zip › Supplementary files/SupplementalFigure3.jpeg]
