# Supplementary material for: Methylation profile of individuals with sickle cell trait
Source: Epigenetics. 2025 Aug 4;20(1):2539234. doi: 10.1080/15592294.2025.2539234 (PMC12323419; doi:10.1080/15592294.2025.2539234)
Supplement: Supplemental Material [file KEPI_A_2539234_SM0609.zip › Supplementary files/SupplementaryTables.zip!/SupplementalTable1.docx]

| **cpg** | **chr** | **pos (GRCh37)** | **Top CpG in region** | **Estimate** | | | **Standard Error** | | | **p-value** | | | **Relation to Island** | **Transcript region** | **Mapped Genes** |
| --- | --- | --- | --- | --- | --- | --- | --- | --- | --- | --- | --- | --- | --- | --- | --- |
|  |  |  |  | **WHI** | **JHS** | **Meta analysis** | **WHI** | **JHS** | **Meta analysis** | **WHI** | **JHS** | **Meta analysis** |  |  |  |
| cg10328583 | 1 | 6551073 | cg10328583 | -0.114 | -0.078 | -0.100 | 0.022 | 0.027 | 0.017 | 2.60E-07 | 3.80E-03 | 1.10E-08 | Island | Gene body and Promoter | PLEKHG5 |
| cg03562766 | 1 | 23698478 | cg06196597 | 0.087 | 0.068 | 0.078 | 0.021 | 0.019 | 0.014 | 3.80E-05 | 3.40E-04 | 3.40E-08 | S Shelf | Intergenic |  |
| cg06196597 | 1 | 24435477 | cg06196597 | 0.086 | 0.069 | 0.079 | 0.019 | 0.021 | 0.014 | 9.90E-06 | 1.00E-03 | 2.90E-08 | Open Sea | Promoter | MYOM3 |
| cg07247797 | 1 | 41746150 | cg07247797 | 0.050 | 0.034 | 0.044 | 0.010 | 0.012 | 0.008 | 1.10E-06 | 6.20E-03 | 2.50E-08 | Open Sea | Intergenic | RP11-399E6.1 |
| cg22438763 | 1 | 47900256 | cg22438763 | -0.129 | -0.108 | -0.121 | 0.030 | 0.031 | 0.021 | 1.60E-05 | 5.00E-04 | 1.70E-08 | Island | Gene body and Promoter | FOXD2; MGC12982; RP11-511I2.5 |
| cg06358171 | 1 | 54822008 | cg06358171 | -0.089 | -0.078 | -0.084 | 0.022 | 0.019 | 0.014 | 5.20E-05 | 4.10E-05 | 4.30E-09 | Island | Gene body | SSBP3 |
| cg19963589 | 1 | 54822103 | cg06358171 | -0.070 | -0.062 | -0.066 | 0.020 | 0.016 | 0.012 | 4.10E-04 | 7.50E-05 | 8.30E-08 | Island | Gene body | SSBP3 |
| cg09235539 | 1 | 65730481 | cg09235539 | -0.153 | -0.111 | -0.131 | 0.035 | 0.032 | 0.024 | 9.20E-06 | 5.10E-04 | 2.50E-08 | N Shore | Promoter | DNAJC6 |
| cg09082287 | 1 | 65730494 | cg09235539 | -0.161 | -0.109 | -0.137 | 0.036 | 0.034 | 0.025 | 9.80E-06 | 1.50E-03 | 5.10E-08 | N Shore | Promoter | DNAJC6 |
| cg03942855 | 1 | 110210925 | cg03942855 | -0.108 | -0.137 | -0.125 | 0.032 | 0.031 | 0.023 | 8.30E-04 | 1.40E-05 | 3.30E-08 | Island | Gene body | GSTM2 |
| cg11574975 | 1 | 112058304 | cg11574975 | -0.132 | -0.115 | -0.128 | 0.029 | 0.037 | 0.023 | 4.00E-06 | 1.70E-03 | 1.30E-08 | Island | Gene body | ADORA3 |
| cg20294789 | 1 | 151513456 | cg20294789 | -0.124 | -0.102 | -0.114 | 0.029 | 0.023 | 0.018 | 1.50E-05 | 9.50E-06 | 2.80E-10 | S Shore | Gene body | TUFT1; RP11-74C1.4 |
| cg27447874 | 1 | 165346015 | cg27447874 | -0.108 | -0.090 | -0.104 | 0.023 | 0.030 | 0.018 | 3.60E-06 | 3.00E-03 | 1.70E-08 | Open Sea | Intergenic |  |
| cg18385689 | 1 | 226136936 | cg18385689 | 0.061 | 0.045 | 0.055 | 0.013 | 0.017 | 0.010 | 2.40E-06 | 7.20E-03 | 6.50E-08 | Open Sea | Intergenic |  |
| cg19206131 | 2 | 26225806 | cg19206131 | 0.049 | 0.036 | 0.043 | 0.010 | 0.012 | 0.008 | 2.80E-06 | 2.30E-03 | 4.30E-08 | Open Sea | Intergenic |  |
| cg23718924 | 2 | 60778617 | cg23718924 | -0.135 | -0.109 | -0.128 | 0.029 | 0.037 | 0.023 | 4.50E-06 | 3.30E-03 | 2.90E-08 | Island | Gene body | BCL11A |
| cg02541582 | 2 | 64069798 | cg02541582 | -0.126 | -0.093 | -0.112 | 0.029 | 0.030 | 0.021 | 1.60E-05 | 2.00E-03 | 8.70E-08 | S Shore | Gene body and Promoter | UGP2 |
| cg16354502 | 2 | 71697410 | cg15574405 | 0.099 | 0.089 | 0.095 | 0.026 | 0.023 | 0.017 | 1.70E-04 | 1.30E-04 | 4.60E-08 | S Shelf | Gene body | DYSF |
| cg15574405 | 2 | 71776472 | cg15574405 | 0.060 | 0.046 | 0.055 | 0.013 | 0.015 | 0.010 | 4.30E-06 | 2.50E-03 | 3.00E-08 | Open Sea | Gene body | DYSF |
| cg07347380 | 2 | 232799969 | cg07347380 | 0.057 | 0.056 | 0.056 | 0.012 | 0.015 | 0.010 | 5.70E-06 | 2.20E-04 | 8.50E-09 | Open Sea | Intergenic |  |
| cg05287437 | 3 | 16215248 | cg05287437 | 0.053 | 0.065 | 0.059 | 0.014 | 0.015 | 0.010 | 1.50E-04 | 2.40E-05 | 1.40E-08 | Open Sea | Promoter | GALNTL2 |
| cg21553436 | 3 | 33701149 | cg21553436 | -0.112 | -0.097 | -0.106 | 0.027 | 0.029 | 0.020 | 2.90E-05 | 6.90E-04 | 6.80E-08 | Open Sea | Gene body and Promoter | CLASP2 |
| cg25367572 | 3 | 52926875 | cg25367572 | 0.136 | 0.109 | 0.121 | 0.033 | 0.030 | 0.022 | 4.00E-05 | 3.20E-04 | 5.50E-08 | Open Sea | Gene body | TMEM110-MUSTN1; TMEM110 |
| cg14654533 | 3 | 131080755 | cg14654533 | -0.163 | -0.217 | -0.183 | 0.037 | 0.049 | 0.029 | 8.70E-06 | 9.50E-06 | 4.70E-10 | Island | Gene body | NUDT16P; RP11-933H2.4 |
| cg01944478 | 3 | 134363967 | cg01944478 | 0.052 | 0.041 | 0.048 | 0.012 | 0.014 | 0.009 | 7.40E-06 | 2.90E-03 | 7.40E-08 | Open Sea | Gene body | KY |
| cg11843333 | 4 | 15719138 | cg11843333 | 0.088 | 0.089 | 0.089 | 0.025 | 0.022 | 0.016 | 4.70E-04 | 3.40E-05 | 5.10E-08 | Open Sea | Gene body | BST1 |
| cg15984718 | 4 | 96470349 | cg15984718 | -0.114 | -0.083 | -0.099 | 0.026 | 0.024 | 0.018 | 8.50E-06 | 5.80E-04 | 1.90E-08 | Island | Promoter | UNC5C |
| cg01872122 | 5 | 16940481 | cg01872122 | 0.124 | 0.109 | 0.116 | 0.030 | 0.024 | 0.019 | 2.80E-05 | 8.70E-06 | 8.70E-10 | S Shelf | Intergenic |  |
| cg12193298 | 5 | 77870946 | cg12193298 | 0.077 | 0.061 | 0.070 | 0.017 | 0.019 | 0.013 | 8.00E-06 | 1.60E-03 | 4.20E-08 | Open Sea | Promoter | LHFPL2 |
| cg04476846 | 5 | 78791415 | cg12193298 | 0.073 | 0.095 | 0.085 | 0.022 | 0.022 | 0.016 | 1.30E-03 | 1.70E-05 | 8.10E-08 | Open Sea | Gene body | HOMER1 |
| cg26898099 | 5 | 95192949 | cg26898099 | 0.061 | 0.044 | 0.055 | 0.013 | 0.017 | 0.010 | 2.00E-06 | 7.60E-03 | 6.80E-08 | Open Sea | Gene body | C5orf27 |
| cg23109996 | 5 | 142023934 | cg23109996 | 0.079 | 0.052 | 0.068 | 0.016 | 0.018 | 0.013 | 1.10E-06 | 3.90E-03 | 6.40E-08 | Open Sea | Promoter | FGF1 |
| cg15407091 | 5 | 148211186 | cg15407091 | 0.047 | 0.064 | 0.053 | 0.011 | 0.015 | 0.009 | 3.90E-05 | 2.50E-05 | 6.30E-09 | Open Sea | Intergenic |  |
| cg04254052 | 5 | 170029226 | cg04254052 | 0.059 | 0.060 | 0.059 | 0.015 | 0.016 | 0.011 | 9.30E-05 | 1.20E-04 | 4.70E-08 | Open Sea | Gene body and Promoter | KCNIP1 |
| cg24196354 | 6 | 13445503 | cg24196354 | -0.111 | -0.106 | -0.111 | 0.026 | 0.024 | 0.018 | 2.30E-05 | 1.50E-05 | 6.90E-10 | Open Sea | Gene body and Promoter | GFOD1 |
| cg00969154 | 6 | 15401024 | cg00969154 | -0.094 | -0.092 | -0.094 | 0.022 | 0.026 | 0.017 | 1.70E-05 | 4.30E-04 | 2.40E-08 | Open Sea | Gene body and Promoter | JARID2 |
| cg22570970 | 6 | 15401067 | cg00969154 | -0.099 | -0.091 | -0.096 | 0.023 | 0.027 | 0.017 | 1.50E-05 | 6.60E-04 | 3.00E-08 | Open Sea | Gene body | JARID2 |
| cg23694492 | 7 | 2672929 | cg23694492 | -0.107 | -0.093 | -0.101 | 0.021 | 0.024 | 0.016 | 4.40E-07 | 9.00E-05 | 1.30E-10 | S Shore | Gene body | TTYH3 |
| cg14573448 | 7 | 27165187 | cg14573448 | 0.061 | 0.084 | 0.072 | 0.017 | 0.020 | 0.013 | 4.20E-04 | 2.10E-05 | 2.90E-08 | S Shore | Promoter | HOXA3; HOXA-AS2 |
| cg04391064 | 7 | 43797839 | cg04391064 | -0.192 | -0.129 | -0.175 | 0.032 | 0.047 | 0.027 | 2.60E-09 | 6.30E-03 | 4.00E-11 | Island | Promoter | BLVRA |
| cg03696781 | 7 | 45742854 | cg03696781 | 0.052 | 0.051 | 0.052 | 0.013 | 0.015 | 0.010 | 5.40E-05 | 5.90E-04 | 8.00E-08 | Open Sea | Gene body | ADCY1 |
| cg20586431 | 7 | 116963479 | cg20586431 | -0.135 | -0.152 | -0.143 | 0.030 | 0.043 | 0.025 | 9.30E-06 | 4.90E-04 | 1.10E-08 | Island | Promoter | WNT2 |
| cg05290748 | 8 | 21668909 | cg05290748 | 0.057 | 0.044 | 0.052 | 0.011 | 0.014 | 0.009 | 5.10E-07 | 1.90E-03 | 3.60E-09 | Open Sea | Intergenic | GFRA2 |
| cg02495935 | 8 | 37690341 | cg02495935 | 0.086 | 0.078 | 0.082 | 0.023 | 0.020 | 0.015 | 1.50E-04 | 7.60E-05 | 4.20E-08 | Open Sea | Gene body | ADGRA2 |
| cg00676161 | 9 | 116620364 | cg00676161 | 0.074 | 0.098 | 0.085 | 0.020 | 0.025 | 0.016 | 2.30E-04 | 7.40E-05 | 5.70E-08 | Open Sea | Intergenic |  |
| cg00403579 | 9 | 124533992 | cg00403579 | 0.069 | 0.050 | 0.060 | 0.015 | 0.016 | 0.011 | 5.30E-06 | 1.30E-03 | 2.40E-08 | N Shore | Gene body | DAB2IP |
| cg06321925 | 9 | 127420734 | cg06321925 | -0.151 | -0.106 | -0.132 | 0.033 | 0.032 | 0.023 | 3.80E-06 | 9.20E-04 | 8.10E-09 | Open Sea | Gene body | MIR181A2HG; NR6A1 |
| cg07483709 | 10 | 29439573 | cg07483709 | 0.067 | 0.051 | 0.061 | 0.014 | 0.017 | 0.011 | 3.30E-06 | 2.10E-03 | 2.60E-08 | Open Sea | Intergenic |  |
| cg15279476 | 10 | 62704447 | cg15279476 | -0.073 | -0.064 | -0.071 | 0.015 | 0.019 | 0.012 | 1.10E-06 | 9.10E-04 | 1.70E-09 | S Shore | Gene body and Promoter | RHOBTB1 |
| cg15334916 | 10 | 71563776 | cg15334916 | 0.082 | 0.064 | 0.073 | 0.019 | 0.018 | 0.013 | 1.30E-05 | 3.40E-04 | 2.00E-08 | S Shore | Gene body | COL13A1 |
| cg04247230 | 11 | 1074009 | cg04247230 | 0.090 | 0.067 | 0.076 | 0.020 | 0.019 | 0.014 | 1.10E-05 | 6.30E-04 | 6.90E-08 | Open Sea | Promoter | MUC2 |
| cg13145504 | 11 | 2594840 | cg23959735 | 0.142 | 0.109 | 0.130 | 0.030 | 0.042 | 0.024 | 1.90E-06 | 8.80E-03 | 8.50E-08 | N Shore | Gene body | KCNQ1 |
| cg23959735 | 11 | 3247253 | cg23959735 | 0.079 | 0.085 | 0.082 | 0.019 | 0.022 | 0.015 | 4.60E-05 | 1.10E-04 | 1.70E-08 | Open Sea | Intergenic | AC109309.4 |
| cg19250571 | 11 | 4869963 | cg27390769 | 0.090 | 0.064 | 0.078 | 0.017 | 0.018 | 0.013 | 8.40E-08 | 4.40E-04 | 3.20E-09 | Open Sea | Intergenic | OR51S1; MMP26 |
| cg13507983 | 11 | 5128882 | cg27390769 | 0.234 | 0.178 | 0.208 | 0.035 | 0.040 | 0.029 | 3.20E-11 | 8.90E-06 | 1.90E-12 | Open Sea | Intergenic | AC113331.9 |
| cg07570010 | 11 | 5249270 | cg27390769 | 0.108 | 0.119 | 0.110 | 0.023 | 0.033 | 0.019 | 2.70E-06 | 3.00E-04 | 5.40E-09 | Open Sea | Promoter | HBB |
| cg24803687 | 11 | 5462916 | cg27390769 | 0.256 | 0.224 | 0.240 | 0.039 | 0.036 | 0.026 | 4.20E-11 | 4.50E-10 | 7.50E-20 | Open Sea | Promoter | OR51I1; HBG2; HBE1; AC104389.28 |
| cg22694445 | 11 | 5570731 | cg27390769 | 0.120 | 0.142 | 0.127 | 0.030 | 0.035 | 0.023 | 6.50E-05 | 5.90E-05 | 2.80E-08 | Open Sea | Intergenic | HBG2 |
| cg27390769 | 11 | 5621691 | cg27390769 | 0.261 | 0.286 | 0.271 | 0.035 | 0.040 | 0.026 | 6.90E-14 | 1.60E-12 | 4.70E-25 | Open Sea | Gene body and Promoter | TRIM6; TRIM6-TRIM34; HBG2; AC015691.13 |
| cg16776065 | 11 | 6340486 | cg27390769 | -0.095 | -0.119 | -0.104 | 0.022 | 0.031 | 0.018 | 1.60E-05 | 1.10E-04 | 5.90E-09 | Island | Gene body | PRKCDBP/CAVIN3 |
| cg04333575 | 11 | 6340890 | cg27390769 | -0.115 | -0.080 | -0.095 | 0.023 | 0.017 | 0.016 | 5.10E-07 | 4.10E-06 | 1.50E-09 | Island | Gene body | PRKCDBP/CAVIN3 |
| cg12755421 | 11 | 6342214 | cg27390769 | 0.269 | 0.234 | 0.257 | 0.042 | 0.055 | 0.033 | 1.70E-10 | 2.30E-05 | 1.40E-14 | S Shore | Promoter | PRKCDBP/CAVIN3 |
| cg12479098 | 11 | 6342231 | cg27390769 | 0.242 | 0.197 | 0.225 | 0.041 | 0.050 | 0.032 | 4.50E-09 | 8.00E-05 | 1.40E-12 | S Shore | Promoter | PRKCDBP/CAVIN3 |
| cg03701853 | 11 | 6342337 | cg27390769 | 0.276 | 0.247 | 0.266 | 0.053 | 0.063 | 0.041 | 2.40E-07 | 8.80E-05 | 6.30E-11 | S Shore | Promoter | PRKCDBP/CAVIN3 |
| cg16245261 | 11 | 6342357 | cg27390769 | 0.303 | 0.307 | 0.306 | 0.054 | 0.069 | 0.043 | 3.10E-08 | 9.30E-06 | 8.20E-13 | S Shore | Promoter | PRKCDBP/CAVIN3 |
| cg17614165 | 11 | 47468663 | cg17614165 | 0.084 | 0.066 | 0.074 | 0.017 | 0.017 | 0.012 | 1.10E-06 | 1.30E-04 | 1.20E-09 | Open Sea | Gene body | RAPSN |
| cg05596552 | 11 | 64826323 | cg05596552 | 0.059 | 0.060 | 0.060 | 0.014 | 0.016 | 0.011 | 4.80E-05 | 1.00E-04 | 1.60E-08 | Open Sea | Promoter | NAALADL1 |
| cg07117751 | 11 | 76811860 | cg07117751 | 0.077 | 0.057 | 0.065 | 0.016 | 0.017 | 0.012 | 2.60E-06 | 8.50E-04 | 7.60E-08 | N Shelf | Gene body | CAPN5 |
| cg16639798 | 12 | 6893565 | cg16639798 | 0.093 | 0.065 | 0.080 | 0.021 | 0.021 | 0.015 | 7.30E-06 | 2.10E-03 | 6.10E-08 | Open Sea | Intergenic |  |
| cg12783132 | 12 | 54943700 | cg12783132 | -0.089 | -0.061 | -0.074 | 0.021 | 0.017 | 0.013 | 2.80E-05 | 4.40E-04 | 3.80E-08 | Open Sea | Gene body and Promoter | PDE1B |
| cg06293108 | 13 | 110438256 | cg06293108 | -0.151 | -0.141 | -0.149 | 0.036 | 0.040 | 0.027 | 2.60E-05 | 4.50E-04 | 2.70E-08 | Island | Intergenic | IRS2 |
| cg08675225 | 14 | 58606095 | cg08675225 | 0.107 | 0.115 | 0.112 | 0.025 | 0.032 | 0.020 | 1.70E-05 | 2.80E-04 | 1.00E-08 | Open Sea | Promoter | C14orf37 |
| cg00607599 | 14 | 73726726 | cg00607599 | 0.073 | 0.056 | 0.063 | 0.018 | 0.015 | 0.012 | 4.80E-05 | 2.90E-04 | 6.90E-08 | Open Sea | Gene body | PAPLN |
| cg14327296 | 14 | 101051064 | cg14327296 | 0.043 | 0.032 | 0.039 | 0.009 | 0.011 | 0.007 | 7.50E-06 | 3.80E-03 | 8.10E-08 | N Shelf | Intergenic |  |
| cg27549079 | 15 | 64566942 | cg27549079 | -0.116 | -0.106 | -0.112 | 0.029 | 0.029 | 0.021 | 6.20E-05 | 3.50E-04 | 6.30E-08 | Open Sea | Gene body | CSNK1G1; CTD-2116N17.1 |
| cg12452298 | 15 | 67134587 | cg12452298 | -0.094 | -0.104 | -0.098 | 0.021 | 0.032 | 0.017 | 7.30E-06 | 1.00E-03 | 2.20E-08 | N Shore | Intergenic |  |
| cg15084949 | 15 | 70128869 | cg15084949 | 0.049 | 0.049 | 0.049 | 0.011 | 0.014 | 0.008 | 5.80E-06 | 2.70E-04 | 6.30E-09 | Open Sea | Gene body | LINC00593; C15orf50 |
| cg10381071 | 15 | 70391035 | cg15084949 | -0.099 | -0.079 | -0.090 | 0.022 | 0.026 | 0.017 | 7.10E-06 | 2.50E-03 | 7.40E-08 | Island | Promoter | TLE3 |
| cg26677315 | 15 | 97038051 | cg26677315 | -0.133 | -0.104 | -0.121 | 0.027 | 0.032 | 0.021 | 1.00E-06 | 1.10E-03 | 5.00E-09 | Open Sea | Intergenic |  |
| cg07892295 | 15 | 98503714 | cg07892295 | -0.116 | -0.086 | -0.102 | 0.025 | 0.023 | 0.017 | 3.30E-06 | 2.40E-04 | 1.90E-09 | Island | Promoter | ARRDC4 |
| cg04839677 | 16 | 3048362 | cg04839677 | 0.045 | 0.041 | 0.043 | 0.010 | 0.012 | 0.008 | 1.20E-05 | 5.30E-04 | 3.10E-08 | Open Sea | Intergenic | LINC00514 |
| cg00089783 | 16 | 16261857 | cg00089783 | 0.074 | 0.104 | 0.093 | 0.025 | 0.022 | 0.017 | 3.50E-03 | 2.40E-06 | 2.80E-08 | Open Sea | Gene body | ABCC6 |
| cg23009806 | 16 | 46771757 | cg23009806 | 0.070 | 0.048 | 0.059 | 0.015 | 0.015 | 0.011 | 4.60E-06 | 2.00E-03 | 4.80E-08 | Open Sea | Gene body and Promoter | MYLK3 |
| cg05261851 | 16 | 68001206 | cg05261851 | 0.042 | 0.038 | 0.041 | 0.009 | 0.013 | 0.007 | 5.10E-06 | 2.50E-03 | 4.70E-08 | N Shore | Gene body and Promoter | SLC12A4 |
| cg07104525 | 17 | 18020727 | cg07104525 | 0.088 | 0.061 | 0.074 | 0.020 | 0.018 | 0.014 | 1.60E-05 | 9.00E-04 | 7.50E-08 | N Shore | Promoter | MYO15A |
| cg10758466 | 17 | 41974087 | cg10758466 | 0.077 | 0.075 | 0.076 | 0.017 | 0.020 | 0.013 | 9.80E-06 | 1.50E-04 | 8.20E-09 | N Shelf | Gene body | MPP2 |
| cg27395066 | 17 | 43221220 | cg27395066 | -0.123 | -0.099 | -0.115 | 0.024 | 0.035 | 0.020 | 4.30E-07 | 4.50E-03 | 8.50E-09 | N Shore | Gene body | ACBD4 |
| cg22005990 | 17 | 46659939 | cg22005990 | -0.118 | -0.094 | -0.109 | 0.026 | 0.032 | 0.020 | 6.10E-06 | 3.80E-03 | 7.30E-08 | Island | Intergenic | HOXB3; RP11-357H14.16; HOXB-AS3 |
| cg08691490 | 17 | 48735420 | cg08691490 | 0.048 | 0.037 | 0.044 | 0.010 | 0.013 | 0.008 | 5.10E-06 | 3.90E-03 | 5.30E-08 | Open Sea | Gene body | ABCC3 |
| cg04318345 | 17 | 72400257 | cg04318345 | 0.074 | 0.051 | 0.064 | 0.016 | 0.016 | 0.011 | 2.60E-06 | 1.70E-03 | 1.80E-08 | Open Sea | Intergenic |  |
| cg08816988 | 17 | 76893060 | cg08816988 | 0.051 | 0.053 | 0.052 | 0.013 | 0.014 | 0.010 | 1.10E-04 | 1.90E-04 | 8.80E-08 | Open Sea | Gene body | TIMP2 |
| cg06459984 | 17 | 79381472 | cg06459984 | 0.044 | 0.052 | 0.046 | 0.010 | 0.014 | 0.008 | 2.40E-05 | 1.60E-04 | 2.20E-08 | S Shore | Gene body | BAHCC1 |
| cg06152601 | 17 | 80444748 | cg06152601 | 0.044 | 0.063 | 0.054 | 0.014 | 0.014 | 0.010 | 1.50E-03 | 9.30E-06 | 7.40E-08 | Open Sea | Gene body | NARF |
| cg13957558 | 19 | 11435095 | cg13957558 | -0.117 | -0.077 | -0.104 | 0.024 | 0.030 | 0.019 | 1.30E-06 | 1.00E-02 | 3.10E-08 | Open Sea | Gene body | TSPAN16; RAB3D |
| cg17810176 | 19 | 36035831 | cg17810176 | -0.134 | -0.121 | -0.132 | 0.027 | 0.034 | 0.021 | 4.80E-07 | 4.50E-04 | 3.50E-10 | N Shore | Gene body and Promoter | GAPDHS; TMEM147; AD000090.2 |
| cg04209573 | 19 | 42070764 | cg01265982 | -0.107 | -0.069 | -0.091 | 0.021 | 0.023 | 0.017 | 7.80E-07 | 3.50E-03 | 5.40E-08 | Open Sea | Promoter | CEACAM21; AC005626.1 |
| cg01265982 | 19 | 42070875 | cg01265982 | -0.137 | -0.173 | -0.155 | 0.031 | 0.035 | 0.023 | 8.40E-06 | 8.10E-07 | 2.20E-11 | Open Sea | Promoter | CEACAM21; AC005626.1 |
| cg17929770 | 19 | 46318514 | cg17929770 | -0.165 | -0.150 | -0.161 | 0.037 | 0.038 | 0.027 | 9.40E-06 | 8.90E-05 | 1.40E-09 | Island | Promoter | RSPH6A |
| cg15407257 | 20 | 23621136 | cg15407257 | 0.055 | 0.056 | 0.056 | 0.013 | 0.014 | 0.010 | 4.60E-05 | 1.10E-04 | 1.20E-08 | S Shelf | Intergenic |  |
| cg23057648 | 21 | 40984763 | cg23057648 | -0.146 | -0.155 | -0.155 | 0.033 | 0.039 | 0.025 | 8.70E-06 | 7.00E-05 | 6.90E-10 | Open Sea | Promoter | C21orf88; B3GALT5 |
| cg26646411 | 22 | 19841374 | cg26646411 | 0.070 | 0.092 | 0.078 | 0.018 | 0.024 | 0.014 | 9.60E-05 | 1.10E-04 | 4.20E-08 | N Shore | Promoter | GNB1L; C22orf29 |
| cg05007163 | 22 | 39266069 | cg05007163 | 0.051 | 0.037 | 0.046 | 0.011 | 0.013 | 0.008 | 2.30E-06 | 3.90E-03 | 3.60E-08 | N Shore | Gene body | CBX6 |
| cg14596330 | 22 | 51176615 | cg14596330 | 0.041 | 0.044 | 0.043 | 0.010 | 0.012 | 0.008 | 4.30E-05 | 2.20E-04 | 3.10E-08 | Open Sea | Promoter | ACR; AC000036.4 |
