# Supplementary material for: Methylation profile of individuals with sickle cell trait
Source: Epigenetics. 2025 Aug 4;20(1):2539234. doi: 10.1080/15592294.2025.2539234 (PMC12323419; doi:10.1080/15592294.2025.2539234)
Supplement: Supplemental Material [file KEPI_A_2539234_SM0609.zip › Supplementary files/SupplementaryTables.zip!/SupplementalTable2.docx]

| **cpg** | **chr** | **pos** | **Relation to island** | **Genes** | **Estimate** | | | **p-value** | | |
| --- | --- | --- | --- | --- | --- | --- | --- | --- | --- | --- |
|  |  |  |  |  | **Original** | **Original Same sample** | **Sensitivity** | **Original** | **Original Same sample** | **Sensitivity** |
| cg03484312 | 1 | 49216204 | OpenSea | BEND5, AGBL4 | 0.05 | 0.048 | 0.047 | 8.33E-07 | **3.87E-08** | 1.04E-07 |
| cg03562766 | 1 | 23698478 | S_Shelf |  | 0.078 | 0.078 | 0.078 | **3.44E-08** | **6.74E-08** | 1.02E-07 |
| cg06196597 | 1 | 24435477 | OpenSea | MYOM3 | 0.079 | 0.078 | 0.078 | **2.85E-08** | 9.13E-08 | 1.07E-07 |
| cg07247797 | 1 | 41746150 | OpenSea | RP11-399E6.1 | 0.044 | 0.043 | 0.043 | **2.54E-08** | 1.81E-07 | 7.92E-07 |
| cg09082287 | 1 | 65730494 | N_Shore | DNAJC6 | -0.137 | -0.133 | -0.133 | **5.07E-08** | 3.32E-07 | 2.00E-06 |
| cg09235539 | 1 | 65730481 | N_Shore | DNAJC6 | -0.131 | -0.129 | -0.132 | **2.54E-08** | 9.28E-08 | 4.78E-07 |
| cg10328583 | 1 | 6551073 | Island | PLEKHG5 | -0.1 | -0.1 | -0.1 | **1.10E-08** | 9.25E-08 | 6.93E-07 |
| cg18385689 | 1 | 2.26E+08 | OpenSea |  | 0.055 | 0.051 | 0.049 | **6.47E-08** | 1.28E-06 | 3.97E-06 |
| cg19963589 | 1 | 54822103 | Island | SSBP3 | -0.066 | -0.064 | -0.065 | **8.25E-08** | 4.69E-07 | 3.76E-07 |
| cg22438763 | 1 | 47900256 | Island | RP11-511I2.5, FOXD2, MGC12982 | -0.121 | -0.115 | -0.114 | **1.72E-08** | 1.74E-07 | 2.74E-07 |
| cg27447874 | 1 | 1.65E+08 | OpenSea |  | -0.104 | -0.096 | -0.097 | **1.69E-08** | 4.06E-07 | 3.22E-07 |
| cg02541582 | 2 | 64069798 | S_Shore | UGP2 | -0.112 | -0.118 | -0.116 | **8.74E-08** | **5.43E-08** | 1.04E-07 |
| cg15574405 | 2 | 71776472 | OpenSea | DYSF | 0.055 | 0.055 | 0.055 | **2.99E-08** | **6.08E-08** | 9.64E-08 |
| cg16354502 | 2 | 71697410 | S_Shelf | DYSF | 0.095 | 0.095 | 0.095 | **4.55E-08** | 9.25E-08 | 1.31E-07 |
| cg19206131 | 2 | 26225806 | OpenSea |  | 0.043 | 0.043 | 0.041 | **4.32E-08** | 1.21E-07 | 3.07E-06 |
| cg23718924 | 2 | 60778617 | Island | BCL11A | -0.128 | -0.124 | -0.122 | **2.86E-08** | 1.75E-07 | 3.39E-07 |
| cg01944478 | 3 | 1.34E+08 | OpenSea | EPHB1, KY | 0.048 | 0.048 | 0.048 | **7.41E-08** | 1.47E-07 | 1.89E-07 |
| cg21553436 | 3 | 33701149 | OpenSea | CLASP2 | -0.106 | -0.103 | -0.103 | **6.75E-08** | 2.77E-07 | 3.39E-07 |
| cg15984718 | 4 | 96470349 | Island | UNC5C | -0.099 | -0.098 | -0.099 | **1.91E-08** | **6.03E-08** | 9.89E-07 |
| cg03637066 | 5 | 913111 | S_Shore | TRIP13 | 0.049 | 0.053 | 0.055 | 7.85E-07 | 1.29E-07 | **7.57E-08** |
| cg04476846 | 5 | 78791415 | OpenSea | HOMER1 | 0.085 | 0.083 | 0.08 | **8.14E-08** | 3.57E-07 | 9.85E-07 |
| cg12193298 | 5 | 77870946 | OpenSea | LHFPL2 | 0.07 | 0.069 | 0.069 | **4.16E-08** | 1.70E-07 | 1.72E-07 |
| cg23109996 | 5 | 1.42E+08 | OpenSea | FGF1, AC005592.2 | 0.068 | 0.066 | 0.066 | **6.42E-08** | **8.99E-08** | 1.23E-07 |
| cg26898099 | 5 | 95192949 | OpenSea | C5orf27 | 0.055 | 0.051 | 0.051 | **6.81E-08** | 1.08E-06 | 1.14E-06 |
| cg22570970 | 6 | 15401067 | OpenSea | JARID2 | -0.096 | -0.095 | -0.098 | **3.03E-08** | 1.16E-07 | **6.33E-08** |
| cg03696781 | 7 | 45742854 | OpenSea | ADCY1 | 0.052 | 0.052 | 0.05 | **8.04E-08** | 2.32E-07 | 6.90E-07 |
| cg04391064 | 7 | 43797839 | Island | BLVRA | -0.175 | -0.168 | -0.165 | **4.01E-11** | 1.55E-07 | 6.38E-06 |
| cg12431517 | 7 | 26428084 | OpenSea |  | 0.132 | 0.129 | 0.134 | 1.39E-06 | **2.30E-08** | **8.36E-09** |
| cg14573448 | 7 | 27165187 | S_Shore | HOXA3, HOXA-AS2 | 0.072 | 0.07 | 0.067 | **2.93E-08** | 1.87E-07 | 5.60E-07 |
| cg00403579 | 9 | 1.25E+08 | N_Shore | DAB2IP | 0.06 | 0.058 | 0.056 | **2.40E-08** | 1.28E-07 | 3.58E-07 |
| cg00676161 | 9 | 1.17E+08 | OpenSea |  | 0.085 | 0.085 | 0.086 | **5.68E-08** | 1.32E-07 | 1.09E-07 |
| cg07483709 | 10 | 29439573 | OpenSea |  | 0.061 | 0.059 | 0.058 | **2.60E-08** | 1.14E-07 | 2.10E-07 |
| cg27423692 | 10 | 1.35E+08 | S_Shore |  | 0.079 | 0.087 | 0.085 | 1.01E-05 | **6.98E-08** | 1.63E-07 |
| cg04247230 | 11 | 1074009 | OpenSea | MUC2 | 0.076 | 0.078 | 0.077 | **6.94E-08** | **7.76E-08** | 1.07E-07 |
| cg07117751 | 11 | 76811860 | N_Shelf | CAPN5 | 0.065 | 0.069 | 0.067 | **7.63E-08** | 1.15E-04 | 1.31E-04 |
| cg13145504 | 11 | 2594840 | N_Shore | KCNQ1 | 0.13 | 0.13 | 0.13 | **8.51E-08** | 2.20E-07 | 2.35E-07 |
| cg16776065 | 11 | 6340486 | Island | CAVIN3 | -0.104 | -0.099 | -0.101 | **5.93E-09** | 9.97E-08 | **5.63E-08** |
| cg19458497 | 11 | 63403371 | OpenSea | ATL3 | 0.035 | 0.037 | 0.039 | 2.65E-07 | 1.54E-07 | **3.09E-08** |
| cg22694445 | 11 | 5570731 | OpenSea | HBG2 | 0.127 | 0.123 | 0.124 | **2.76E-08** | 1.94E-07 | 1.54E-07 |
| cg23326785 | 11 | 1.18E+08 | N_Shore | TTC36, TMEM25, RP11-770J1.3 | -0.113 | -0.121 | -0.122 | 5.74E-06 | **1.10E-09** | **1.26E-09** |
| cg23959735 | 11 | 3247253 | OpenSea | AC109309.4 | 0.082 | 0.079 | 0.077 | **1.65E-08** | 1.38E-07 | 3.24E-07 |
| cg12783132 | 12 | 54943700 | OpenSea | PDE1B | -0.074 | -0.073 | -0.072 | **3.77E-08** | 1.40E-07 | 2.34E-07 |
| cg16639798 | 12 | 6893565 | OpenSea |  | 0.08 | 0.078 | 0.077 | **6.13E-08** | 2.34E-07 | 4.66E-07 |
| cg00607599 | 14 | 73726726 | OpenSea | PAPLN | 0.063 | 0.064 | 0.064 | **6.92E-08** | **8.09E-08** | 1.13E-07 |
| cg14327296 | 14 | 1.01E+08 | N_Shelf |  | 0.039 | 0.04 | 0.04 | **8.11E-08** | **6.96E-08** | 4.70E-06 |
| cg07892295 | 15 | 98503714 | Island | ARRDC4 | -0.102 | -0.099 | -0.101 | **1.90E-09** | **8.54E-08** | 3.50E-07 |
| cg10381071 | 15 | 70391035 | Island | TLE3 | -0.09 | -0.087 | -0.088 | **7.36E-08** | 5.50E-07 | 4.67E-07 |
| cg12452298 | 15 | 67134587 | N_Shore |  | -0.098 | -0.094 | -0.09 | **2.23E-08** | 1.73E-07 | 6.92E-07 |
| cg27549079 | 15 | 64566942 | OpenSea | CTD-2116N17.1, CSNK1G1 | -0.112 | -0.106 | -0.108 | **6.34E-08** | 5.65E-07 | 4.91E-07 |
| cg00089783 | 16 | 16261857 | OpenSea | ABCC6 | 0.093 | 0.09 | 0.089 | **2.81E-08** | 1.73E-07 | 2.42E-07 |
| cg04839677 | 16 | 3048362 | OpenSea | LINC00514 | 0.043 | 0.043 | 0.042 | **3.11E-08** | **5.71E-08** | 1.17E-07 |
| cg05261851 | 16 | 68001206 | N_Shore | SLC12A4 | 0.041 | 0.039 | 0.04 | **4.67E-08** | 2.26E-07 | 1.89E-07 |
| cg23009806 | 16 | 46771757 | OpenSea | MYLK3 | 0.059 | 0.058 | 0.058 | **4.81E-08** | 9.46E-06 | 1.12E-04 |
| cg04318345 | 17 | 72400257 | OpenSea |  | 0.064 | 0.063 | 0.063 | **1.80E-08** | 6.07E-07 | 3.24E-05 |
| cg06152601 | 17 | 80444748 | OpenSea | NARF | 0.054 | 0.052 | 0.052 | **7.35E-08** | 5.38E-07 | 3.35E-07 |
| cg07104525 | 17 | 18020727 | N_Shore | MYO15A | 0.074 | 0.078 | 0.078 | **7.45E-08** | 2.77E-05 | 6.52E-05 |
| cg08691490 | 17 | 48735420 | OpenSea | ABCC3 | 0.044 | 0.043 | 0.042 | **5.32E-08** | 2.56E-07 | 5.97E-07 |
| cg08816988 | 17 | 76893060 | OpenSea | TIMP2 | 0.052 | 0.053 | 0.052 | **8.78E-08** | 9.72E-08 | 1.91E-07 |
| cg12948278 | 17 | 8987654 | OpenSea | NTN1 | 0.045 | 0.047 | 0.047 | 1.23E-07 | **7.75E-08** | **8.52E-08** |
| cg22005990 | 17 | 46659939 | Island | HOXB3, RP11-357H14.16, HOXB-AS3 | -0.109 | -0.112 | -0.11 | **7.27E-08** | 9.86E-08 | 1.87E-07 |
| cg00333824 | 18 | 63417605 | N_Shore | CDH7 | -0.106 | -0.112 | -0.111 | 9.69E-08 | **4.31E-08** | **5.61E-08** |
| cg04209573 | 19 | 42070764 | OpenSea | CEACAM21, AC005626.1 | -0.091 | -0.091 | -0.089 | **5.39E-08** | 6.90E-05 | 2.03E-04 |
| cg05007163 | 22 | 39266069 | N_Shore | CBX6 | 0.046 | 0.043 | 0.043 | **3.63E-08** | 5.25E-07 | 5.09E-07 |
| cg26646411 | 22 | 19841374 | N_Shore | GNB1L, C22orf29 | 0.078 | 0.08 | 0.078 | **4.20E-08** | **5.77E-08** | 1.30E-07 |
