# Supplementary material for: Methylation profile of individuals with sickle cell trait
Source: Epigenetics. 2025 Aug 4;20(1):2539234. doi: 10.1080/15592294.2025.2539234 (PMC12323419; doi:10.1080/15592294.2025.2539234)
Supplement: Supplemental Material [file KEPI_A_2539234_SM0609.zip › Supplementary files/SupplementaryTables.zip!/SupplementalTable3.docx]

| **DMR** | **chr** | **start** | **end** | **width** | **n CpGs** | **n DMPs** | **n DMPs +/- 1Mb around** | **Max effect** | **FDR p-value** | **Overlapping genes** |
| --- | --- | --- | --- | --- | --- | --- | --- | --- | --- | --- |
| 1 | 1 | 3566950 | 3568245 | 1296 | 21 | 0 | 0 | -0.122 | 2.92E-24 | WRAP73 |
| 2 | 1 | 32827389 | 32828191 | 803 | 11 | 0 | 0 | -0.151 | 2.59E-15 | TSSK3, FAM229A |
| 3 | 1 | 38941821 | 38942581 | 761 | 7 | 0 | 0 | -0.125 | 7.25E-12 | RP11-329N22.1, LINC01685 |
| 4 | 1 | 65729786 | 65731107 | 1322 | 10 | 0 | 2 | -0.137 | 4.02E-18 | DNAJC6 |
| 5 | 1 | 112057879 | 112058975 | 1097 | 7 | 0 | 1 | -0.128 | 2.36E-13 | ADORA3, TMIGD3 |
| 6 | 1 | 151513198 | 151513502 | 305 | 4 | 0 | 1 | -0.114 | 2.35E-11 | TUFT1, RP11-74C1.4, ENSG00000232536 |
| 7 | 1 | 186649153 | 186649877 | 725 | 12 | 1 | 1 | -0.100 | 5.52E-10 | RP5-973M2.2, PTGS2, PACERR |
| 8 | 1 | 200860002 | 200860682 | 681 | 8 | 0 | 0 | -0.195 | 1.18E-15 | C1orf106, INAVA |
| 9 | 1 | 209848306 | 209848864 | 559 | 9 | 0 | 0 | -0.151 | 7.35E-11 | G0S2, RP1-28O10.1 |
| 10 | 1 | 209979111 | 209980186 | 1076 | 14 | 0 | 0 | -0.119 | 3.92E-13 | IRF6 |
| 11 | 1 | 220921306 | 220922217 | 912 | 11 | 0 | 0 | -0.136 | 8.42E-12 | MARC2 |
| 12 | 2 | 25473895 | 25475805 | 1911 | 10 | 0 | 1 | -0.113 | 7.20E-20 | DNMT3A |
| 13 | 2 | 43019484 | 43020457 | 974 | 10 | 0 | 0 | -0.166 | 2.24E-12 | HAAO, ENSG00000288886 |
| 14 | 2 | 45159434 | 45160861 | 1428 | 16 | 2 | 2 | -0.161 | 3.46E-17 | RP11-89K21.1, LINC01833 |
| 15 | 2 | 107503547 | 107504169 | 623 | 8 | 0 | 0 | -0.107 | 9.21E-10 | ST6GAL2, ENSG00000303047 |
| 16 | 2 | 164592477 | 164592854 | 378 | 5 | 0 | 0 | -0.110 | 4.02E-10 | FIGN |
| 17 | 2 | 180725907 | 180726697 | 791 | 9 | 0 | 0 | -0.145 | 1.10E-13 | ZNF385B |
| 18 | 2 | 213403212 | 213403321 | 110 | 4 | 0 | 0 | -0.103 | 2.28E-09 | ERBB4 |
| 19 | 3 | 238931 | 239665 | 735 | 7 | 0 | 0 | -0.130 | 1.22E-13 | CHL1, CHL1-AS2 |
| 20 | 3 | 6902689 | 6903327 | 639 | 9 | 0 | 0 | -0.102 | 5.21E-11 | GRM7 |
| 21 | 3 | 24536077 | 24537469 | 1393 | 20 | 1 | 1 | -0.114 | 7.56E-14 | THRB-AS1, THRB |
| 22 | 3 | 33700962 | 33701707 | 746 | 9 | 0 | 1 | -0.106 | 3.33E-12 | CLASP2 |
| 23 | 3 | 44625992 | 44626860 | 869 | 9 | 0 | 0 | -0.129 | 9.45E-11 | RP11-944L7.5, ZNF660, ZNF197, RP11-944L7.4 |
| 24 | 3 | 54155913 | 54156332 | 420 | 4 | 0 | 0 | -0.108 | 4.52E-09 | ENSG00000286353 |
| 25 | 3 | 61547104 | 61547951 | 848 | 12 | 0 | 0 | -0.140 | 1.67E-09 | PTPRG |
| 26 | 3 | 111717534 | 111718457 | 924 | 14 | 1 | 1 | -0.105 | 4.03E-11 | TAGLN3 |
| 27 | 3 | 131080483 | 131081177 | 695 | 8 | 0 | 1 | -0.183 | 5.16E-11 | NUDT16P1, RP11-933H2.4 |
| 28 | 3 | 192127327 | 192127991 | 665 | 5 | 0 | 0 | -0.120 | 1.18E-09 | FGF12 |
| 29 | 4 | 682831 | 683376 | 546 | 9 | 0 | 0 | -0.167 | 7.18E-12 | MFSD7, SLC49A3 |
| 30 | 4 | 41362397 | 41362853 | 457 | 4 | 0 | 0 | -0.129 | 1.71E-12 | LIMCH1 |
| 31 | 4 | 74965135 | 74965278 | 144 | 5 | 0 | 0 | -0.146 | 7.35E-09 | ENSG00000289241 |
| 32 | 4 | 90757814 | 90758843 | 1030 | 11 | 0 | 0 | -0.130 | 1.59E-14 | RP11-67M1.1, SNCA-AS1 |
| 33 | 4 | 91047985 | 91049007 | 1023 | 12 | 0 | 0 | -0.111 | 1.21E-11 | CCSER1 |
| 34 | 4 | 96469519 | 96471143 | 1625 | 20 | 1 | 2 | -0.148 | 1.43E-28 | RP11-710C12.1, UNC5C-AS1 |
| 35 | 4 | 145566975 | 145567731 | 757 | 7 | 0 | 0 | -0.105 | 2.39E-11 | HHIP, HHIP-AS1 |
| 36 | 4 | 156679449 | 156681047 | 1599 | 14 | 1 | 1 | -0.151 | 5.65E-20 | GUCY1B3 |
| 37 | 4 | 157892489 | 157893306 | 818 | 7 | 0 | 0 | -0.107 | 4.63E-11 | PDGFC |
| 38 | 5 | 76115309 | 76115974 | 666 | 6 | 0 | 0 | -0.119 | 1.23E-10 | F2RL1 |
| 39 | 5 | 139076600 | 139077047 | 448 | 5 | 0 | 0 | -0.143 | 5.11E-12 | CTB-35F21.1, ENSG00000249526 |
| 40 | 5 | 150399781 | 150400531 | 751 | 11 | 0 | 0 | -0.104 | 5.14E-13 | GPX3 |
| 41 | 5 | 174151492 | 174152033 | 542 | 7 | 0 | 0 | -0.122 | 2.01E-09 | MSX2 |
| 42 | 6 | 4775638 | 4775696 | 59 | 4 | 0 | 0 | -0.150 | 2.10E-09 | CDYL-AS1, RP3-430A16.1 |
| 43 | 6 | 28601271 | 28601519 | 249 | 11 | 0 | 0 | -0.213 | 4.19E-11 | RP11-373N24.2, COX8CP1 |
| 44 | 6 | 29520698 | 29521788 | 1091 | 33 | 0 | 0 | -0.159 | 5.19E-19 | OR2I1P |
| 45 | 6 | 30043194 | 30043597 | 404 | 8 | 0 | 0 | -0.108 | 1.19E-09 | RNF39 |
| 46 | 6 | 31542556 | 31543686 | 1131 | 15 | 0 | 0 | 0.115 | 1.60E-17 | TNF |
| 47 | 6 | 33160847 | 33161148 | 302 | 10 | 0 | 0 | -0.125 | 4.99E-09 |  |
| 48 | 6 | 33180841 | 33181412 | 572 | 10 | 0 | 0 | -0.108 | 9.38E-11 |  |
| 49 | 6 | 36354232 | 36354988 | 757 | 7 | 0 | 0 | -0.100 | 5.44E-15 | RP1-50J22.4, ETV7-AS1 |
| 50 | 6 | 48036212 | 48036676 | 465 | 11 | 0 | 0 | -0.145 | 4.28E-10 | PTCHD4, ENSG00000309382 |
| 51 | 6 | 62995523 | 62996299 | 777 | 9 | 0 | 0 | -0.137 | 1.08E-12 | KHDRBS2, ENSG00000304042 |
| 52 | 6 | 123316988 | 123318023 | 1036 | 17 | 0 | 0 | -0.142 | 1.46E-19 | CLVS2 |
| 53 | 6 | 125283726 | 125284212 | 487 | 6 | 0 | 0 | -0.128 | 7.53E-13 | RNF217, RP11-510H23.1 |
| 54 | 6 | 133562035 | 133562776 | 742 | 23 | 1 | 1 | -0.111 | 4.39E-16 | EYA4 |
| 55 | 6 | 143999465 | 143999715 | 251 | 4 | 0 | 0 | -0.114 | 1.29E-10 | PHACTR2 |
| 56 | 6 | 146864278 | 146865487 | 1210 | 15 | 1 | 1 | -0.149 | 2.06E-15 | RAB32 |
| 57 | 7 | 2672400 | 2673408 | 1009 | 6 | 1 | 2 | -0.101 | 1.13E-17 | TTYH3 |
| 58 | 7 | 27195602 | 27195960 | 359 | 6 | 0 | 1 | -0.106 | 1.21E-11 | HOXA7 |
| 59 | 7 | 27204127 | 27204981 | 855 | 7 | 0 | 1 | -0.114 | 1.26E-12 | HOXA9, RP1-170O19.20, ENSG00000257184 |
| 60 | 7 | 45960806 | 45961311 | 506 | 10 | 0 | 1 | -0.130 | 2.34E-11 | IGFBP3 |
| 61 | 7 | 79764176 | 79764387 | 212 | 4 | 1 | 1 | -0.144 | 1.03E-09 | GNAI1 |
| 62 | 7 | 116962950 | 116964012 | 1063 | 10 | 1 | 2 | -0.143 | 4.36E-20 | WNT2 |
| 63 | 7 | 122525949 | 122527499 | 1551 | 19 | 0 | 0 | -0.138 | 1.89E-14 | CADPS2, ENSG00000308214 |
| 64 | 8 | 63161057 | 63161676 | 620 | 7 | 0 | 0 | -0.151 | 1.63E-11 | NKAIN3 |
| 65 | 9 | 13278977 | 13279548 | 572 | 5 | 0 | 0 | -0.161 | 1.54E-10 | MPDZ, RP11-272P10.2,ENSG00000234740 |
| 66 | 10 | 128076910 | 128077369 | 460 | 10 | 1 | 1 | -0.116 | 1.44E-12 | ADAM12, ENSG00000299806 |
| 67 | 10 | 128593718 | 128594417 | 700 | 8 | 0 | 0 | -0.142 | 3.33E-10 | DOCK1 |
| 68 | 10 | 128994030 | 128994702 | 673 | 9 | 0 | 0 | -0.137 | 9.25E-13 | DOCK1, FAM196A, INSYN2A |
| 69 | 10 | 134599149 | 134600998 | 1850 | 29 | 0 | 0 | -0.130 | 1.42E-19 | NKX6-2 |
| 70 | 11 | 2919689 | 2920819 | 1131 | 17 | 0 | 2 | 0.262 | 6.12E-26 | SLC22A18AS |
| 71 | 11 | 5617703 | 5618023 | 321 | 6 | 0 | 12 | -0.149 | 1.33E-09 | TRIM6, TRIM6-TRIM34, HBG2, AC015691.13 |
| 72 | 11 | 6340283 | 6342510 | 2228 | 19 | 6 | 15 | 0.306 | 3.84E-56 | CAVIN3, ENSG00000282556 |
| 73 | 11 | 26353484 | 26354057 | 574 | 9 | 0 | 0 | -0.144 | 1.73E-12 | ANO3-AS1 |
| 74 | 11 | 30038286 | 30038797 | 512 | 11 | 0 | 0 | -0.138 | 1.74E-10 | KCNA4, ENSG00000297137 |
| 75 | 11 | 61594708 | 61595050 | 343 | 5 | 0 | 0 | -0.104 | 1.70E-09 | FADS2, FADS1 |
| 76 | 11 | 79151188 | 79151814 | 627 | 7 | 0 | 0 | -0.108 | 7.72E-10 | TENM4 |
| 77 | 11 | 89224179 | 89224885 | 707 | 13 | 1 | 1 | -0.169 | 4.92E-11 | NOX4 |
| 78 | 11 | 111847747 | 111848164 | 418 | 5 | 0 | 0 | -0.158 | 2.38E-09 | DIXDC1 |
| 79 | 12 | 20521984 | 20522674 | 691 | 8 | 0 | 0 | -0.101 | 7.29E-10 | PDE3A, RP11-284H19.1 |
| 80 | 12 | 54070517 | 54071751 | 1235 | 16 | 2 | 3 | -0.136 | 2.63E-18 | ATP5G2, ATP5MC2 |
| 81 | 12 | 58290206 | 58290957 | 752 | 7 | 0 | 0 | -0.152 | 1.34E-10 | RP11-620J15.2, ENSG00000273805 |
| 82 | 12 | 59313817 | 59314400 | 584 | 8 | 0 | 0 | -0.150 | 1.60E-10 | LRIG3-DT |
| 83 | 12 | 63544752 | 63545288 | 537 | 8 | 0 | 0 | -0.115 | 1.33E-11 | ENSG00000279444 |
| 84 | 12 | 72665771 | 72666314 | 544 | 5 | 0 | 0 | -0.119 | 8.61E-10 | TRHDE, TRHDE-AS1 |
| 85 | 12 | 89743788 | 89744983 | 1196 | 12 | 0 | 0 | -0.100 | 1.59E-16 | DUSP6, ENSG00000274021 |
| 86 | 12 | 123752916 | 123754328 | 1413 | 7 | 0 | 0 | -0.106 | 4.36E-12 | CDK2AP1 |
| 87 | 12 | 132906376 | 132906443 | 68 | 5 | 0 | 0 | -0.122 | 2.47E-10 | RP13-895J2.7, ENSG00000255916 |
| 88 | 13 | 110438256 | 110439781 | 1526 | 10 | 1 | 2 | -0.174 | 1.19E-20 | IRS2, ENSG00000275741 |
| 89 | 14 | 24640895 | 24641706 | 812 | 12 | 0 | 0 | -0.142 | 1.52E-19 | REC8 |
| 90 | 14 | 79745664 | 79746359 | 696 | 8 | 0 | 0 | -0.101 | 7.56E-11 | NRXN3 |
| 91 | 15 | 27112049 | 27112902 | 854 | 8 | 0 | 0 | -0.156 | 5.09E-13 | GABRA5, GABRB3 |
| 92 | 15 | 58357874 | 58358384 | 511 | 13 | 0 | 0 | -0.126 | 5.04E-11 | CTD-2330J20.2, ALDH1A2-AS1 |
| 93 | 15 | 91427184 | 91428203 | 1020 | 12 | 1 | 1 | -0.131 | 3.46E-15 | FES |
| 94 | 15 | 98503376 | 98504668 | 1293 | 16 | 1 | 2 | -0.142 | 2.71E-30 | ARRDC4 |
| 95 | 16 | 6069399 | 6069731 | 333 | 5 | 0 | 0 | -0.105 | 1.12E-09 | RP11-420N3.2, RBFOX1 |
| 96 | 16 | 75589282 | 75589572 | 291 | 6 | 0 | 0 | -0.148 | 4.96E-11 | TMEM231, RP11-77K12.8, ENSG00000259992 |
| 97 | 17 | 7341436 | 7341936 | 501 | 6 | 0 | 0 | -0.124 | 1.58E-13 | RP11-104H15.7, FGF11 |
| 98 | 17 | 27044322 | 27045302 | 981 | 16 | 0 | 0 | -0.142 | 7.92E-12 | RAB34, ENSG00000295132 |
| 99 | 17 | 38501397 | 38501677 | 281 | 5 | 0 | 0 | -0.151 | 5.76E-11 | RARA |
| 100 | 17 | 43221220 | 43222121 | 902 | 7 | 2 | 3 | -0.115 | 4.53E-17 | ACBD4 |
| 101 | 17 | 46659019 | 46660097 | 1079 | 9 | 0 | 1 | -0.117 | 1.33E-18 | HOXB-AS3, HOXB3, MIR10A, ENSG00000257178 |
| 102 | 17 | 46681316 | 46681635 | 320 | 5 | 0 | 1 | 0.116 | 4.66E-11 | HOXB-AS3, HOXB3, HOXB6 |
| 103 | 18 | 5543548 | 5544237 | 690 | 12 | 0 | 0 | -0.114 | 1.83E-12 | EPB41L3 |
| 104 | 18 | 6414974 | 6415118 | 145 | 5 | 0 | 0 | -0.116 | 8.82E-09 | L3MBTL4, LINC01387 |
| 105 | 18 | 11148682 | 11149841 | 1160 | 11 | 0 | 0 | -0.104 | 7.75E-14 | PIEZO2 |
| 106 | 18 | 25757202 | 25757710 | 509 | 7 | 0 | 0 | -0.153 | 1.27E-12 | CDH2 |
| 107 | 18 | 63417605 | 63417783 | 179 | 4 | 0 | 0 | -0.106 | 2.31E-11 | CDH7 |
| 108 | 18 | 74961451 | 74962672 | 1222 | 20 | 0 | 0 | -0.134 | 1.00E-16 | GALR1 |
| 109 | 19 | 15217974 | 15218165 | 192 | 6 | 0 | 0 | -0.102 | 5.42E-09 |  |
| 110 | 19 | 42070397 | 42071240 | 844 | 9 | 2 | 4 | -0.155 | 1.62E-33 | CEACAM21 |
| 111 | 19 | 46317840 | 46319493 | 1654 | 12 | 1 | 2 | -0.161 | 3.57E-33 | RSPH6A, SYMPK |
| 112 | 19 | 56158885 | 56159710 | 826 | 5 | 0 | 0 | -0.132 | 5.62E-13 | CCDC106 |
| 113 | 19 | 57018614 | 57019373 | 760 | 10 | 1 | 1 | -0.160 | 6.44E-17 | ZNF471 |
| 114 | 19 | 57049326 | 57050834 | 1509 | 11 | 0 | 0 | -0.103 | 1.09E-15 | ZFP28, AC005498.3 |
| 115 | 20 | 13200685 | 13201225 | 541 | 15 | 0 | 0 | -0.113 | 3.92E-09 | ENSG00000296962 |
| 116 | 20 | 60641703 | 60642264 | 562 | 4 | 0 | 0 | -0.113 | 1.76E-11 | ENSG00000297946 |
| 117 | 21 | 40984285 | 40985406 | 1122 | 12 | 0 | 1 | -0.155 | 1.85E-15 | B3GALT5, C21orf88 |
| 118 | 22 | 34316316 | 34317024 | 709 | 5 | 1 | 1 | -0.131 | 2.60E-09 | LARGE |
| 119 | 22 | 46440469 | 46441127 | 659 | 7 | 0 | 0 | -0.109 | 2.51E-12 | LINC00899, MIRLET7BHG |
