# Supplementary material for: Methylation profile of individuals with sickle cell trait
Source: Epigenetics. 2025 Aug 4;20(1):2539234. doi: 10.1080/15592294.2025.2539234 (PMC12323419; doi:10.1080/15592294.2025.2539234)
Supplement: Supplemental Material [file KEPI_A_2539234_SM0609.zip › Supplementary files/SupplementaryTables.zip!/SupplementalTable4.docx]

| **cpg** | **chr** | **pos (GRCh37)** | **Top CpG in region** | **Genes** | **VISION annotation** | **UCSC Gene Location (GENCODE version 47)** | **Genehancer** | **Transcription factor binding site (ENCODE 4)** | **build38 cpG location** |
| --- | --- | --- | --- | --- | --- | --- | --- | --- | --- |
| cg06358171 | 1 | 54822008 | cg06358171 | SSBP3 | within SF_4, build 38 chr1:54356001-54357000, cCREs for Human-Mouse joint segmentation with 25 states (SF_4), candidate cis-regulatory regions (cCREs) called using IDEAS in IS mode (from https://usevision.org/trackHubs.html) | In intron SSBP3 | GH01J054353, Elite Enhancer | RFX1 and others | chr1:54356335-54356336 |
| cg19963589 | 1 | 54822103 | cg19963589 | SSBP3 | within SF_4, build 38 chr1:54356001-54357000, cCREs for Human-Mouse joint segmentation with 25 states (SF_4), candidate cis-regulatory regions (cCREs) called using IDEAS in IS mode (from https://usevision.org/trackHubs.html) | In intron SSBP3 | GH01J054353, Elite Enhancer | RFX1 and others | chr1:54356430-54356431 |
| cg23718924 | 2 | 60778617 | cg23718924 | BCL11A | within SF_1 cCRE in IDEAS, build 38 chr2:60550401-60552800, cCREs for Human-Mouse joint segmentation with 25 states (SF_1), candidate cis-regulatory regions (cCREs) called using IDEAS in IS mode (from https://usevision.org/trackHubs.html) | In intron BCL11A | GH02J060543, Elite Promoter/Enhancer | Nearby KLF16-6268- Transcription Factor Representative Peak (rPeak) Clusters (912 factors in 1152 biosamples) from ENCODE 4 (KLF16-6268), chr2:60551222-60551625, directly in ZBTB26-3275 peak | chr2:60551482-60551483 |
| cg04391064 | 7 | 43797839 | cg04391064 | BLVRA | not a cCRE from joint model | Upstream BLVRA | GH07J043757 Promoter/Enhancer, Elite, Promoter entry for BLVRA | ZBTB26 | chr7:43758240-43758241 |
| cg07570010 | 11 | 5249270 | cg27390769 | HBB | not a cCRE from joint model, is less than 1 kb from SF_2 chr11:5227001-5227400 cCREs for Human-Mouse joint segmentation with 25 states (SF_2) region | 1 kb upstream of HBB | not annotated | none | chr11:5228040-5228041 |
| cg24803687 | 11 | 5462916 | cg27390769 | OR51I1, HBG2, HBE1, AC104389.28 | not annotated | ~200 kb upstream of the fetal (HBG1 and HBG2) and HBE1 gene bodies | not annotated | none | chr11:5441686-5441687 |
| cg27390769 | 11 | 5621691 | cg27390769 | TRIM6, HBG2, AC015691.1, TRIM6-TRIM34 | In N_8 element, chr11:5600401-5600600, cCREs for Human-Mouse joint segmentation with 25 states (N_8) | Within intron of some TRIM6 transcripts | GH11J005599 Elite Enhancer, GH11J005599 targets: TRIM6, TRIM6-TRIM34, piR-34862, TRIM34, lnc-OR52H1-1, ENSG00000239920, TRIM5, validated in in K562 erythroleukemia cells using Sharpr-MPRA | CTCF | chr11:5600461-5600462 |
| Lowest scoring factors from Transcription Factor Representative Peak (rPeak) Clusters (912 factors in 1152 biosamples) from ENCODE 4 excluded | | | | | | | | | |
