# Supplementary material for: Methylation profile of individuals with sickle cell trait
Source: Epigenetics. 2025 Aug 4;20(1):2539234. doi: 10.1080/15592294.2025.2539234 (PMC12323419; doi:10.1080/15592294.2025.2539234)
Supplement: Supplemental Material [file KEPI_A_2539234_SM0609.zip › Supplementary files/SupplementaryTables.zip!/SupplementalTable5.docx]

| **Pathway** | **N** | **DE** | [**P-value**](http://p.de/) | **FDR** |
| --- | --- | --- | --- | --- |
| **DMRs** | | | | |
| GOBP_EMBRYO_DEVELOPMENT | 1128 | 35 | 1.20E-05 | 4.13E-02 |
| GOBP_ORGAN_GROWTH | 179 | 12 | 1.29E-05 | 4.13E-02 |
| GOBP_TUBE_DEVELOPMENT | 1154 | 35 | 7.94E-06 | 4.13E-02 |
| **DMPs** | | | | |
| GOBP_NEUROGENESIS | 1603 | 587 | 3.50E-12 | 3.35E-08 |
| GOBP_GENERATION_OF_NEURONS | 1380 | 514 | 2.44E-11 | 1.17E-07 |
| GOBP_CELL_CELL_SIGNALING | 1608 | 552 | 5.85E-11 | 1.40E-07 |
| GOBP_ANIMAL_ORGAN_MORPHOGENESIS | 1007 | 377 | 5.36E-11 | 1.40E-07 |
| GOBP_CELL_JUNCTION_ORGANIZATION | 698 | 283 | 7.19E-10 | 1.38E-06 |
| GOBP_ANATOMICAL_STRUCTURE_FORMATION_INVOLVED_IN_MORPHOGENESIS | 1209 | 414 | 2.55E-09 | 4.08E-06 |
| GOBP_TUBE_DEVELOPMENT | 1112 | 383 | 3.85E-09 | 5.28E-06 |
| GOBP_EMBRYONIC_MORPHOGENESIS | 600 | 234 | 6.04E-09 | 7.24E-06 |
| GOBP_CENTRAL_NERVOUS_SYSTEM_DEVELOPMENT | 1015 | 369 | 1.55E-08 | 1.36E-05 |
| GOBP_NEURON_DEVELOPMENT | 1057 | 396 | 1.51E-08 | 1.36E-05 |
| GOBP_CELL_MOTILITY | 1687 | 543 | 1.55E-08 | 1.36E-05 |
| GOBP_CIRCULATORY_SYSTEM_DEVELOPMENT | 1171 | 394 | 3.61E-08 | 2.89E-05 |
| GOBP_TUBE_MORPHOGENESIS | 909 | 315 | 4.72E-08 | 3.23E-05 |
| GOBP_REGULATION_OF_SYSTEM_PROCESS | 575 | 210 | 4.61E-08 | 3.23E-05 |
| GOBP_SYNAPTIC_SIGNALING | 744 | 274 | 1.89E-07 | 1.00E-04 |
| GOBP_TISSUE_MORPHOGENESIS | 620 | 232 | 3.47E-07 | 2.00E-04 |
| GOBP_CELL_MORPHOGENESIS | 885 | 339 | 4.20E-07 | 2.00E-04 |
| GOBP_SYNAPSE_ORGANIZATION | 431 | 176 | 4.54E-07 | 2.00E-04 |
| GOBP_AXON_DEVELOPMENT | 451 | 189 | 4.56E-07 | 2.00E-04 |
| GOBP_CELL_JUNCTION_ASSEMBLY | 420 | 170 | 6.48E-07 | 3.00E-04 |
| GOBP_CELL_ADHESION | 1456 | 475 | 9.36E-07 | 4.00E-04 |
| GOBP_ADENYLATE_CYCLASE_MODULATING_G_PROTEIN_COUPLED_RECEPTOR_SIGNALING_PATHWAY | 228 | 91 | 1.05E-06 | 5.00E-04 |
| GOBP_CELLULAR_COMPONENT_MORPHOGENESIS | 735 | 286 | 1.17E-06 | 5.00E-04 |
| GOBP_HEAD_DEVELOPMENT | 782 | 286 | 1.45E-06 | 6.00E-04 |
| GOBP_ACTIN_FILAMENT_BASED_PROCESS | 771 | 286 | 2.49E-06 | 1.00E-03 |
| GOBP_REGULATION_OF_TRANS_SYNAPTIC_SIGNALING | 467 | 183 | 2.90E-06 | 1.10E-03 |
| GOBP_SENSORY_ORGAN_DEVELOPMENT | 559 | 206 | 3.51E-06 | 1.20E-03 |
| GOBP_RENAL_SYSTEM_DEVELOPMENT | 312 | 128 | 3.55E-06 | 1.20E-03 |
| GOBP_EPITHELIUM_DEVELOPMENT | 1184 | 383 | 3.68E-06 | 1.20E-03 |
| GOBP_EMBRYONIC_ORGAN_DEVELOPMENT | 446 | 169 | 4.46E-06 | 1.40E-03 |
| GOBP_G_PROTEIN_COUPLED_RECEPTOR_SIGNALING_PATHWAY | 1113 | 276 | 8.50E-06 | 2.60E-03 |
| GOBP_CONNECTIVE_TISSUE_DEVELOPMENT | 268 | 109 | 8.60E-06 | 2.60E-03 |
| GOBP_TAXIS | 584 | 206 | 8.98E-06 | 2.60E-03 |
| GOBP_MORPHOGENESIS_OF_AN_EPITHELIUM | 505 | 190 | 1.04E-05 | 2.90E-03 |
| GOBP_REGULATION_OF_SMALL_GTPASE_MEDIATED_SIGNAL_TRANSDUCTION | 279 | 122 | 1.35E-05 | 3.70E-03 |
| KEGG_VASCULAR_SMOOTH_MUSCLE_CONTRACTION | 112 | 53 | 1.46E-05 | 3.90E-03 |
| REACTOME_SIGNALING_BY_GPCR | 670 | 210 | 1.76E-05 | 4.20E-03 |
| GOBP_VASCULATURE_DEVELOPMENT | 785 | 258 | 1.66E-05 | 4.20E-03 |
| GOBP_CIRCULATORY_SYSTEM_PROCESS | 576 | 200 | 1.74E-05 | 4.20E-03 |
| GOBP_VASCULAR_PROCESS_IN_CIRCULATORY_SYSTEM | 257 | 101 | 1.79E-05 | 4.20E-03 |
| GOBP_POSITIVE_REGULATION_OF_NERVOUS_SYSTEM_DEVELOPMENT | 262 | 111 | 1.71E-05 | 4.20E-03 |
| GOBP_EMBRYO_DEVELOPMENT | 1098 | 367 | 1.91E-05 | 4.30E-03 |
| GOBP_REGULATION_OF_SYNAPSE_ASSEMBLY | 104 | 52 | 1.92E-05 | 4.30E-03 |
| GOBP_CELL_PART_MORPHOGENESIS | 626 | 246 | 2.23E-05 | 4.90E-03 |
| GOBP_CELL_MORPHOGENESIS_INVOLVED_IN_DIFFERENTIATION | 590 | 233 | 2.51E-05 | 5.20E-03 |
| GOBP_PATTERN_SPECIFICATION_PROCESS | 452 | 165 | 2.47E-05 | 5.20E-03 |
| GOBP_EMBRYONIC_ORGAN_MORPHOGENESIS | 290 | 114 | 2.99E-05 | 6.10E-03 |
| GOBP_SYNAPSE_ASSEMBLY | 181 | 79 | 3.50E-05 | 6.70E-03 |
| GOBP_CELL_PROJECTION_ORGANIZATION | 1506 | 505 | 3.48E-05 | 6.70E-03 |
| GOBP_LOCOMOTION | 1378 | 432 | 3.42E-05 | 6.70E-03 |
| GOBP_TRANSMEMBRANE_TRANSPORT | 1485 | 448 | 3.83E-05 | 7.20E-03 |
| GOBP_SENSORY_SYSTEM_DEVELOPMENT | 387 | 147 | 4.08E-05 | 7.50E-03 |
| GOBP_TRANSMEMBRANE_RECEPTOR_PROTEIN_TYROSINE_KINASE_SIGNALING_PATHWAY | 619 | 219 | 4.60E-05 | 7.90E-03 |
| GOBP_MUSCLE_ORGAN_DEVELOPMENT | 339 | 127 | 4.58E-05 | 7.90E-03 |
| GOBP_GLIOGENESIS | 311 | 119 | 4.64E-05 | 7.90E-03 |
| GOBP_CELL_SURFACE_RECEPTOR_SIGNALING_PATHWAY_INVOLVED_IN_CELL_CELL_SIGNALING | 561 | 204 | 4.41E-05 | 7.90E-03 |
| GOBP_BLOOD_VESSEL_MORPHOGENESIS | 677 | 221 | 4.76E-05 | 8.00E-03 |
| REACTOME_MUSCLE_CONTRACTION | 197 | 82 | 5.03E-05 | 8.10E-03 |
| GOBP_REGULATION_OF_ANATOMICAL_STRUCTURE_MORPHOGENESIS | 887 | 305 | 4.97E-05 | 8.10E-03 |
| GOBP_SENSORY_ORGAN_MORPHOGENESIS | 264 | 105 | 5.05E-05 | 8.10E-03 |
| GOBP_BRANCHING_MORPHOGENESIS_OF_AN_EPITHELIAL_TUBE | 151 | 68 | 5.42E-05 | 8.50E-03 |
| GOBP_SENSORY_PERCEPTION_OF_MECHANICAL_STIMULUS | 175 | 74 | 5.52E-05 | 8.50E-03 |
| GOBP_MORPHOGENESIS_OF_A_BRANCHING_STRUCTURE | 196 | 84 | 5.64E-05 | 8.60E-03 |
| GOBP_PANCREAS_DEVELOPMENT | 80 | 39 | 5.91E-05 | 8.90E-03 |
| KEGG_DILATED_CARDIOMYOPATHY | 86 | 44 | 6.13E-05 | 8.90E-03 |
| GOBP_CELL_CELL_JUNCTION_MAINTENANCE | 18 | 14 | 6.10E-05 | 8.90E-03 |
| GOBP_GLIAL_CELL_DIFFERENTIATION | 231 | 92 | 8.47E-05 | 1.20E-02 |
| GOBP_REGULATION_OF_MEMBRANE_POTENTIAL | 415 | 150 | 8.47E-05 | 1.20E-02 |
| GOBP_MONOATOMIC_ION_TRANSPORT | 1174 | 364 | 8.93E-05 | 1.21E-02 |
| GOBP_ENZYME_LINKED_RECEPTOR_PROTEIN_SIGNALING_PATHWAY | 978 | 323 | 9.05E-05 | 1.21E-02 |
| GOBP_DORSAL_VENTRAL_PATTERN_FORMATION | 95 | 44 | 9.00E-05 | 1.21E-02 |
| GOBP_CELLULAR_RESPONSE_TO_OXYGEN_CONTAINING_COMPOUND | 1253 | 382 | 8.84E-05 | 1.21E-02 |
| GOBP_RENAL_SYSTEM_PROCESS | 118 | 50 | 1.02E-04 | 1.32E-02 |
| GOBP_REGULATION_OF_POSTSYNAPTIC_MEMBRANE_POTENTIAL | 139 | 61 | 1.01E-04 | 1.32E-02 |
| GOBP_ADENYLATE_CYCLASE_ACTIVATING_G_PROTEIN_COUPLED_RECEPTOR_SIGNALING_PATHWAY | 136 | 54 | 1.27E-04 | 1.62E-02 |
| REACTOME_NEURONAL_SYSTEM | 388 | 150 | 1.36E-04 | 1.68E-02 |
| GOBP_SMALL_GTPASE_MEDIATED_SIGNAL_TRANSDUCTION | 452 | 173 | 1.37E-04 | 1.68E-02 |
| GOBP_REGULATION_OF_LONG_TERM_NEURONAL_SYNAPTIC_PLASTICITY | 22 | 16 | 1.34E-04 | 1.68E-02 |
| KEGG_NEUROACTIVE_LIGAND_RECEPTOR_INTERACTION | 251 | 85 | 1.58E-04 | 1.90E-02 |
| REACTOME_EXTRACELLULAR_MATRIX_ORGANIZATION | 291 | 114 | 1.57E-04 | 1.90E-02 |
| GOBP_NEUROMUSCULAR_PROCESS | 153 | 63 | 1.62E-04 | 1.92E-02 |
| GOBP_ACTIN_FILAMENT_BASED_MOVEMENT | 126 | 58 | 1.65E-04 | 1.93E-02 |
| GOBP_MONOATOMIC_ION_TRANSMEMBRANE_TRANSPORT | 962 | 305 | 1.72E-04 | 1.99E-02 |
| GOBP_REGULATION_OF_SYNAPTIC_PLASTICITY | 200 | 82 | 1.77E-04 | 2.02E-02 |
| GOBP_PULMONARY_VALVE_MORPHOGENESIS | 19 | 14 | 1.80E-04 | 2.03E-02 |
| GOBP_REGULATION_OF_LOCOMOTION | 1078 | 338 | 1.85E-04 | 2.07E-02 |
| KEGG_HYPERTROPHIC_CARDIOMYOPATHY_HCM | 80 | 39 | 2.09E-04 | 2.18E-02 |
| GOBP_AMEBOIDAL_TYPE_CELL_MIGRATION | 478 | 163 | 2.11E-04 | 2.18E-02 |
| GOBP_RAS_PROTEIN_SIGNAL_TRANSDUCTION | 310 | 121 | 2.11E-04 | 2.18E-02 |
| GOBP_REGULATION_OF_RAS_PROTEIN_SIGNAL_TRANSDUCTION | 173 | 75 | 2.00E-04 | 2.18E-02 |
| GOBP_MUSCLE_ORGAN_MORPHOGENESIS | 81 | 36 | 2.16E-04 | 2.18E-02 |
| GOBP_SKELETAL_SYSTEM_MORPHOGENESIS | 224 | 90 | 2.08E-04 | 2.18E-02 |
| GOBP_UTERUS_DEVELOPMENT | 23 | 16 | 2.13E-04 | 2.18E-02 |
| GOBP_NEURON_PROJECTION_GUIDANCE | 216 | 96 | 2.14E-04 | 2.18E-02 |
| GOBP_REGULATION_OF_BLOOD_CIRCULATION | 250 | 92 | 2.16E-04 | 2.18E-02 |
| GOBP_REGULATION_OF_SYNAPSE_STRUCTURE_OR_ACTIVITY | 223 | 93 | 2.24E-04 | 2.24E-02 |
| GOBP_REGULATION_OF_CELL_JUNCTION_ASSEMBLY | 200 | 85 | 2.27E-04 | 2.24E-02 |
| GOBP_EPITHELIAL_TUBE_MORPHOGENESIS | 325 | 125 | 2.34E-04 | 2.29E-02 |
| GOBP_RESPONSE_TO_OXYGEN_CONTAINING_COMPOUND | 1740 | 505 | 2.97E-04 | 2.88E-02 |
| GOBP_SEMAPHORIN_PLEXIN_SIGNALING_PATHWAY | 41 | 25 | 3.25E-04 | 3.12E-02 |
| GOBP_CELL_JUNCTION_MAINTENANCE | 46 | 26 | 3.29E-04 | 3.13E-02 |
| GOBP_EPITHELIAL_TUBE_BRANCHING_INVOLVED_IN_LUNG_MORPHOGENESIS | 29 | 18 | 3.45E-04 | 3.25E-02 |
| GOBP_ENDOCRINE_SYSTEM_DEVELOPMENT | 135 | 57 | 3.57E-04 | 3.32E-02 |
| KEGG_CALCIUM_SIGNALING_PATHWAY | 167 | 71 | 3.69E-04 | 3.36E-02 |
| GOBP_HEART_MORPHOGENESIS | 260 | 99 | 3.72E-04 | 3.36E-02 |
| GOBP_NERVOUS_SYSTEM_PROCESS | 1382 | 365 | 3.68E-04 | 3.36E-02 |
| GOBP_REGULATION_OF_NEURON_DIFFERENTIATION | 188 | 76 | 3.91E-04 | 3.44E-02 |
| GOBP_POSITIVE_REGULATION_OF_SYNAPSE_ASSEMBLY | 56 | 30 | 3.91E-04 | 3.44E-02 |
| GOBP_CHEMICAL_SYNAPTIC_TRANSMISSION_POSTSYNAPTIC | 124 | 53 | 3.86E-04 | 3.44E-02 |
| GOBP_REGULATION_OF_NEURON_PROJECTION_DEVELOPMENT | 430 | 167 | 4.03E-04 | 3.52E-02 |
| GOBP_REGULATION_OF_NERVOUS_SYSTEM_DEVELOPMENT | 440 | 163 | 4.38E-04 | 3.79E-02 |
| REACTOME_NETRIN_MEDIATED_REPULSION_SIGNALS | 8 | 8 | 4.43E-04 | 3.80E-02 |
| REACTOME_NRAGE_SIGNALS_DEATH_THROUGH_JNK | 53 | 30 | 4.62E-04 | 3.92E-02 |
| GOBP_MUSCLE_CONTRACTION | 335 | 116 | 4.74E-04 | 3.99E-02 |
| GOBP_REGULATION_OF_VASCULAR_PERMEABILITY | 49 | 26 | 5.28E-04 | 4.36E-02 |
| GOBP_RENAL_ABSORPTION | 29 | 16 | 5.24E-04 | 4.36E-02 |
| GOBP_SYNAPTIC_TRANSMISSION_GLUTAMATERGIC | 108 | 50 | 5.49E-04 | 4.50E-02 |
| REACTOME_GPCR_LIGAND_BINDING | 441 | 117 | 5.88E-04 | 4.78E-02 |
| GOBP_AXON_ENSHEATHMENT_IN_CENTRAL_NERVOUS_SYSTEM | 24 | 15 | 5.95E-04 | 4.80E-02 |
| GOBP_REGULATION_OF_NERVOUS_SYSTEM_PROCESS | 146 | 61 | 6.06E-04 | 4.84E-02 |
| GOBP_EMBRYONIC_SKELETAL_SYSTEM_DEVELOPMENT | 127 | 55 | 6.25E-04 | 4.96E-02 |
| GOBP_EYE_MORPHOGENESIS | 155 | 65 | 6.33E-04 | 4.97E-02 |
