# Supplementary material for: Methylation profile of individuals with sickle cell trait
Source: Epigenetics. 2025 Aug 4;20(1):2539234. doi: 10.1080/15592294.2025.2539234 (PMC12323419; doi:10.1080/15592294.2025.2539234)
Supplement: Supplemental Material [file KEPI_A_2539234_SM0609.zip › Supplementary files/SupplementaryTables.zip!/SupplementalTable6.docx]

| **ID** | **Top motif proportion** | **Enrichment raw score** | **p-value** | **FDR**  **p-value** | **Motif ID** | **Gene Symbol** | **Data Source** | **TF Family** |
| --- | --- | --- | --- | --- | --- | --- | --- | --- |
| Hsapiens-jaspar2022-GRHL2-MA1105.2 | 0.068 | 6 | 1.16E-22 | 1.01E-19 | MA1105.2 | GRHL2 | jaspar2022 | Grainyhead-related factors |
| Hsapiens-jaspar2022-CTCF-MA1929.1 | 0.058 | 51 | 1.34E-16 | 5.83E-14 | MA1929.1 | CTCF | jaspar2022 | More than 3 adjacent zinc fingers |
| Hsapiens-jaspar2022-GRHL1-MA0647.1 | 0.087 | 3 | 2.90E-16 | 8.44E-14 | MA0647.1 | GRHL1 | jaspar2022 | Grainyhead-related factors |
| Hsapiens-jaspar2022-HES7-MA0822.1 | 0.029 | 2 | 2.25E-15 | 4.91E-13 | MA0822.1 | HES7 | jaspar2022 | Hairy-related factors |
| Hsapiens-jaspar2022-POU6F1-MA0628.1 | 0.087 | 3 | 1.09E-11 | 1.90E-09 | MA0628.1 | POU6F1 | jaspar2022 | POU domain factors |
| Hsapiens-jaspar2022-ERF::FOXO1-MA1936.1 | 0.107 | 9 | 1.31E-11 | 1.91E-09 | MA1936.1 | ERF::FOXO1 | jaspar2022 | Ets-related;FOX |
| Hsapiens-jaspar2022-ZNF675-MA1714.1 | 0.107 | 33 | 2.04E-11 | 2.54E-09 | MA1714.1 | ZNF675 | jaspar2022 | More than 3 adjacent zinc fingers |
| Hsapiens-jaspar2022-PKNOX2-MA0783.1 | 0.019 | 3 | 5.06E-11 | 5.52E-09 | MA0783.1 | PKNOX2 | jaspar2022 | TALE-type homeo domain factors |
| Hsapiens-jaspar2022-TGIF1-MA0796.1 | 0.019 | 1 | 1.06E-10 | 1.03E-08 | MA0796.1 | TGIF1 | jaspar2022 | TALE-type homeo domain factors |
| Hsapiens-jaspar2022-IRF3-MA1418.1 | 0.039 | 5 | 1.34E-10 | 1.17E-08 | MA1418.1 | IRF3 | jaspar2022 | Interferon-regulatory factors |
| Hsapiens-jaspar2022-ETV5::FOXI1-MA1946.1 | 0.078 | 9 | 2.68E-10 | 2.13E-08 | MA1946.1 | ETV5::FOXI1 | jaspar2022 | Ets-related;FOX |
| Hsapiens-jaspar2022-CEBPB-MA0466.3 | 0.087 | 0 | 4.75E-09 | 3.45E-07 | MA0466.3 | CEBPB | jaspar2022 | CEBP-related |
| Hsapiens-jaspar2022-TGIF2LY-MA1572.1 | 0.049 | 16 | 5.88E-09 | 3.94E-07 | MA1572.1 | TGIF2LY | jaspar2022 | TALE-type homeo domain factors |
| Hsapiens-jaspar2022-TGIF2-MA0797.1 | 0.029 | 8 | 9.99E-09 | 6.22E-07 | MA0797.1 | TGIF2 | jaspar2022 | TALE-type homeo domain factors |
| Hsapiens-jaspar2022-PLAG1-MA0163.1 | 0.117 | 2 | 1.30E-08 | 7.57E-07 | MA0163.1 | PLAG1 | jaspar2022 | More than 3 adjacent zinc fingers |
| Hsapiens-jaspar2022-ZNF708-MA1730.1 | 0.117 | 6 | 2.32E-08 | 1.27E-06 | MA1730.1 | ZNF708 | jaspar2022 | More than 3 adjacent zinc fingers |
| Hsapiens-jaspar2022-CEBPE-MA0837.2 | 0.097 | 0 | 3.80E-08 | 1.95E-06 | MA0837.2 | CEBPE | jaspar2022 | CEBP-related |
| Hsapiens-jaspar2022-VENTX-MA0724.1 | 0.087 | 1 | 6.49E-08 | 3.15E-06 | MA0724.1 | VENTX | jaspar2022 | NK |
| Hsapiens-jaspar2022-HES6-MA1493.1 | 0.087 | 1 | 8.12E-08 | 3.73E-06 | MA1493.1 | HES6 | jaspar2022 | Hairy-related factors |
| Hsapiens-jaspar2022-IKZF1-MA1508.1 | 0.155 | 12 | 1.59E-07 | 6.93E-06 | MA1508.1 | IKZF1 | jaspar2022 | Factors with multiple dispersed zinc fingers |
| Hsapiens-jaspar2022-NFE2-MA0841.1 | 0.029 | 2 | 2.18E-07 | 9.07E-06 | MA0841.1 | NFE2 | jaspar2022 | Jun-related |
| Hsapiens-jaspar2022-NFKB1-MA0105.4 | 0.068 | 0 | 1.12E-06 | 4.43E-05 | MA0105.4 | NFKB1 | jaspar2022 | NF-kappaB-related factors |
| Hsapiens-jaspar2022-MAFF-MA0495.3 | 0.029 | 7 | 1.69E-06 | 6.42E-05 | MA0495.3 | MAFF | jaspar2022 | Maf-related |
| Hsapiens-jaspar2022-TGIF2LX-MA1571.1 | 0.039 | 20 | 2.33E-06 | 8.47E-05 | MA1571.1 | TGIF2LX | jaspar2022 | TALE-type homeo domain factors |
| Hsapiens-jaspar2022-ZBTB6-MA1581.1 | 0.107 | 3 | 2.84E-06 | 9.56E-05 | MA1581.1 | ZBTB6 | jaspar2022 | More than 3 adjacent zinc fingers |
| Hsapiens-jaspar2022-TFCP2-MA1968.1 | 0.146 | 1 | 2.85E-06 | 9.56E-05 | MA1968.1 | TFCP2 | jaspar2022 | CP2-related factors |
| Hsapiens-jaspar2022-ZNF667-MA1984.1 | 0.058 | 5 | 4.34E-06 | 1.36E-04 | MA1984.1 | ZNF667 | jaspar2022 | More than 3 adjacent zinc fingers |
| Hsapiens-jaspar2022-STAT1::STAT2-MA0517.1 | 0.058 | 11 | 4.36E-06 | 1.36E-04 | MA0517.1 | STAT1::STAT2 | jaspar2022 | STAT factors;STAT factors |
| Hsapiens-jaspar2022-MSC-MA0665.1 | 0.087 | 4 | 6.67E-06 | 2.01E-04 | MA0665.1 | MSC | jaspar2022 | Tal-related |
| Hsapiens-jaspar2022-MYCN-MA0104.4 | 0.068 | 4 | 8.41E-06 | 2.44E-04 | MA0104.4 | MYCN | jaspar2022 | bHLH-ZIP |
| Hsapiens-jaspar2022-BACH2-MA1101.2 | 0.029 | 2 | 9.91E-06 | 0.00E+00 | MA1101.2 | BACH2 | jaspar2022 | Jun-related |
| Hsapiens-jaspar2022-JDP2-MA0655.1 | 0.029 | 2 | 9.98E-06 | 0.00E+00 | MA0655.1 | JDP2 | jaspar2022 | Fos-related |
| Hsapiens-jaspar2022-TCF7-MA0769.2 | 0.068 | 3 | 1.21E-05 | 0.00E+00 | MA0769.2 | TCF7 | jaspar2022 | TCF-7-related factors |
| Hsapiens-jaspar2022-ETV5::FOXO1-MA1947.1 | 0.146 | 9 | 1.24E-05 | 0.00E+00 | MA1947.1 | ETV5::FOXO1 | jaspar2022 | Ets-related;FOX |
| Hsapiens-jaspar2022-LHX6-MA0658.1 | 0.039 | 2 | 2.17E-05 | 1.00E-03 | MA0658.1 | LHX6 | jaspar2022 | HD-LIM |
| Hsapiens-jaspar2022-SIX1-MA1118.1 | 0.087 | 3 | 3.22E-05 | 1.00E-03 | MA1118.1 | SIX1 | jaspar2022 | HD-SINE |
| Hsapiens-jaspar2022-ATF2-MA1632.1 | 0.068 | 8 | 3.66E-05 | 1.00E-03 | MA1632.1 | ATF2 | jaspar2022 | Jun-related |
| Hsapiens-jaspar2022-ZNF707-MA1715.1 | 0.039 | 1 | 4.20E-05 | 1.00E-03 | MA1715.1 | ZNF707 | jaspar2022 | More than 3 adjacent zinc fingers |
| Hsapiens-jaspar2022-FOXO1::ELK3-MA1955.1 | 0.117 | 11 | 5.99E-05 | 1.00E-03 | MA1955.1 | FOXO1::ELK3 | jaspar2022 | FOX;Ets-related |
| Hsapiens-jaspar2022-ZSCAN31-MA1722.1 | 0.049 | 0 | 6.48E-05 | 1.00E-03 | MA1722.1 | ZSCAN31 | jaspar2022 | More than 3 adjacent zinc fingers |
| Hsapiens-jaspar2022-RAX-MA0718.1 | 0.107 | 2 | 6.34E-05 | 1.00E-03 | MA0718.1 | RAX | jaspar2022 | Paired-related HD factors |
| Hsapiens-jaspar2022-VSX2-MA0726.1 | 0.107 | 2 | 6.61E-05 | 1.00E-03 | MA0726.1 | VSX2 | jaspar2022 | Paired-related HD factors |
| Hsapiens-jaspar2022-MYF6-MA0667.1 | 0.078 | 2 | 7.67E-05 | 2.00E-03 | MA0667.1 | MYF6 | jaspar2022 | MyoD/ASC-related factors |
| Hsapiens-HOCOMOCOv11-core-A-CEBPB_HUMAN.H11MO.0.A | 0.078 | 1 | 8.54E-05 | 2.00E-03 | CEBPB_HUMAN.H11MO.0.A | CEBPB | HOCOMOCOv11-core-A | C/EBP-related{1.1.8}: C/EBP{1.1.8.1} |
| Hsapiens-jaspar2022-TEAD1-MA0090.3 | 0.058 | 15 | 9.23E-05 | 2.00E-03 | MA0090.3 | TEAD1 | jaspar2022 | TEF-1-related factors |
| Hsapiens-jaspar2022-SPIC-MA0687.1 | 0.049 | 8 | 1.10E-04 | 2.00E-03 | MA0687.1 | SPIC | jaspar2022 | Ets-related |
| Hsapiens-jaspar2022-ZNF449-MA1656.1 | 0.087 | 3 | 1.09E-04 | 2.00E-03 | MA1656.1 | ZNF449 | jaspar2022 | More than 3 adjacent zinc fingers |
| Hsapiens-jaspar2022-JUN-MA0488.1 | 0.049 | 9 | 1.19E-04 | 2.00E-03 | MA0488.1 | JUN | jaspar2022 | Jun-related |
| Hsapiens-jaspar2022-ZBTB32-MA1580.1 | 0.049 | 0 | 1.49E-04 | 3.00E-03 | MA1580.1 | ZBTB32 | jaspar2022 | Other factors with up to three adjacent zinc fingers |
| Hsapiens-jaspar2022-PRRX1-MA0716.1 | 0.097 | 2 | 1.62E-04 | 3.00E-03 | MA0716.1 | PRRX1 | jaspar2022 | Paired-related HD factors |
| Hsapiens-jaspar2022-POU3F3-MA0788.1 | 0.029 | 5 | 1.69E-04 | 3.00E-03 | MA0788.1 | POU3F3 | jaspar2022 | POU domain factors |
| Hsapiens-jaspar2022-MAX::MYC-MA0059.1 | 0.039 | 14 | 1.83E-04 | 3.00E-03 | MA0059.1 | MAX::MYC | jaspar2022 | bHLH-ZIP;bHLH-ZIP |
| Hsapiens-jaspar2022-UNCX-MA0721.1 | 0.068 | 2 | 1.80E-04 | 3.00E-03 | MA0721.1 | UNCX | jaspar2022 | Paired-related HD factors |
| Hsapiens-jaspar2022-VSX1-MA0725.1 | 0.087 | 2 | 1.91E-04 | 3.00E-03 | MA0725.1 | VSX1 | jaspar2022 | Paired-related HD factors |
| Hsapiens-jaspar2022-ZNF136-MA1588.1 | 0.039 | 6 | 1.97E-04 | 3.00E-03 | MA1588.1 | ZNF136 | jaspar2022 | More than 3 adjacent zinc fingers |
| Hsapiens-jaspar2022-ZBED1-MA0749.1 | 0.019 | 0 | 2.20E-04 | 3.00E-03 | MA0749.1 | ZBED1 | jaspar2022 | BED zinc finger factors |
| Hsapiens-jaspar2022-HNF1B-MA0153.2 | 0.039 | 2 | 2.21E-04 | 3.00E-03 | MA0153.2 | HNF1B | jaspar2022 | POU domain factors |
| Hsapiens-jaspar2022-LMX1B-MA0703.2 | 0.019 | 8 | 2.84E-04 | 4.00E-03 | MA0703.2 | LMX1B | jaspar2022 | HD-LIM |
| Hsapiens-jaspar2022-TP63-MA0525.2 | 0.019 | 0 | 2.86E-04 | 4.00E-03 | MA0525.2 | TP63 | jaspar2022 | p53-related factors |
| Hsapiens-jaspar2022-MZF1-MA0056.2 | 0.078 | 2 | 2.79E-04 | 4.00E-03 | MA0056.2 | MZF1 | jaspar2022 | More than 3 adjacent zinc fingers |
| Hsapiens-jaspar2022-FLI1::FOXI1-MA1950.1 | 0.078 | 9 | 2.80E-04 | 4.00E-03 | MA1950.1 | FLI1::FOXI1 | jaspar2022 | Ets-related;FOX |
| Hsapiens-jaspar2022-MLX-MA0663.1 | 0.078 | 1 | 2.85E-04 | 4.00E-03 | MA0663.1 | MLX | jaspar2022 | bHLH-ZIP |
| Hsapiens-jaspar2022-SHOX-MA0630.1 | 0.097 | 2 | 2.85E-04 | 4.00E-03 | MA0630.1 | SHOX | jaspar2022 | Paired-related HD factors |
| Hsapiens-jaspar2022-FOXO1::ELK1-MA1954.1 | 0.078 | 16 | 3.83E-04 | 5.00E-03 | MA1954.1 | FOXO1::ELK1 | jaspar2022 | FOX;Ets-related |
| Hsapiens-jaspar2022-ERF::HOXB13-MA1937.1 | 0.068 | 2 | 4.46E-04 | 6.00E-03 | MA1937.1 | ERF::HOXB13 | jaspar2022 | Ets-related;HOX-related factors |
| Hsapiens-jaspar2022-RAX2-MA0717.1 | 0.078 | 1 | 4.39E-04 | 6.00E-03 | MA0717.1 | RAX2 | jaspar2022 | Paired-related HD factors |
| Hsapiens-HOCOMOCOv11-core-A-SUH_HUMAN.H11MO.0.A | 0.117 | 4 | 4.61E-04 | 6.00E-03 | SUH_HUMAN.H11MO.0.A | SUH | HOCOMOCOv11-core-A | CSL-related factors{6.1.4}: M{6.1.4.1} |
| Hsapiens-jaspar2022-ZIM3-MA1709.1 | 0.039 | 2 | 5.67E-04 | 7.00E-03 | MA1709.1 | ZIM3 | jaspar2022 | More than 3 adjacent zinc fingers |
| Hsapiens-jaspar2022-ESX1-MA0644.2 | 0.078 | 2 | 5.82E-04 | 7.00E-03 | MA0644.2 | ESX1 | jaspar2022 | Paired-related HD factors |
| Hsapiens-jaspar2022-TFAP2A-MA0003.4 | 0.117 | 6 | 5.85E-04 | 7.00E-03 | MA0003.4 | TFAP2A | jaspar2022 | AP-2 |
| Hsapiens-jaspar2022-TLX2-MA1577.1 | 0.117 | 2 | 6.17E-04 | 8.00E-03 | MA1577.1 | TLX2 | jaspar2022 | NK |
| Hsapiens-jaspar2022-HEY2-MA0649.1 | 0.058 | 4 | 6.27E-04 | 8.00E-03 | MA0649.1 | HEY2 | jaspar2022 | Hairy-related factors |
| Hsapiens-jaspar2022-CEBPG-MA0838.1 | 0.087 | 0 | 6.72E-04 | 8.00E-03 | MA0838.1 | CEBPG | jaspar2022 | CEBP-related |
| Hsapiens-jaspar2022-ZNF684-MA1600.1 | 0.058 | 1 | 7.08E-04 | 8.00E-03 | MA1600.1 | ZNF684 | jaspar2022 | More than 3 adjacent zinc fingers |
| Hsapiens-jaspar2022-ISX-MA0654.1 | 0.078 | 2 | 7.69E-04 | 9.00E-03 | MA0654.1 | ISX | jaspar2022 | Paired-related HD factors |
| Hsapiens-jaspar2022-NOTO-MA0710.1 | 0.068 | 2 | 7.95E-04 | 9.00E-03 | MA0710.1 | NOTO | jaspar2022 | NK |
| Hsapiens-jaspar2022-RFX4-MA0799.2 | 0.078 | 0 | 8.53E-04 | 1.00E-02 | MA0799.2 | RFX4 | jaspar2022 | RFX-related factors |
| Hsapiens-HOCOMOCOv11-core-A-STAT2_HUMAN.H11MO.0.A | 0.039 | 94 | 9.32E-04 | 1.00E-02 | STAT2_HUMAN.H11MO.0.A | STAT2 | HOCOMOCOv11-core-A | STAT factors{6.2.1}: STAT2{6.2.1.0.2} |
| Hsapiens-jaspar2022-DRGX-MA1481.1 | 0.087 | 2 | 9.53E-04 | 1.10E-02 | MA1481.1 | DRGX | jaspar2022 | Paired-related HD factors |
| Hsapiens-jaspar2022-EMX2-MA0886.1 | 0.049 | 2 | 1.18E-03 | 1.30E-02 | MA0886.1 | EMX2 | jaspar2022 | NK |
| Hsapiens-jaspar2022-CEBPA-MA0102.4 | 0.126 | 1 | 1.20E-03 | 1.30E-02 | MA0102.4 | CEBPA | jaspar2022 | CEBP-related |
| Hsapiens-jaspar2022-DBP-MA0639.1 | 0.078 | 0 | 1.27E-03 | 1.30E-02 | MA0639.1 | DBP | jaspar2022 | CEBP-related |
| Hsapiens-jaspar2022-PRRX2-MA0075.3 | 0.087 | 2 | 1.31E-03 | 1.40E-02 | MA0075.3 | PRRX2 | jaspar2022 | Paired-related HD factors |
| Hsapiens-jaspar2022-EN1-MA0027.2 | 0.078 | 2 | 1.36E-03 | 1.40E-02 | MA0027.2 | EN1 | jaspar2022 | NK |
| Hsapiens-jaspar2022-LBX2-MA0699.1 | 0.068 | 1 | 1.41E-03 | 1.40E-02 | MA0699.1 | LBX2 | jaspar2022 | NK |
| Hsapiens-jaspar2022-TEAD2-MA1121.1 | 0.068 | 5 | 1.43E-03 | 1.50E-02 | MA1121.1 | TEAD2 | jaspar2022 | TEF-1-related factors |
| Hsapiens-jaspar2022-ARGFX-MA1463.1 | 0.058 | 2 | 1.48E-03 | 1.50E-02 | MA1463.1 | ARGFX | jaspar2022 | Paired-related HD factors |
| Hsapiens-jaspar2022-LHX9-MA0701.2 | 0.068 | 1 | 1.55E-03 | 1.50E-02 | MA0701.2 | LHX9 | jaspar2022 | HD-LIM |
| Hsapiens-jaspar2022-PAX1-MA0779.1 | 0.058 | 0 | 1.59E-03 | 1.60E-02 | MA0779.1 | PAX1 | jaspar2022 | Paired domain only |
| Hsapiens-jaspar2022-REST-MA0138.2 | 0.039 | 4 | 1.71E-03 | 1.70E-02 | MA0138.2 | REST | jaspar2022 | Factors with multiple dispersed zinc fingers |
| Hsapiens-jaspar2022-HESX1-MA0894.1 | 0.058 | 2 | 1.86E-03 | 1.80E-02 | MA0894.1 | HESX1 | jaspar2022 | Paired-related HD factors |
| Hsapiens-HOCOMOCOv11-core-A-MYCN_HUMAN.H11MO.0.A | 0.078 | 2 | 1.86E-03 | 1.80E-02 | MYCN_HUMAN.H11MO.0.A | MYCN | HOCOMOCOv11-core-A | bHLH-ZIP factors{1.2.6}: Myc / Max factors{1.2.6.5} |
| Hsapiens-jaspar2022-FOXO1::ELF1-MA1953.1 | 0.126 | 13 | 2.01E-03 | 1.90E-02 | MA1953.1 | FOXO1::ELF1 | jaspar2022 | FOX;Ets-related |
| Hsapiens-jaspar2022-DLX6-MA0882.1 | 0.068 | 2 | 2.04E-03 | 1.90E-02 | MA0882.1 | DLX6 | jaspar2022 | NK |
| Hsapiens-jaspar2022-HOXC8-MA1505.1 | 0.087 | 2 | 2.11E-03 | 1.90E-02 | MA1505.1 | HOXC8 | jaspar2022 | HOX |
| Hsapiens-jaspar2022-ZNF549-MA1728.1 | 0.058 | 5 | 2.26E-03 | 2.00E-02 | MA1728.1 | ZNF549 | jaspar2022 | More than 3 adjacent zinc fingers |
| Hsapiens-jaspar2022-EMX1-MA0612.2 | 0.068 | 2 | 2.27E-03 | 2.00E-02 | MA0612.2 | EMX1 | jaspar2022 | NK |
| Hsapiens-jaspar2022-MIXL1-MA0662.1 | 0.068 | 2 | 2.46E-03 | 2.20E-02 | MA0662.1 | MIXL1 | jaspar2022 | Paired-related HD factors |
| Hsapiens-jaspar2022-KLF15-MA1513.1 | 0.087 | 9 | 2.54E-03 | 2.20E-02 | MA1513.1 | KLF15 | jaspar2022 | Three-zinc finger Kruppel-related |
| Hsapiens-jaspar2022-HOXD3-MA0912.2 | 0.019 | 2 | 2.67E-03 | 2.30E-02 | MA0912.2 | HOXD3 | jaspar2022 | HOX |
| Hsapiens-jaspar2022-TEAD4-MA0809.2 | 0.058 | 7 | 2.79E-03 | 2.40E-02 | MA0809.2 | TEAD4 | jaspar2022 | TEF-1-related factors |
| Hsapiens-jaspar2022-HES5-MA0821.2 | 0.058 | 3 | 2.82E-03 | 2.40E-02 | MA0821.2 | HES5 | jaspar2022 | Hairy-related factors |
| Hsapiens-jaspar2022-VAX1-MA0722.1 | 0.039 | 2 | 2.85E-03 | 2.40E-02 | MA0722.1 | VAX1 | jaspar2022 | NK |
| Hsapiens-jaspar2022-MAX-MA0058.3 | 0.049 | 3 | 3.13E-03 | 2.60E-02 | MA0058.3 | MAX | jaspar2022 | bHLH-ZIP |
| Hsapiens-HOCOMOCOv11-core-A-REST_HUMAN.H11MO.0.A | 0.068 | 1 | 3.10E-03 | 2.60E-02 | REST_HUMAN.H11MO.0.A | REST | HOCOMOCOv11-core-A | Factors with multiple dispersed zinc fingers{2.3.4}: unclassified{2.3.4.0} |
| Hsapiens-jaspar2022-MSX1-MA0666.2 | 0.078 | 2 | 3.40E-03 | 2.80E-02 | MA0666.2 | MSX1 | jaspar2022 | NK |
| Hsapiens-jaspar2022-SOX9-MA0077.1 | 0.087 | 2 | 3.40E-03 | 2.80E-02 | MA0077.1 | SOX9 | jaspar2022 | SOX-related factors |
| Hsapiens-jaspar2022-LMX1A-MA0702.2 | 0.029 | 6 | 3.46E-03 | 2.80E-02 | MA0702.2 | LMX1A | jaspar2022 | HD-LIM |
| Hsapiens-jaspar2022-FLI1::DRGX-MA1949.1 | 0.039 | 1 | 3.76E-03 | 3.00E-02 | MA1949.1 | FLI1::DRGX | jaspar2022 | Paired-related HD factors;Ets-related |
| Hsapiens-jaspar2022-ZFP14-MA1972.1 | 0.049 | 16 | 3.78E-03 | 3.00E-02 | MA1972.1 | ZFP14 | jaspar2022 | Factors with multiple dispersed zinc fingers |
| Hsapiens-jaspar2022-ALX3-MA0634.1 | 0.087 | 2 | 3.85E-03 | 3.00E-02 | MA0634.1 | ALX3 | jaspar2022 | Paired-related HD factors |
| Hsapiens-HOCOMOCOv11-core-A-CEBPA_HUMAN.H11MO.0.A | 0.097 | 1 | 3.81E-03 | 3.00E-02 | CEBPA_HUMAN.H11MO.0.A | CEBPA | HOCOMOCOv11-core-A | C/EBP-related{1.1.8}: C/EBP{1.1.8.1} |
| Hsapiens-jaspar2022-ERF::FOXI1-MA1935.1 | 0.136 | 5 | 3.84E-03 | 3.00E-02 | MA1935.1 | ERF::FOXI1 | jaspar2022 | Ets-related;FOX |
| Hsapiens-jaspar2022-RARA::RXRA-MA0159.1 | 0.068 | 6 | 3.93E-03 | 3.00E-02 | MA0159.1 | RARA::RXRA | jaspar2022 | Thyroid hormone receptor-related factors (NR1);RXR-related receptors (NR2) |
| Hsapiens-jaspar2022-JUND-MA0492.1 | 0.029 | 8 | 4.18E-03 | 3.20E-02 | MA0492.1 | JUND | jaspar2022 | Jun-related |
| Hsapiens-jaspar2022-MSX2-MA0708.2 | 0.068 | 1 | 4.27E-03 | 3.20E-02 | MA0708.2 | MSX2 | jaspar2022 | NK |
| Hsapiens-jaspar2022-HNF1A-MA0046.2 | 0.039 | 2 | 4.36E-03 | 3.20E-02 | MA0046.2 | HNF1A | jaspar2022 | POU domain factors |
| Hsapiens-jaspar2022-DLX1-MA0879.2 | 0.068 | 1 | 4.55E-03 | 3.40E-02 | MA0879.2 | DLX1 | jaspar2022 | NK |
| Hsapiens-jaspar2022-HOXB5-MA0904.2 | 0.087 | 1 | 4.83E-03 | 3.50E-02 | MA0904.2 | HOXB5 | jaspar2022 | HOX |
| Hsapiens-jaspar2022-VAX2-MA0723.2 | 0.068 | 1 | 4.99E-03 | 3.60E-02 | MA0723.2 | VAX2 | jaspar2022 | NK |
| Hsapiens-jaspar2022-ZNF530-MA1981.1 | 0.087 | 8 | 5.33E-03 | 3.80E-02 | MA1981.1 | ZNF530 | jaspar2022 | More than 3 adjacent zinc fingers |
| Hsapiens-jaspar2022-KLF13-MA0657.1 | 0.039 | 0 | 5.50E-03 | 3.90E-02 | MA0657.1 | KLF13 | jaspar2022 | Three-zinc finger Kruppel-related |
| Hsapiens-HOCOMOCOv11-core-A-KLF15_HUMAN.H11MO.0.A | 0.078 | 77 | 5.68E-03 | 4.00E-02 | KLF15_HUMAN.H11MO.0.A | KLF15 | HOCOMOCOv11-core-A | Three-zinc finger Krüppel-related factors{2.3.1}: Krüppel-like factors{2.3.1.2} |
| Hsapiens-jaspar2022-THRA-MA1969.1 | 0.01 | 5 | 5.99E-03 | 4.20E-02 | MA1969.1 | THRA | jaspar2022 | Thyroid hormone receptor-related factors (NR1) |
| Hsapiens-HOCOMOCOv11-core-A-TEAD1_HUMAN.H11MO.0.A | 0.058 | 3 | 6.16E-03 | 4.30E-02 | TEAD1_HUMAN.H11MO.0.A | TEAD1 | HOCOMOCOv11-core-A | TEF-1-related factors{3.6.1}: TEF-1 (TEAD-1, TCF-13){3.6.1.0.1} |
| Hsapiens-jaspar2022-ETV2::FOXI1-MA1942.1 | 0.126 | 7 | 6.16E-03 | 4.30E-02 | MA1942.1 | ETV2::FOXI1 | jaspar2022 | Ets-related;FOX |
| Hsapiens-jaspar2022-MLXIPL-MA0664.1 | 0.078 | 1 | 6.28E-03 | 4.30E-02 | MA0664.1 | MLXIPL | jaspar2022 | bHLH-ZIP |
| Hsapiens-jaspar2022-GLI3-MA1491.2 | 0.029 | 0 | 6.54E-03 | 4.50E-02 | MA1491.2 | GLI3 | jaspar2022 | More than 3 adjacent zinc fingers |
| Hsapiens-jaspar2022-HOXA2-MA0900.2 | 0.019 | 2 | 6.69E-03 | 4.50E-02 | MA0900.2 | HOXA2 | jaspar2022 | HOX |
| Hsapiens-jaspar2022-ZNF417-MA1727.1 | 0.039 | 1 | 7.10E-03 | 4.80E-02 | MA1727.1 | ZNF417 | jaspar2022 | Factors with multiple dispersed zinc fingers |
