# Supplementary material for: Methylation profile of individuals with sickle cell trait
Source: Epigenetics. 2025 Aug 4;20(1):2539234. doi: 10.1080/15592294.2025.2539234 (PMC12323419; doi:10.1080/15592294.2025.2539234)
Supplement: Supplemental Material [file KEPI_A_2539234_SM0609.zip › Supplementary files/SupplementaryTables.zip!/SupplementalTable7.docx]

| **Gene** | **Gene chr** | **Gene TSS** | **CpG** | **CpG mapped genes** | **Chr** | **Position** | **Beta** | **SE** | **P-value** | **eQTM type** |
| --- | --- | --- | --- | --- | --- | --- | --- | --- | --- | --- |
| CAVIN3 | 11 | 6341877 | cg12755421 | PRKCDBP | 11 | 6342214 | -0.487 | 0.044 | 1.33E-26 | cis |
| CAVIN3 | 11 | 6341877 | cg16245261 | PRKCDBP | 11 | 6342357 | -0.365 | 0.035 | 3.58E-24 | cis |
| CAVIN3 | 11 | 6341877 | cg12479098 | PRKCDBP | 11 | 6342231 | -0.484 | 0.047 | 2.64E-23 | cis |
| CAVIN3 | 11 | 6341877 | cg03701853 | PRKCDBP | 11 | 6342337 | -0.378 | 0.037 | 2.99E-23 | cis |
| GSTM2 | 1 | 110210644 | cg03942855 | GSTM2 | 1 | 110210925 | -0.268 | 0.031 | 3.76E-17 | cis |
| PLEKHG5 | 1 | 6580121 | cg10328583 | PLEKHG5 | 1 | 6551073 | -0.710 | 0.084 | 2.03E-16 | cis |
| COA1 | 7 | 43769316 | cg04391064 | BLVRA | 7 | 43797839 | 0.086 | 0.011 | 1.20E-13 | cis |
| CSNK1G1 | 15 | 64648442 | cg27549079 | CSNK1G1; CTD-2116N17.1 | 15 | 64566942 | -0.072 | 0.010 | 1.42E-13 | cis |
| ADRB2 | 5 | 148206156 | cg15407091 |  | 5 | 148211186 | -0.248 | 0.038 | 7.60E-11 | cis |
| CCR4 | 3 | 32993066 | cg21553436 | CLASP2 | 3 | 33701149 | 0.216 | 0.035 | 1.37E-09 | cis |
| UBQLNL | 11 | 5537935 | cg27390769 | TRIM6; TRIM6-TRIM34; HBG2; AC015691.13 | 11 | 5621691 | 0.165 | 0.027 | 2.07E-09 | cis |
| TRIM34 | 11 | 5640994 | cg27390769 | TRIM6; TRIM6-TRIM34; HBG2; AC015691.13 | 11 | 5621691 | -0.040 | 0.007 | 4.51E-08 | cis |
| TTC16 | 9 | 130478345 | cg06293108 | IRS2 | 13 | 110438256 | 0.277 | 0.028 | 1.13E-21 | trans |
| CD247 | 1 | 167487847 | cg24196354 | GFOD1 | 6 | 13445503 | -0.168 | 0.018 | 4.50E-19 | trans |
| TPRG1 | 3 | 188665003 | cg06358171 | SSBP3 | 1 | 54822008 | 0.467 | 0.051 | 4.91E-19 | trans |
| MSC | 8 | 72756703 | cg09082287 | DNAJC6 | 1 | 65730494 | 0.347 | 0.038 | 9.74E-19 | trans |
| GZMH | 14 | 25078905 | cg06293108 | IRS2 | 13 | 110438256 | 0.299 | 0.033 | 1.03E-18 | trans |
| MYO6 | 6 | 76458909 | cg06358171 | SSBP3 | 1 | 54822008 | 0.411 | 0.047 | 1.25E-17 | trans |
| CCL5 | 17 | 34207797 | cg06358171 | SSBP3 | 1 | 54822008 | 0.252 | 0.029 | 2.61E-17 | trans |
| GZMH | 14 | 25078905 | cg06358171 | SSBP3 | 1 | 54822008 | 0.472 | 0.055 | 3.03E-17 | trans |
| SOX13 | 1 | 204042243 | cg06358171 | SSBP3 | 1 | 54822008 | 0.311 | 0.037 | 9.43E-17 | trans |
| CD247 | 1 | 167487847 | cg06321925 | MIR181A2HG; NR6A1 | 9 | 127420734 | -0.118 | 0.014 | 1.49E-16 | trans |
| PPP2R2B | 5 | 146464347 | cg06358171 | SSBP3 | 1 | 54822008 | 0.235 | 0.028 | 2.17E-16 | trans |
| CD8A | 2 | 87035519 | cg06293108 | IRS2 | 13 | 110438256 | 0.279 | 0.033 | 2.38E-16 | trans |
| CD8A | 2 | 87035519 | cg15984718 | UNC5C | 4 | 96470349 | 0.379 | 0.045 | 2.41E-16 | trans |
| GNAO1 | 16 | 56225302 | cg06321925 | MIR181A2HG; NR6A1 | 9 | 127420734 | 0.222 | 0.027 | 1.19E-15 | trans |
| AGFG2 | 7 | 100136834 | cg21553436 | CLASP2 | 3 | 33701149 | 0.097 | 0.012 | 1.97E-15 | trans |
| CD8A | 2 | 87035519 | cg09082287 | DNAJC6 | 1 | 65730494 | 0.278 | 0.034 | 3.10E-15 | trans |
| GNAO1 | 16 | 56225302 | cg20294789 | TUFT1; RP11-74C1.4 | 1 | 151513456 | 0.264 | 0.033 | 3.18E-15 | trans |
| TTC16 | 9 | 130478345 | cg15984718 | UNC5C | 4 | 96470349 | 0.313 | 0.039 | 3.47E-15 | trans |
| RCAN2 | 6 | 46459709 | cg06293108 | IRS2 | 13 | 110438256 | 0.613 | 0.076 | 3.54E-15 | trans |
| MSC | 8 | 72756703 | cg20294789 | TUFT1; RP11-74C1.4 | 1 | 151513456 | 0.356 | 0.044 | 4.63E-15 | trans |
| GZMH | 14 | 25078905 | cg09082287 | DNAJC6 | 1 | 65730494 | 0.274 | 0.034 | 6.06E-15 | trans |
| CD2 | 1 | 117297007 | cg09082287 | DNAJC6 | 1 | 65730494 | 0.090 | 0.011 | 7.61E-15 | trans |
| NCR1 | 19 | 55417508 | cg06321925 | MIR181A2HG; NR6A1 | 9 | 127420734 | -0.249 | 0.032 | 9.54E-15 | trans |
| GNAO1 | 16 | 56225302 | cg12783132 | PDE1B | 12 | 54943700 | 0.358 | 0.045 | 1.16E-14 | trans |
| PITPNM2 | 12 | 123634562 | cg20294789 | TUFT1; RP11-74C1.4 | 1 | 151513456 | -0.162 | 0.021 | 1.35E-14 | trans |
| IFNLR1 | 1 | 24514449 | cg15984718 | UNC5C | 4 | 96470349 | 0.315 | 0.040 | 1.42E-14 | trans |
| BANK1 | 4 | 102332443 | cg06358171 | SSBP3 | 1 | 54822008 | 0.352 | 0.045 | 1.56E-14 | trans |
| AGFG2 | 7 | 100136834 | cg24196354 | GFOD1 | 6 | 13445503 | 0.101 | 0.013 | 1.68E-14 | trans |
| CXCR5 | 11 | 118754475 | cg06358171 | SSBP3 | 1 | 54822008 | 0.343 | 0.044 | 1.78E-14 | trans |
| ERBB2 | 17 | 37844167 | cg06321925 | MIR181A2HG; NR6A1 | 9 | 127420734 | -0.211 | 0.027 | 2.22E-14 | trans |
| BLK | 8 | 11351510 | cg15984718 | UNC5C | 4 | 96470349 | 0.308 | 0.040 | 2.32E-14 | trans |
| PNOC | 8 | 28174503 | cg06358171 | SSBP3 | 1 | 54822008 | 0.357 | 0.046 | 3.94E-14 | trans |
| CD8A | 2 | 87035519 | cg07892295 | ARRDC4 | 15 | 98503714 | 0.351 | 0.046 | 4.05E-14 | trans |
| GZMH | 14 | 25078905 | cg15984718 | UNC5C | 4 | 96470349 | 0.349 | 0.045 | 4.39E-14 | trans |
| FRMPD3 | X | 106765680 | cg15984718 | UNC5C | 4 | 96470349 | 0.336 | 0.044 | 6.62E-14 | trans |
| TPRG1 | 3 | 188665003 | cg09082287 | DNAJC6 | 1 | 65730494 | 0.247 | 0.032 | 8.28E-14 | trans |
| CD79A | 19 | 42381190 | cg06358171 | SSBP3 | 1 | 54822008 | 0.394 | 0.052 | 8.67E-14 | trans |
| TRADD | 16 | 67194201 | cg21553436 | CLASP2 | 3 | 33701149 | 0.091 | 0.012 | 1.50E-13 | trans |
| BLK | 8 | 11351510 | cg06358171 | SSBP3 | 1 | 54822008 | 0.363 | 0.048 | 1.70E-13 | trans |
| SPIB | 19 | 50922195 | cg06358171 | SSBP3 | 1 | 54822008 | 0.323 | 0.043 | 2.13E-13 | trans |
| FCRLA | 1 | 161676762 | cg06358171 | SSBP3 | 1 | 54822008 | 0.436 | 0.058 | 2.45E-13 | trans |
| TPRG1 | 3 | 188665003 | cg06293108 | IRS2 | 13 | 110438256 | 0.234 | 0.031 | 2.51E-13 | trans |
| PAX5 | 9 | 37034103 | cg06358171 | SSBP3 | 1 | 54822008 | 0.403 | 0.054 | 3.48E-13 | trans |
| CD7 | 17 | 80275478 | cg21553436 | CLASP2 | 3 | 33701149 | -0.147 | 0.020 | 3.63E-13 | trans |
| THEMIS | 6 | 128239776 | cg09082287 | DNAJC6 | 1 | 65730494 | 0.139 | 0.019 | 3.68E-13 | trans |
| LAG3 | 12 | 6881678 | cg06358171 | SSBP3 | 1 | 54822008 | 0.361 | 0.049 | 3.93E-13 | trans |
| SH2D1B | 1 | 162381928 | cg09235539 | DNAJC6 | 1 | 65730481 | -0.285 | 0.039 | 4.33E-13 | trans |
| GNAO1 | 16 | 56225302 | cg09082287 | DNAJC6 | 1 | 65730494 | 0.211 | 0.029 | 4.51E-13 | trans |
| LAG3 | 12 | 6881678 | cg09082287 | DNAJC6 | 1 | 65730494 | 0.225 | 0.031 | 5.64E-13 | trans |
| MLLT3 | 9 | 20622542 | cg15407091 |  | 5 | 148211186 | 0.187 | 0.026 | 6.14E-13 | trans |
| TSPOAP1 | 17 | 56406152 | cg06321925 | MIR181A2HG; NR6A1 | 9 | 127420734 | -0.170 | 0.023 | 6.60E-13 | trans |
| THEMIS | 6 | 128239776 | cg06321925 | MIR181A2HG; NR6A1 | 9 | 127420734 | 0.131 | 0.018 | 7.71E-13 | trans |
| GNAO1 | 16 | 56225302 | cg06358171 | SSBP3 | 1 | 54822008 | 0.333 | 0.046 | 8.81E-13 | trans |
| CLIC5 | 6 | 46048132 | cg21553436 | CLASP2 | 3 | 33701149 | 0.245 | 0.034 | 1.07E-12 | trans |
| CLIC5 | 21 | 36041688 | cg21553436 | CLASP2 | 3 | 33701149 | 0.245 | 0.034 | 1.07E-12 | trans |
| AGFG2 | 7 | 100136834 | cg20294789 | TUFT1; RP11-74C1.4 | 1 | 151513456 | 0.086 | 0.012 | 1.16E-12 | trans |
| CD2 | 1 | 117297007 | cg06321925 | MIR181A2HG; NR6A1 | 9 | 127420734 | 0.079 | 0.011 | 1.18E-12 | trans |
| INPP4A | 2 | 99061317 | cg00969154 | JARID2 | 6 | 15401024 | -0.085 | 0.012 | 1.36E-12 | trans |
| NUGGC | 8 | 27941388 | cg09082287 | DNAJC6 | 1 | 65730494 | 0.267 | 0.037 | 1.39E-12 | trans |
| POU2AF1 | 11 | 111326355 | cg06358171 | SSBP3 | 1 | 54822008 | 0.317 | 0.044 | 1.44E-12 | trans |
| AGFG2 | 7 | 100136834 | cg06321925 | MIR181A2HG; NR6A1 | 9 | 127420734 | 0.071 | 0.010 | 1.46E-12 | trans |
| MANEAL | 1 | 38259474 | cg15984718 | UNC5C | 4 | 96470349 | 0.216 | 0.030 | 1.52E-12 | trans |
| SNX22 | 15 | 64443914 | cg06358171 | SSBP3 | 1 | 54822008 | 0.383 | 0.053 | 1.78E-12 | trans |
| SH2D1B | 1 | 162381928 | cg06321925 | MIR181A2HG; NR6A1 | 9 | 127420734 | -0.276 | 0.038 | 1.81E-12 | trans |
| MYO6 | 6 | 76458909 | cg09082287 | DNAJC6 | 1 | 65730494 | 0.214 | 0.030 | 1.94E-12 | trans |
