# Supplementary material for: Methylation profile of individuals with sickle cell trait
Source: Epigenetics. 2025 Aug 4;20(1):2539234. doi: 10.1080/15592294.2025.2539234 (PMC12323419; doi:10.1080/15592294.2025.2539234)
Supplement: Supplemental Material [file KEPI_A_2539234_SM0609.zip › Supplementary files/SupplementaryTables.zip!/SupplementalTable8.docx]

| **Trait** | **CpG** | **chr** | **pos** | **Beta** | **SE** | **P** | **PMID** | **Tissue** | **N** |
| --- | --- | --- | --- | --- | --- | --- | --- | --- | --- |
| Gestational age | cg10328583 | 1 | 6551073 | -2.53E-04 | 3.80E-05 | 4.12E-11 | 27717397 | Cord blood | 1068 |
| Tissue | cg10328583 | 1 | 6551073 | 1.34E-01 | 3.14E-03 | 2.60E-100 | 30602389 | Buccal cells and peripheral blood mononuclear cells | 215 |
| Age | cg10328583 | 1 | 6551073 | 1.60E-03 | 1.20E-04 | 0.00E+00 | 33450751 | Whole blood | 2338 |
| Age | cg10328583 | 1 | 6551073 | 1.30E-03 |  | 3.90E-43 | 33450751 | Whole blood | 2338 |
| C-reactive protein (CRP) levels | cg10328583 | 1 | 6551073 | 1.80E+00 | 3.43E-01 | 7.80E-08 |  | Whole blood | 17936 |
| C-reactive protein (CRP) levels | cg07247797 | 1 | 41746150 | 3.10E+00 | 4.04E-01 | 2.10E-14 |  | Whole blood | 17936 |
| C-reactive protein (CRP) levels | cg07247797 | 1 | 41746150 | 2.10E+00 | 3.95E-01 | 8.10E-08 |  | Whole blood | 14040 |
| Clear cell renal carcinoma | cg22438763 | 1 | 47900256 |  |  | 7.27E-30 | 23526956 | Clear cell renal carcinoma tumour cells, adjacent healthy cells | 129 |
| Fetal vs adult liver | cg22438763 | 1 | 47900256 | -3.51E+00 | 1.55E-01 | 2.64E-38 | 25282492 | Liver | 195 |
| Age | cg22438763 | 1 | 47900256 | 7.55E-01 | 7.68E-02 | 8.65E-23 | 25888029 | Whole blood | 143 |
| Age | cg22438763 | 1 | 47900256 |  |  | 2.88E-67 | 28056824 | Whole blood, cord blood | 269 |
| BMI | cg22438763 | 1 | 47900256 | 2.39E-01 |  | 4.90E-09 | 29278407 | Whole blood | 5361 |
| Tissue | cg22438763 | 1 | 47900256 | 2.28E-01 | 5.67E-03 | 8.30E-96 | 30602389 | Buccal cells and peripheral blood mononuclear cells | 215 |
| Age | cg22438763 | 1 | 47900256 | 3.90E-03 | 1.90E-04 | 0.00E+00 | 33450751 | Whole blood | 2338 |
| Age | cg22438763 | 1 | 47900256 | 3.30E-03 |  | 4.00E-143 | 33450751 | Whole blood | 2338 |
| Age | cg22438763 | 1 | 47900256 | -5.70E-03 | 9.20E-04 | 5.60E-10 | 33450751 | Whole blood | 2338 |
| Age | cg22438763 | 1 | 47900256 | 1.10E-02 |  | 1.80E-87 | 33450751 | Whole blood | 2338 |
| Age | cg22438763 | 1 | 47900256 | 2.20E-02 |  | 4.90E-151 | 33450751 | Whole blood | 2338 |
| schizophrenia | cg22438763 | 1 | 47900256 | -1.44E+00 | 1.91E-01 | 4.60E-14 | 33646943 | Whole blood | 3264 |
| schizophrenia | cg22438763 | 1 | 47900256 | -1.42E+00 | 2.56E-01 | 3.40E-08 | 33646943 | Whole blood | 3264 |
| C-reactive protein (CRP) levels | cg22438763 | 1 | 47900256 | 1.60E+00 | 2.42E-01 | 1.40E-10 |  | Whole blood | 17936 |
| Age | cg06358171 | 1 | 54822008 |  |  | 4.20E-80 | 28056824 | Whole blood, cord blood | 269 |
| Tissue | cg06358171 | 1 | 54822008 | -4.03E-01 | 7.28E-03 | 1.90E-120 | 30602389 | Buccal cells and peripheral blood mononuclear cells | 215 |
| Age | cg06358171 | 1 | 54822008 | 6.00E-03 | 1.90E-04 | 0.00E+00 | 33450751 | Whole blood | 2338 |
| Age | cg06358171 | 1 | 54822008 | 2.60E-03 |  | 1.80E-64 | 33450751 | Whole blood | 2338 |
| Age | cg06358171 | 1 | 54822008 | -1.10E-02 | 1.20E-03 | 0.00E+00 | 33450751 | Whole blood | 2338 |
| GZMK protein levels (SeqId = 9545-156) | cg06358171 | 1 | 54822008 | 9.80E-01 | 1.51E-01 | 8.60E-11 | 35945220 | Whole blood | 774 |
| Age | cg19963589 | 1 | 54822103 | -4.30E-04 | 7.40E-05 | 1.40E-08 | 23177740 | Whole blood | 656 |
| Tissue | cg19963589 | 1 | 54822103 | -4.31E-01 | 8.55E-03 | 5.70E-113 | 30602389 | Buccal cells and peripheral blood mononuclear cells | 215 |
| Age | cg19963589 | 1 | 54822103 | 1.10E-03 |  | 4.40E-11 | 33450751 | Whole blood | 2338 |
| Age | cg09235539 | 1 | 65730481 | 1.18E+00 | 1.30E-01 | 1.09E-19 | 25888029 | Whole blood | 143 |
| Tissue | cg09235539 | 1 | 65730481 | 5.06E-02 | 3.13E-03 | 1.10E-37 | 30602389 | Buccal cells and peripheral blood mononuclear cells | 215 |
| Age | cg09235539 | 1 | 65730481 | 1.20E-03 | 1.30E-04 | 0.00E+00 | 33450751 | Whole blood | 2338 |
| Incident COPD | cg09235539 | 1 | 65730481 | 2.90E-02 | 4.65E-03 | 7.30E-10 |  | Whole blood | 17902 |
| C-reactive protein (CRP) levels | cg09235539 | 1 | 65730481 | 2.90E+00 | 3.70E-01 | 6.30E-15 |  | Whole blood | 17936 |
| Age | cg09082287 | 1 | 65730494 | 1.11E+00 | 1.14E-01 | 1.58E-22 | 25888029 | Whole blood | 143 |
| BMI | cg09082287 | 1 | 65730494 | 3.40E-01 |  | 4.80E-11 | 29278407 | Whole blood | 5361 |
| Tissue | cg09082287 | 1 | 65730494 | 5.64E-02 | 3.89E-03 | 1.10E-32 | 30602389 | Buccal cells and peripheral blood mononuclear cells | 215 |
| Age | cg09082287 | 1 | 65730494 | 2.80E-02 | 4.70E-03 | 3.50E-09 | 31426852 | Whole Blood | 237 |
| Age | cg09082287 | 1 | 65730494 | 1.90E-03 | 9.90E-05 | 0.00E+00 | 33450751 | Whole blood | 2338 |
| Age | cg09082287 | 1 | 65730494 | 1.70E-03 |  | 1.60E-184 | 33450751 | Whole blood | 2338 |
| Age | cg09082287 | 1 | 65730494 | 4.20E-03 |  | 1.10E-10 | 33450751 | Whole blood | 2338 |
| Incident COPD | cg09082287 | 1 | 65730494 | 2.40E-02 | 4.07E-03 | 7.80E-09 |  | Whole blood | 17902 |
| C-reactive protein (CRP) levels | cg09082287 | 1 | 65730494 | 2.60E+00 | 3.00E-01 | 1.60E-17 |  | Whole blood | 17936 |
| Pancreatic ductal adenocarcinoma | cg03942855 | 1 | 110210925 |  |  | 2.97E-15 | 24500968 | Pancreatic ductal adenocarcinoma, adjacent nontransformed pancreata | 196 |
| Fetal vs adult liver | cg03942855 | 1 | 110210925 | -3.12E+00 | 1.42E-01 | 2.30E-37 | 25282492 | Liver | 195 |
| Tissue | cg03942855 | 1 | 110210925 | 1.10E-01 | 3.50E-03 | 1.60E-77 | 30602389 | Buccal cells and peripheral blood mononuclear cells | 215 |
| Age | cg03942855 | 1 | 110210925 | 2.00E-03 | 1.40E-04 | 0.00E+00 | 33450751 | Whole blood | 2338 |
| Age | cg03942855 | 1 | 110210925 | 1.50E-03 |  | 1.50E-37 | 33450751 | Whole blood | 2338 |
| Gene expression of Affymetrix ID: 2350952 (gene symbol: GSTM2) | cg03942855 | 1 | 110210925 |  |  | 3.70E-15 | 33752734 | Whole blood | 4170 |
| Gene expression of Affymetrix ID: 2350981 (gene symbol: GSTM1) | cg03942855 | 1 | 110210925 |  |  | 1.30E-22 | 33752734 | Whole blood | 4170 |
| C-reactive protein (CRP) levels | cg03942855 | 1 | 110210925 | 2.10E+00 | 3.85E-01 | 4.50E-08 |  | Whole blood | 17936 |
| Fetal vs adult liver | cg11574975 | 1 | 112058304 | -2.85E+00 | 1.09E-01 | 5.56E-43 | 25282492 | Liver | 195 |
| Age | cg11574975 | 1 | 112058304 | 6.66E-01 | 7.17E-02 | 1.48E-20 | 25888029 | Whole blood | 143 |
| BMI | cg11574975 | 1 | 112058304 | 2.95E-01 |  | 1.40E-08 | 29278407 | Whole blood | 5361 |
| Tissue | cg11574975 | 1 | 112058304 | 1.09E-01 | 3.64E-03 | 2.90E-74 | 30602389 | Buccal cells and peripheral blood mononuclear cells | 215 |
| atopy | cg11574975 | 1 | 112058304 | -4.10E-02 | 6.94E-03 | 5.00E-08 | 32795589 | nasal polyp | 108 |
| Age | cg11574975 | 1 | 112058304 | 2.30E-03 | 9.90E-05 | 0.00E+00 | 33450751 | Whole blood | 2338 |
| Age | cg11574975 | 1 | 112058304 | 2.00E-03 |  | 0.00E+00 | 33450751 | Whole blood | 2338 |
| Age | cg11574975 | 1 | 112058304 | 6.10E-03 |  | 7.90E-74 | 33450751 | Whole blood | 2338 |
| GZMK protein levels (SeqId = 9545-156) | cg11574975 | 1 | 112058304 | 6.30E-01 | 1.17E-01 | 7.30E-08 | 35945220 | Whole blood | 774 |
| C-reactive protein (CRP) levels | cg11574975 | 1 | 112058304 | 1.60E+00 | 2.16E-01 | 1.00E-13 |  | Whole blood | 17936 |
| BMI | cg20294789 | 1 | 151513456 | 2.16E-01 |  | 5.10E-09 | 29278407 | Whole blood | 5361 |
| Tissue | cg20294789 | 1 | 151513456 | 8.95E-02 | 3.05E-03 | 4.50E-73 | 30602389 | Buccal cells and peripheral blood mononuclear cells | 215 |
| atopy | cg20294789 | 1 | 151513456 | -3.20E-02 | 3.95E-03 | 1.80E-12 | 32795589 | nasal polyp | 108 |
| Age | cg20294789 | 1 | 151513456 | 2.40E-03 | 9.40E-05 | 0.00E+00 | 33450751 | Whole blood | 2338 |
| Age | cg20294789 | 1 | 151513456 | 9.80E-04 |  | 4.00E-32 | 33450751 | Whole blood | 2338 |
| C-reactive protein (CRP) levels | cg20294789 | 1 | 151513456 | 2.40E+00 | 3.97E-01 | 1.30E-09 |  | Whole blood | 17936 |
| Tissue | cg27447874 | 1 | 165346015 | 7.31E-02 | 2.39E-03 | 5.30E-76 | 30602389 | Buccal cells and peripheral blood mononuclear cells | 215 |
| Age | cg27447874 | 1 | 165346015 | 9.70E-04 | 1.10E-04 | 0.00E+00 | 33450751 | Whole blood | 2338 |
| Age | cg27447874 | 1 | 165346015 | 7.70E-04 |  | 2.30E-08 | 33450751 | Whole blood | 2338 |
| Prevalent Chronic Kidney Disease (Estimated) | cg18385689 | 1 | 226136936 | 8.90E-02 | 1.63E-02 | 5.10E-08 |  | Whole blood | 18318 |
| C-reactive protein (CRP) levels | cg18385689 | 1 | 226136936 | 3.60E+00 | 3.30E-01 | 8.80E-28 |  | Whole blood | 17936 |
| C-reactive protein (CRP) levels | cg18385689 | 1 | 226136936 | 2.50E+00 | 3.30E-01 | 6.30E-14 |  | Whole blood | 14040 |
| C-reactive protein (CRP) levels | cg19206131 | 2 | 26225806 | 4.30E+00 | 4.10E-01 | 3.00E-25 |  | Whole blood | 17936 |
| C-reactive protein (CRP) levels | cg19206131 | 2 | 26225806 | 2.60E+00 | 4.23E-01 | 1.10E-09 |  | Whole blood | 14040 |
| Tissue | cg23718924 | 2 | 60778617 | 3.98E-02 | 2.11E-03 | 1.40E-45 | 30602389 | Buccal cells and peripheral blood mononuclear cells | 215 |
| Age | cg23718924 | 2 | 60778617 | 6.20E-04 | 3.90E-05 | 0.00E+00 | 33450751 | Whole blood | 2338 |
| Age | cg23718924 | 2 | 60778617 | 7.30E-04 |  | 1.50E-242 | 33450751 | Whole blood | 2338 |
| Age | cg23718924 | 2 | 60778617 | 2.00E-03 |  | 7.30E-46 | 33450751 | Whole blood | 2338 |
| Sex | cg23718924 | 2 | 60778617 | 8.50E+00 |  | 3.20E-08 | 34044884 | Placenta | 585 |
| Tissue | cg02541582 | 2 | 64069798 | 6.93E-02 | 2.92E-03 | 3.90E-59 | 30602389 | Buccal cells and peripheral blood mononuclear cells | 215 |
| atopy | cg02541582 | 2 | 64069798 | -2.40E-02 | 4.02E-03 | 3.40E-08 | 32795589 | nasal polyp | 108 |
| Age | cg02541582 | 2 | 64069798 | 1.80E-03 | 8.00E-05 | 0.00E+00 | 33450751 | Whole blood | 2338 |
| Age | cg02541582 | 2 | 64069798 | 1.30E-03 |  | 6.10E-156 | 33450751 | Whole blood | 2338 |
| Age | cg02541582 | 2 | 64069798 | 2.70E-03 |  | 4.10E-20 | 33450751 | Whole blood | 2338 |
| Age | cg16354502 | 2 | 71697410 | -1.30E-03 | 1.10E-04 | 0.00E+00 | 33450751 | Whole blood | 2338 |
| Age | cg16354502 | 2 | 71697410 | 1.10E-03 |  | 3.70E-19 | 33450751 | Whole blood | 2338 |
| Age | cg16354502 | 2 | 71697410 | 3.10E-03 |  | 4.90E-08 | 33450751 | Whole blood | 2338 |
| C-reactive protein (CRP) levels | cg15574405 | 2 | 71776472 | -2.10E+00 | 3.74E-01 | 2.40E-08 |  | Whole blood | 17936 |
| C-reactive protein (CRP) levels | cg07347380 | 2 | 232799969 | -2.60E+00 | 3.71E-01 | 2.90E-12 |  | Whole blood | 17936 |
| Clear cell renal carcinoma | cg05287437 | 3 | 16215248 |  |  | 1.77E-42 | 23526956 | Clear cell renal carcinoma tumour cells, adjacent healthy cells | 129 |
| Gestational age | cg05287437 | 3 | 16215248 | 8.87E-02 | 1.27E-02 | 3.21E-12 | 25650246 | Fetal brain | 179 |
| Age | cg05287437 | 3 | 16215248 |  |  | 5.89E-27 | 28056824 | Whole blood, cord blood | 269 |
| Maternal body mass index | cg05287437 | 3 | 16215248 | -4.80E-04 | 7.70E-05 | 5.50E-10 | 29016858 | Cord blood | 7523 |
| Nitrogen dioxide exposure | cg05287437 | 3 | 16215248 | -4.80E-04 | 7.70E-05 | 5.50E-10 | 29410382 | Whole blood | 1656 |
| Tissue | cg05287437 | 3 | 16215248 | -9.41E-02 | 3.60E-03 | 3.30E-65 | 30602389 | Buccal cells and peripheral blood mononuclear cells | 215 |
| Age | cg05287437 | 3 | 16215248 | -3.10E-03 | 1.50E-04 | 0.00E+00 | 33450751 | Whole blood | 2338 |
| C-reactive protein (CRP) levels | cg05287437 | 3 | 16215248 | -2.30E+00 | 3.84E-01 | 2.50E-09 |  | Whole blood | 17936 |
| Incident Type 2 Diabetes | cg21553436 | 3 | 33701149 | 3.40E-02 | 5.58E-03 | 1.40E-09 |  | Whole blood | 17749 |
| C-reactive protein (CRP) levels | cg21553436 | 3 | 33701149 | 2.40E+00 | 2.85E-01 | 5.70E-17 |  | Whole blood | 17936 |
| Tissue | cg14654533 | 3 | 131080755 | 1.03E-01 | 4.38E-03 | 1.50E-58 | 30602389 | Buccal cells and peripheral blood mononuclear cells | 215 |
| Age | cg14654533 | 3 | 131080755 | 1.40E-03 | 1.60E-04 | 0.00E+00 | 33450751 | Whole blood | 2338 |
| Age | cg14654533 | 3 | 131080755 | 1.70E-03 |  | 3.10E-26 | 33450751 | Whole blood | 2338 |
| Incident COPD | cg14654533 | 3 | 131080755 | 2.00E-02 | 3.43E-03 | 9.40E-09 |  | Whole blood | 17902 |
| C-reactive protein (CRP) levels | cg14654533 | 3 | 131080755 | 1.40E+00 | 2.53E-01 | 4.70E-08 |  | Whole blood | 17936 |
| Tissue | cg11843333 | 4 | 15719138 | -4.26E-02 | 2.31E-03 | 2.00E-44 | 30602389 | Buccal cells and peripheral blood mononuclear cells | 215 |
| Age | cg11843333 | 4 | 15719138 | -9.60E-04 | 7.70E-05 | 0.00E+00 | 33450751 | Whole blood | 2338 |
| Age | cg15984718 | 4 | 96470349 | 7.56E-01 | 8.76E-02 | 5.93E-18 | 25888029 | Whole blood | 143 |
| HIV infection | cg15984718 | 4 | 96470349 | 1.17E+00 | 2.18E-01 | 7.05E-08 | 27105112 | Whole blood | 181 |
| Tissue | cg15984718 | 4 | 96470349 | 3.25E-02 | 2.57E-03 | 4.40E-27 | 30602389 | Buccal cells and peripheral blood mononuclear cells | 215 |
| Age | cg15984718 | 4 | 96470349 | 1.20E-03 | 9.00E-05 | 0.00E+00 | 33450751 | Whole blood | 2338 |
| Age | cg15984718 | 4 | 96470349 | 1.20E-03 |  | 5.00E-66 | 33450751 | Whole blood | 2338 |
| Gestational age | cg01872122 | 5 | 16940481 | -3.43E-01 | 4.97E-02 | 4.83E-12 | 25650246 | Fetal brain | 179 |
| Tissue | cg01872122 | 5 | 16940481 | -3.78E-02 | 2.60E-03 | 8.70E-33 | 30602389 | Buccal cells and peripheral blood mononuclear cells | 215 |
| Age | cg01872122 | 5 | 16940481 | -1.90E-03 | 1.50E-04 | 0.00E+00 | 33450751 | Whole blood | 2338 |
| Age | cg01872122 | 5 | 16940481 | 1.10E-03 |  | 3.70E-11 | 33450751 | Whole blood | 2338 |
| atopy | cg12193298 | 5 | 77870946 | 4.20E-02 | 4.87E-03 | 8.60E-14 | 32795589 | nasal polyp | 108 |
| Fetal vs adult liver | cg04476846 | 5 | 78791415 | 1.38E+00 | 1.03E-01 | 7.02E-23 | 25282492 | Liver | 195 |
| Tissue | cg04476846 | 5 | 78791415 | 5.84E-01 | 8.58E-03 | 8.50E-137 | 30602389 | Buccal cells and peripheral blood mononuclear cells | 215 |
| Age | cg04476846 | 5 | 78791415 | -1.90E-03 | 1.50E-04 | 0.00E+00 | 33450751 | Whole blood | 2338 |
| Age | cg04476846 | 5 | 78791415 | 1.10E-03 |  | 5.00E-10 | 33450751 | Whole blood | 2338 |
| Fetal vs adult liver | cg26898099 | 5 | 95192949 | 2.09E+00 | 8.38E-02 | 2.18E-41 | 25282492 | Liver | 195 |
| Tissue | cg26898099 | 5 | 95192949 | 1.90E-01 | 4.47E-03 | 7.70E-100 | 30602389 | Buccal cells and peripheral blood mononuclear cells | 215 |
| Perinatally acquired HIV | cg26898099 | 5 | 95192949 | 5.50E-02 |  | 6.40E-09 | 31324826 | Whole blood | 180 |
| Age | cg26898099 | 5 | 95192949 | 3.00E-03 | 2.10E-04 | 0.00E+00 | 33450751 | Whole blood | 2338 |
| Age | cg26898099 | 5 | 95192949 | -1.90E-02 | 1.30E-03 | 0.00E+00 | 33450751 | Whole blood | 2338 |
| Sex | cg26898099 | 5 | 95192949 | 1.20E-02 | 2.10E-03 | 5.60E-08 | 33450751 | Whole blood | 2338 |
| C-reactive protein (CRP) levels | cg26898099 | 5 | 95192949 | 3.70E+00 | 3.27E-01 | 4.30E-29 |  | Whole blood | 17936 |
| C-reactive protein (CRP) levels | cg26898099 | 5 | 95192949 | 2.20E+00 | 3.37E-01 | 1.30E-10 |  | Whole blood | 14040 |
| GZMK protein levels (SeqId = 9545-156) | cg24196354 | 6 | 13445503 | 8.70E-01 | 1.58E-01 | 3.20E-08 | 35945220 | Whole blood | 774 |
| C-reactive protein (CRP) levels | cg24196354 | 6 | 13445503 | 2.50E+00 | 4.53E-01 | 5.30E-08 |  | Whole blood | 17936 |
| GZMK protein levels (SeqId = 9545-156) | cg00969154 | 6 | 15401024 | 9.20E-01 | 1.50E-01 | 1.20E-09 | 35945220 | Whole blood | 774 |
| Clear cell renal carcinoma | cg22570970 | 6 | 15401067 |  |  | 2.64E-31 | 23526956 | Clear cell renal carcinoma tumour cells, adjacent healthy cells | 129 |
| Age | cg22570970 | 6 | 15401067 | 2.30E-03 | 2.10E-04 | 0.00E+00 | 33450751 | Whole blood | 2338 |
| GZMK protein levels (SeqId = 9545-156) | cg22570970 | 6 | 15401067 | 1.06E+00 | 1.47E-01 | 6.90E-13 | 35945220 | Whole blood | 774 |
| BMI | cg23694492 | 7 | 2672929 | 2.37E-01 |  | 6.20E-08 | 29278407 | Whole blood | 5361 |
| Tissue | cg23694492 | 7 | 2672929 | 1.03E-01 | 4.08E-03 | 6.10E-63 | 30602389 | Buccal cells and peripheral blood mononuclear cells | 215 |
| atopy | cg23694492 | 7 | 2672929 | -3.40E-02 | 5.55E-03 | 2.60E-08 | 32795589 | nasal polyp | 108 |
| Age | cg23694492 | 7 | 2672929 | 3.40E-03 | 1.40E-04 | 0.00E+00 | 33450751 | Whole blood | 2338 |
| Age | cg23694492 | 7 | 2672929 | 2.00E-03 |  | 6.70E-86 | 33450751 | Whole blood | 2338 |
| C-reactive protein (CRP) levels | cg23694492 | 7 | 2672929 | 1.90E+00 | 2.88E-01 | 1.20E-10 |  | Whole blood | 17936 |
| Right vs left colon adenomas | cg14573448 | 7 | 27165187 | -6.10E-01 | 9.56E-02 | 1.78E-10 | 23868178 | Colon adenomas | 135 |
| Age | cg14573448 | 7 | 27165187 | -1.16E-02 | 1.81E-03 | 7.81E-11 | 28811542 | Whole blood | 1366 |
| Tissue | cg14573448 | 7 | 27165187 | 4.14E-01 | 7.46E-03 | 1.40E-120 | 30602389 | Buccal cells and peripheral blood mononuclear cells | 215 |
| Age | cg14573448 | 7 | 27165187 | -2.90E-03 | 3.30E-04 | 0.00E+00 | 33450751 | Whole blood | 2338 |
| C-reactive protein (CRP) levels | cg14573448 | 7 | 27165187 | -1.60E+00 | 2.95E-01 | 4.70E-08 |  | Whole blood | 17936 |
| Tissue | cg04391064 | 7 | 43797839 | 1.11E-01 | 4.80E-03 | 2.60E-57 | 30602389 | Buccal cells and peripheral blood mononuclear cells | 215 |
| Age | cg04391064 | 7 | 43797839 | 1.90E-03 | 1.50E-04 | 0.00E+00 | 33450751 | Whole blood | 2338 |
| Age | cg04391064 | 7 | 43797839 | 1.90E-03 |  | 8.90E-33 | 33450751 | Whole blood | 2338 |
| Age | cg04391064 | 7 | 43797839 | 5.30E-03 | 9.00E-04 | 5.60E-09 | 33450751 | Whole blood | 2338 |
| Gene expression of Affymetrix ID: 3048134 (gene symbol: COA1) | cg04391064 | 7 | 43797839 |  |  | 1.10E-23 | 33752734 | Whole blood | 4170 |
| IL2RB protein levels (SeqId = 9343-16) | cg20586431 | 7 | 116963479 | -5.60E-01 | 1.04E-01 | 5.50E-08 | 35945220 | Whole blood | 774 |
| Incident COPD | cg20586431 | 7 | 116963479 | 3.00E-02 | 3.99E-03 | 7.40E-14 |  | Whole blood | 17902 |
| Tissue | cg00403579 | 9 | 124533992 | 1.33E-01 | 4.66E-03 | 3.30E-71 | 30602389 | Buccal cells and peripheral blood mononuclear cells | 215 |
| Age | cg00403579 | 9 | 124533992 | -1.90E-03 | 1.40E-04 | 0.00E+00 | 33450751 | Whole blood | 2338 |
| C-reactive protein (CRP) levels | cg00403579 | 9 | 124533992 | -2.90E+00 | 4.08E-01 | 8.90E-13 |  | Whole blood | 17936 |
| GZMA protein levels (SeqId = 3440-7) | cg06321925 | 9 | 127420734 | 7.30E-01 | 1.31E-01 | 2.60E-08 | 35945220 | Whole blood | 774 |
| GZMK protein levels (SeqId = 9545-156) | cg06321925 | 9 | 127420734 | 8.80E-01 | 1.31E-01 | 2.20E-11 | 35945220 | Whole blood | 774 |
| Incident COPD | cg06321925 | 9 | 127420734 | 2.50E-02 | 4.36E-03 | 9.10E-09 |  | Whole blood | 17902 |
| C-reactive protein (CRP) levels | cg06321925 | 9 | 127420734 | 2.60E+00 | 3.04E-01 | 4.40E-17 |  | Whole blood | 17936 |
| Age | cg07483709 | 10 | 29439573 |  |  | 9.17E-45 | 28056824 | Whole blood, cord blood | 269 |
| Tissue | cg07483709 | 10 | 29439573 | -2.08E-02 | 3.73E-03 | 7.50E-08 | 30602389 | Buccal cells and peripheral blood mononuclear cells | 215 |
| Age | cg07483709 | 10 | 29439573 | -3.20E-03 | 1.70E-04 | 0.00E+00 | 33450751 | Whole blood | 2338 |
| Age | cg07483709 | 10 | 29439573 | 1.20E-03 |  | 8.60E-08 | 33450751 | Whole blood | 2338 |
| Age | cg07483709 | 10 | 29439573 | 7.90E-03 | 1.10E-03 | 1.50E-12 | 33450751 | Whole blood | 2338 |
| C-reactive protein (CRP) levels | cg07483709 | 10 | 29439573 | -1.80E+00 | 3.29E-01 | 6.70E-08 |  | Whole blood | 17936 |
| Gestational age | cg15279476 | 10 | 62704447 | -3.22E-04 | 4.90E-05 | 1.01E-10 | 27717397 | Cord blood | 1068 |
| Tissue | cg15279476 | 10 | 62704447 | 1.41E-01 | 3.06E-03 | 6.70E-106 | 30602389 | Buccal cells and peripheral blood mononuclear cells | 215 |
| Age | cg15279476 | 10 | 62704447 | 1.40E-03 | 1.30E-04 | 0.00E+00 | 33450751 | Whole blood | 2338 |
| Age | cg15279476 | 10 | 62704447 | 1.10E-03 |  | 2.50E-14 | 33450751 | Whole blood | 2338 |
| Tissue | cg15334916 | 10 | 71563776 | -3.41E-02 | 2.51E-03 | 8.20E-30 | 30602389 | Buccal cells and peripheral blood mononuclear cells | 215 |
| Age | cg15334916 | 10 | 71563776 | -2.10E-03 | 1.70E-04 | 0.00E+00 | 33450751 | Whole blood | 2338 |
| Sex | cg15334916 | 10 | 71563776 | 1.80E+01 |  | 1.90E-12 | 34044884 | Placenta | 585 |
| Infant sex | cg15334916 | 10 | 71563776 |  |  | 1.10E-11 | 34569420 | Placenta | 229 |
| Maternal overweight/obesity | cg13145504 | 11 | 2594840 | -3.69E-03 | 6.70E-04 | 3.10E-08 | 29016858 | Cord blood | 7523 |
| Age | cg13145504 | 11 | 2594840 | -2.70E-03 | 1.70E-04 | 0.00E+00 | 33450751 | Whole blood | 2338 |
| Clear cell renal carcinoma | cg19250571 | 11 | 4869963 |  |  | 2.17E-40 | 23526956 | Clear cell renal carcinoma tumour cells, adjacent healthy cells | 129 |
| Tissue | cg19250571 | 11 | 4869963 | -6.69E-02 | 3.42E-03 | 1.00E-47 | 30602389 | Buccal cells and peripheral blood mononuclear cells | 215 |
| Age | cg19250571 | 11 | 4869963 | -2.70E-03 | 1.80E-04 | 0.00E+00 | 33450751 | Whole blood | 2338 |
| Age | cg19250571 | 11 | 4869963 | 1.40E-03 |  | 2.30E-09 | 33450751 | Whole blood | 2338 |
| eosinophilia | cg13507983 | 11 | 5128882 | -4.20E-02 | 6.81E-03 | 2.00E-08 | 32795589 | nasal polyp | 129 |
| Clear cell renal carcinoma | cg24803687 | 11 | 5462916 |  |  | 3.71E-34 | 23526956 | Clear cell renal carcinoma tumour cells, adjacent healthy cells | 129 |
| Age | cg24803687 | 11 | 5462916 | -2.00E-03 | 1.30E-04 | 0.00E+00 | 33450751 | Whole blood | 2338 |
| Age | cg24803687 | 11 | 5462916 | 1.50E-03 |  | 8.90E-25 | 33450751 | Whole blood | 2338 |
| Clear cell renal carcinoma | cg16776065 | 11 | 6340486 |  |  | 3.40E-18 | 23526956 | Clear cell renal carcinoma tumour cells, adjacent healthy cells | 129 |
| Fetal vs adult liver | cg16776065 | 11 | 6340486 | -2.11E+00 | 9.57E-02 | 1.97E-37 | 25282492 | Liver | 195 |
| Sex | cg16776065 | 11 | 6340486 |  |  | 3.55E-18 | 26500701 | Whole blood | 1799 |
| Age | cg16776065 | 11 | 6340486 |  |  | 2.78E-86 | 28056824 | Whole blood, cord blood | 269 |
| Tissue | cg16776065 | 11 | 6340486 | 1.35E-01 | 3.79E-03 | 5.90E-87 | 30602389 | Buccal cells and peripheral blood mononuclear cells | 215 |
| Age | cg16776065 | 11 | 6340486 | 4.50E-03 | 1.40E-04 | 0.00E+00 | 33450751 | Whole blood | 2338 |
| Age | cg16776065 | 11 | 6340486 | 2.50E-03 |  | 3.10E-163 | 33450751 | Whole blood | 2338 |
| Age | cg16776065 | 11 | 6340486 | -7.50E-03 | 7.60E-04 | 0.00E+00 | 33450751 | Whole blood | 2338 |
| Age | cg16776065 | 11 | 6340486 | 7.20E-03 |  | 1.30E-39 | 33450751 | Whole blood | 2338 |
| C-reactive protein (CRP) levels | cg16776065 | 11 | 6340486 | 1.40E+00 | 2.45E-01 | 4.50E-09 |  | Whole blood | 17936 |
| Tissue | cg04333575 | 11 | 6340890 | 7.09E-02 | 6.53E-03 | 9.40E-22 | 30602389 | Buccal cells and peripheral blood mononuclear cells | 215 |
| Age | cg04333575 | 11 | 6340890 | 1.00E-03 | 1.40E-04 | 1.60E-12 | 33450751 | Whole blood | 2338 |
| Age | cg04333575 | 11 | 6340890 | 1.20E-03 |  | 1.50E-13 | 33450751 | Whole blood | 2338 |
| Clear cell renal carcinoma | cg12755421 | 11 | 6342214 |  |  | 9.40E-26 | 23526956 | Clear cell renal carcinoma tumour cells, adjacent healthy cells | 129 |
| Pancreatic ductal adenocarcinoma | cg12755421 | 11 | 6342214 |  |  | 1.71E-30 | 24500968 | Pancreatic ductal adenocarcinoma, adjacent nontransformed pancreata | 196 |
| Fetal vs adult liver | cg12755421 | 11 | 6342214 | -1.43E+00 | 1.11E-01 | 5.56E-22 | 25282492 | Liver | 195 |
| Gestational age | cg12755421 | 11 | 6342214 | 2.20E-01 | 2.93E-02 | 5.58E-14 | 25650246 | Fetal brain | 179 |
| Sex | cg12755421 | 11 | 6342214 |  |  | 3.18E-14 | 26500701 | Whole blood | 1799 |
| Tissue | cg12755421 | 11 | 6342214 | 1.79E-01 | 6.18E-03 | 3.30E-72 | 30602389 | Buccal cells and peripheral blood mononuclear cells | 215 |
| Age | cg12755421 | 11 | 6342214 | 2.40E-03 |  | 7.80E-23 | 33450751 | Whole blood | 2338 |
| Sex | cg12755421 | 11 | 6342214 | -2.30E-02 | 2.60E-03 | 0.00E+00 | 33450751 | Whole blood | 2338 |
| Clear cell renal carcinoma | cg12479098 | 11 | 6342231 |  |  | 5.34E-45 | 23526956 | Clear cell renal carcinoma tumour cells, adjacent healthy cells | 129 |
| Gestational age | cg12479098 | 11 | 6342231 | 1.40E-01 | 2.25E-02 | 5.11E-10 | 25650246 | Fetal brain | 179 |
| Sex | cg12479098 | 11 | 6342231 |  |  | 4.58E-12 | 26500701 | Whole blood | 1799 |
| Tissue | cg12479098 | 11 | 6342231 | 9.50E-02 | 3.92E-03 | 2.30E-60 | 30602389 | Buccal cells and peripheral blood mononuclear cells | 215 |
| Age | cg12479098 | 11 | 6342231 | 1.90E-03 |  | 7.10E-27 | 33450751 | Whole blood | 2338 |
| Clear cell renal carcinoma | cg16245261 | 11 | 6342357 |  |  | 7.27E-33 | 23526956 | Clear cell renal carcinoma tumour cells, adjacent healthy cells | 129 |
| Pancreatic ductal adenocarcinoma | cg16245261 | 11 | 6342357 |  |  | 1.70E-35 | 24500968 | Pancreatic ductal adenocarcinoma, adjacent nontransformed pancreata | 196 |
| Fetal vs adult liver | cg16245261 | 11 | 6342357 | -1.79E+00 | 1.09E-01 | 2.34E-28 | 25282492 | Liver | 195 |
| Tissue | cg16245261 | 11 | 6342357 | 1.18E-01 | 6.32E-03 | 2.40E-45 | 30602389 | Buccal cells and peripheral blood mononuclear cells | 215 |
| Age | cg16245261 | 11 | 6342357 | 2.20E-03 |  | 2.20E-11 | 33450751 | Whole blood | 2338 |
| Maternal body mass index | cg17614165 | 11 | 47468663 | -4.70E-04 | 7.30E-05 | 9.00E-11 | 29016858 | Cord blood | 7523 |
| Tissue | cg17614165 | 11 | 47468663 | 1.44E-01 | 4.39E-03 | 7.80E-81 | 30602389 | Buccal cells and peripheral blood mononuclear cells | 215 |
| Age | cg17614165 | 11 | 47468663 | -1.80E-03 | 2.00E-04 | 0.00E+00 | 33450751 | Whole blood | 2338 |
| Age | cg05596552 | 11 | 64826323 | -7.09E-01 | 7.44E-02 | 1.45E-21 | 25888029 | Whole blood | 143 |
| HIV infection | cg05596552 | 11 | 64826323 | -1.40E+00 | 2.39E-01 | 4.25E-09 | 27105112 | Whole blood | 181 |
| Age | cg05596552 | 11 | 64826323 |  |  | 2.29E-24 | 28056824 | Whole blood, cord blood | 269 |
| Maternal body mass index | cg05596552 | 11 | 64826323 | -3.70E-04 | 6.60E-05 | 1.70E-08 | 29016858 | Cord blood | 7523 |
| Nitrogen dioxide exposure | cg05596552 | 11 | 64826323 | -3.70E-04 | 6.60E-05 | 1.70E-08 | 29410382 | Whole blood | 1656 |
| Tissue | cg05596552 | 11 | 64826323 | 3.32E-02 | 4.14E-03 | 9.10E-14 | 30602389 | Buccal cells and peripheral blood mononuclear cells | 215 |
| Age | cg05596552 | 11 | 64826323 | -3.90E-03 | 1.60E-04 | 0.00E+00 | 33450751 | Whole blood | 2338 |
| C-reactive protein (CRP) levels | cg05596552 | 11 | 64826323 | -2.00E+00 | 3.71E-01 | 7.00E-08 |  | Whole blood | 17936 |
| Age | cg07117751 | 11 | 76811860 | -1.90E-03 | 1.60E-04 | 0.00E+00 | 33450751 | Whole blood | 2338 |
| atopy | cg12783132 | 12 | 54943700 | -2.90E-02 | 4.82E-03 | 4.50E-08 | 32795589 | nasal polyp | 108 |
| Age | cg06293108 | 13 | 110438256 | 6.97E-01 | 8.62E-02 | 6.38E-16 | 25888029 | Whole blood | 143 |
| Tissue | cg06293108 | 13 | 110438256 | 5.89E-02 | 4.14E-03 | 7.50E-32 | 30602389 | Buccal cells and peripheral blood mononuclear cells | 215 |
| Age | cg06293108 | 13 | 110438256 | 1.60E-03 | 1.00E-04 | 0.00E+00 | 33450751 | Whole blood | 2338 |
| Age | cg06293108 | 13 | 110438256 | 1.70E-03 |  | 3.20E-146 | 33450751 | Whole blood | 2338 |
| Sex | cg06293108 | 13 | 110438256 | -6.00E-03 | 8.40E-04 | 1.20E-12 | 33450751 | Whole blood | 2338 |
| Incident COPD | cg06293108 | 13 | 110438256 | 1.90E-02 | 3.18E-03 | 3.30E-09 |  | Whole blood | 17902 |
| C-reactive protein (CRP) levels | cg06293108 | 13 | 110438256 | 1.40E+00 | 2.47E-01 | 1.10E-08 |  | Whole blood | 17936 |
| C-reactive protein (CRP) levels | cg08675225 | 14 | 58606095 | 1.90E+00 | 2.03E-01 | 1.70E-20 |  | Whole blood | 17936 |
| Age | cg14327296 | 14 | 101051064 |  |  | 2.99E-49 | 28056824 | Whole blood, cord blood | 269 |
| Tissue | cg14327296 | 14 | 101051064 | 2.60E-01 | 5.36E-03 | 3.30E-110 | 30602389 | Buccal cells and peripheral blood mononuclear cells | 215 |
| Smoking | cg14327296 | 14 | 101051064 | -4.27E-03 | 7.50E-04 | 1.00E-08 | 31536415 | Whole blood, CD4+ T cells, CD14+ monocytes | 15907 |
| Age | cg14327296 | 14 | 101051064 | -1.80E-03 | 1.60E-04 | 0.00E+00 | 33450751 | Whole blood | 2338 |
| GZMK protein levels (SeqId = 9545-156) | cg27549079 | 15 | 64566942 | 9.80E-01 | 1.74E-01 | 1.50E-08 | 35945220 | Whole blood | 774 |
| C-reactive protein (CRP) levels | cg27549079 | 15 | 64566942 | 2.60E+00 | 3.72E-01 | 3.10E-12 |  | Whole blood | 17936 |
| Tissue | cg12452298 | 15 | 67134587 | 1.92E-01 | 3.65E-03 | 1.60E-116 | 30602389 | Buccal cells and peripheral blood mononuclear cells | 215 |
| Age | cg12452298 | 15 | 67134587 | 4.10E-03 | 2.10E-04 | 0.00E+00 | 33450751 | Whole blood | 2338 |
| Age | cg12452298 | 15 | 67134587 | 2.50E-03 |  | 1.30E-54 | 33450751 | Whole blood | 2338 |
| Age | cg12452298 | 15 | 67134587 | 6.20E-03 |  | 7.50E-10 | 33450751 | Whole blood | 2338 |
| C-reactive protein (CRP) levels | cg12452298 | 15 | 67134587 | 1.70E+00 | 2.91E-01 | 4.60E-09 |  | Whole blood | 17936 |
| Sex | cg10381071 | 15 | 70391035 |  |  | 6.35E-10 | 26500701 | Whole blood | 1799 |
| Smoking | cg10381071 | 15 | 70391035 | -6.10E-03 | 1.10E-03 | 1.50E-08 | 27651444 | CD4+ T cells, monocytes | 9389 |
| Age | cg10381071 | 15 | 70391035 |  |  | 5.39E-33 | 28056824 | Whole blood, cord blood | 269 |
| Tissue | cg10381071 | 15 | 70391035 | 7.60E-02 | 4.28E-03 | 1.80E-42 | 30602389 | Buccal cells and peripheral blood mononuclear cells | 215 |
| body mass index | cg10381071 | 15 | 70391035 |  |  | 4.00E-11 | 31510868 | Whole blood | 1686 |
| Smoking | cg10381071 | 15 | 70391035 | -6.25E-03 | 8.60E-04 | 3.10E-13 | 31536415 | Whole blood, CD4+ T cells, CD14+ monocytes | 15907 |
| atopy | cg10381071 | 15 | 70391035 | -2.90E-02 | 3.37E-03 | 1.20E-13 | 32795589 | nasal polyp | 108 |
| Age | cg10381071 | 15 | 70391035 | 4.60E-03 | 1.50E-04 | 0.00E+00 | 33450751 | Whole blood | 2338 |
| Age | cg10381071 | 15 | 70391035 | 1.80E-03 |  | 1.40E-44 | 33450751 | Whole blood | 2338 |
| Incident Type 2 Diabetes | cg10381071 | 15 | 70391035 | -3.20E-02 | 5.33E-03 | 3.20E-09 |  | Whole blood | 17749 |
| Prevalent Type 2 Diabetes (Self-report) | cg10381071 | 15 | 70391035 | -6.60E-02 | 1.19E-02 | 3.20E-08 |  | Whole blood | 18005 |
| C-reactive protein (CRP) levels | cg10381071 | 15 | 70391035 | -2.80E+00 | 2.78E-01 | 3.10E-23 |  | Whole blood | 17936 |
| Age | cg07892295 | 15 | 98503714 | 5.17E-01 | 9.14E-02 | 1.57E-08 | 25888029 | Whole blood | 143 |
| Tissue | cg07892295 | 15 | 98503714 | 2.61E-02 | 2.49E-03 | 1.20E-20 | 30602389 | Buccal cells and peripheral blood mononuclear cells | 215 |
| Age | cg07892295 | 15 | 98503714 | 8.40E-04 | 7.50E-05 | 0.00E+00 | 33450751 | Whole blood | 2338 |
| Age | cg07892295 | 15 | 98503714 | 7.20E-04 |  | 5.90E-23 | 33450751 | Whole blood | 2338 |
| Sex | cg04839677 | 16 | 3048362 |  |  | 6.98E-08 | 26500701 | Whole blood | 1799 |
| Maternal body mass index | cg04839677 | 16 | 3048362 | -4.30E-04 | 7.10E-05 | 1.10E-09 | 29016858 | Cord blood | 7523 |
| Nitrogen dioxide exposure | cg04839677 | 16 | 3048362 | -4.30E-04 | 7.10E-05 | 1.10E-09 | 29410382 | Whole blood | 1656 |
| Tissue | cg04839677 | 16 | 3048362 | -6.02E-02 | 2.85E-03 | 4.00E-52 | 30602389 | Buccal cells and peripheral blood mononuclear cells | 215 |
| Age | cg04839677 | 16 | 3048362 | -1.10E-03 | 1.50E-04 | 5.50E-13 | 33450751 | Whole blood | 2338 |
| Age | cg05261851 | 16 | 68001206 |  |  | 1.57E-91 | 28056824 | Whole blood, cord blood | 269 |
| Tissue | cg05261851 | 16 | 68001206 | 2.56E-01 | 5.04E-03 | 1.30E-113 | 30602389 | Buccal cells and peripheral blood mononuclear cells | 215 |
| Age | cg05261851 | 16 | 68001206 | -4.20E-03 | 1.50E-04 | 0.00E+00 | 33450751 | Whole blood | 2338 |
| Age | cg05261851 | 16 | 68001206 | 1.10E-02 | 8.90E-04 | 0.00E+00 | 33450751 | Whole blood | 2338 |
| PRG3 protein levels (SeqId = 9015-1) | cg05261851 | 16 | 68001206 | -1.59E+00 | 2.90E-01 | 4.00E-08 | 35945220 | Whole blood | 774 |
| Clear cell renal carcinoma | cg10758466 | 17 | 41974087 |  |  | 6.06E-38 | 23526956 | Clear cell renal carcinoma tumour cells, adjacent healthy cells | 129 |
| Tissue | cg10758466 | 17 | 41974087 | -7.66E-02 | 3.62E-03 | 3.00E-52 | 30602389 | Buccal cells and peripheral blood mononuclear cells | 215 |
| Age | cg10758466 | 17 | 41974087 | -3.40E-03 | 1.60E-04 | 0.00E+00 | 33450751 | Whole blood | 2338 |
| Clear cell renal carcinoma | cg27395066 | 17 | 43221220 |  |  | 1.18E-43 | 23526956 | Clear cell renal carcinoma tumour cells, adjacent healthy cells | 129 |
| Gestational age | cg27395066 | 17 | 43221220 | 1.37E-01 | 2.30E-02 | 2.58E-09 | 25650246 | Fetal brain | 179 |
| Tissue | cg27395066 | 17 | 43221220 | 2.81E-01 | 7.80E-03 | 1.30E-87 | 30602389 | Buccal cells and peripheral blood mononuclear cells | 215 |
| atopy | cg27395066 | 17 | 43221220 | -4.20E-02 | 6.98E-03 | 2.40E-08 | 32795589 | nasal polyp | 108 |
| Age | cg27395066 | 17 | 43221220 | 5.10E-03 | 2.90E-04 | 0.00E+00 | 33450751 | Whole blood | 2338 |
| Age | cg27395066 | 17 | 43221220 | 2.50E-03 |  | 9.80E-09 | 33450751 | Whole blood | 2338 |
| Sex | cg27395066 | 17 | 43221220 | -2.80E-02 | 3.70E-03 | 1.90E-14 | 33450751 | Whole blood | 2338 |
| Clear cell renal carcinoma | cg22005990 | 17 | 46659939 |  |  | 4.80E-22 | 23526956 | Clear cell renal carcinoma tumour cells, adjacent healthy cells | 129 |
| Tissue | cg22005990 | 17 | 46659939 | 1.29E-01 | 3.95E-03 | 1.10E-80 | 30602389 | Buccal cells and peripheral blood mononuclear cells | 215 |
| Age | cg22005990 | 17 | 46659939 | 8.30E-04 | 1.20E-04 | 1.10E-12 | 33450751 | Whole blood | 2338 |
| Age | cg22005990 | 17 | 46659939 | 1.80E-03 |  | 1.60E-137 | 33450751 | Whole blood | 2338 |
| Age | cg22005990 | 17 | 46659939 | 4.40E-03 |  | 6.90E-32 | 33450751 | Whole blood | 2338 |
| Infant sex | cg22005990 | 17 | 46659939 |  |  | 7.00E-13 | 34569420 | Umbilical artery | 229 |
| Age | cg08816988 | 17 | 76893060 |  |  | 6.51E-35 | 28056824 | Whole blood, cord blood | 269 |
| Maternal body mass index | cg08816988 | 17 | 76893060 | -4.30E-04 | 7.90E-05 | 3.70E-08 | 29016858 | Cord blood | 7523 |
| Nitrogen dioxide exposure | cg08816988 | 17 | 76893060 | -4.30E-04 | 7.90E-05 | 3.70E-08 | 29410382 | Whole blood | 1656 |
| Tissue | cg08816988 | 17 | 76893060 | -2.78E-02 | 3.85E-03 | 1.10E-11 | 30602389 | Buccal cells and peripheral blood mononuclear cells | 215 |
| Age | cg08816988 | 17 | 76893060 | -3.70E-03 | 1.80E-04 | 0.00E+00 | 33450751 | Whole blood | 2338 |
| C-reactive protein (CRP) levels | cg06459984 | 17 | 79381472 | 3.10E+00 | 3.97E-01 | 3.40E-15 |  | Whole blood | 17936 |
| C-reactive protein (CRP) levels | cg06459984 | 17 | 79381472 | 3.30E+00 | 4.16E-01 | 2.70E-15 |  | Whole blood | 14040 |
| Gestational age | cg13957558 | 19 | 11435095 | -5.56E-01 | 6.79E-02 | 2.55E-16 | 25650246 | Fetal brain | 179 |
| Tissue | cg13957558 | 19 | 11435095 | 4.69E-02 | 1.90E-03 | 1.10E-61 | 30602389 | Buccal cells and peripheral blood mononuclear cells | 215 |
| Age | cg13957558 | 19 | 11435095 | 2.00E-04 | 2.70E-05 | 2.50E-13 | 33450751 | Whole blood | 2338 |
| Age | cg13957558 | 19 | 11435095 | 3.70E-04 |  | 1.00E-88 | 33450751 | Whole blood | 2338 |
| Age | cg13957558 | 19 | 11435095 | 8.90E-04 |  | 3.30E-08 | 33450751 | Whole blood | 2338 |
| Clear cell renal carcinoma | cg17810176 | 19 | 36035831 |  |  | 2.34E-28 | 23526956 | Clear cell renal carcinoma tumour cells, adjacent healthy cells | 129 |
| Fetal vs adult liver | cg17810176 | 19 | 36035831 | -1.68E+00 | 1.26E-01 | 9.03E-23 | 25282492 | Liver | 195 |
| Tissue | cg17810176 | 19 | 36035831 | 1.34E-01 | 4.24E-03 | 2.30E-78 | 30602389 | Buccal cells and peripheral blood mononuclear cells | 215 |
| Age | cg17810176 | 19 | 36035831 | 2.40E-03 | 1.30E-04 | 0.00E+00 | 33450751 | Whole blood | 2338 |
| Age | cg17810176 | 19 | 36035831 | 2.10E-03 |  | 1.70E-129 | 33450751 | Whole blood | 2338 |
| Age | cg17810176 | 19 | 36035831 | 4.90E-03 |  | 2.10E-16 | 33450751 | Whole blood | 2338 |
| atopy | cg01265982 | 19 | 42070875 | -1.80E-02 | 3.06E-03 | 2.90E-08 | 32795589 | nasal polyp | 108 |
| Fetal vs adult liver | cg17929770 | 19 | 46318514 | -2.39E+00 | 1.02E-01 | 2.38E-39 | 25282492 | Liver | 195 |
| Age | cg17929770 | 19 | 46318514 | 6.04E-01 | 6.57E-02 | 3.82E-20 | 25888029 | Whole blood | 143 |
| HIV infection | cg17929770 | 19 | 46318514 | 1.50E+00 | 2.02E-01 | 9.57E-14 | 27105112 | Whole blood | 181 |
| Age | cg17929770 | 19 | 46318514 |  |  | 6.34E-14 | 28056824 | Whole blood, cord blood | 269 |
| Tissue | cg17929770 | 19 | 46318514 | -4.05E-01 | 7.28E-03 | 7.30E-121 | 30602389 | Buccal cells and peripheral blood mononuclear cells | 215 |
| Smoking | cg17929770 | 19 | 46318514 | 6.38E-03 | 1.04E-03 | 7.70E-10 | 31536415 | Whole blood, CD4+ T cells, CD14+ monocytes | 15907 |
| Age | cg17929770 | 19 | 46318514 | 2.20E-03 | 1.70E-04 | 0.00E+00 | 33450751 | Whole blood | 2338 |
| Age | cg17929770 | 19 | 46318514 | 2.50E-03 |  | 2.60E-104 | 33450751 | Whole blood | 2338 |
| Age | cg17929770 | 19 | 46318514 | 4.40E-03 |  | 3.80E-11 | 33450751 | Whole blood | 2338 |
| Downs Syndrome | cg17929770 | 19 | 46318514 | 7.97E-02 | 1.23E-02 | 8.80E-11 | 33547282 | Whole Blood | 635 |
| Downs Syndrome | cg17929770 | 19 | 46318514 | 8.00E-02 | 1.30E-02 | 6.70E-10 | 33547282 | Whole Blood | 635 |
| Downs Syndrome | cg17929770 | 19 | 46318514 | 1.02E-01 | 1.80E-02 | 1.40E-08 | 33547282 | Whole Blood | 357 |
| GZMK protein levels (SeqId = 9545-156) | cg17929770 | 19 | 46318514 | 7.00E-01 | 1.17E-01 | 2.00E-09 | 35945220 | Whole blood | 774 |
| Incident COPD | cg17929770 | 19 | 46318514 | 2.90E-02 | 3.81E-03 | 1.70E-14 |  | Whole blood | 17902 |
| C-reactive protein (CRP) levels | cg17929770 | 19 | 46318514 | 1.20E+00 | 1.94E-01 | 1.20E-09 |  | Whole blood | 17936 |
| Age | cg15407257 | 20 | 23621136 |  |  | 6.51E-45 | 28056824 | Whole blood, cord blood | 269 |
| Maternal body mass index | cg15407257 | 20 | 23621136 | -5.40E-04 | 8.50E-05 | 2.00E-10 | 29016858 | Cord blood | 7523 |
| Maternal overweight/obesity | cg15407257 | 20 | 23621136 | -4.21E-03 | 7.70E-04 | 4.10E-08 | 29016858 | Cord blood | 7523 |
| Tissue | cg15407257 | 20 | 23621136 | 4.88E-02 | 5.70E-03 | 3.60E-15 | 30602389 | Buccal cells and peripheral blood mononuclear cells | 215 |
| Age | cg15407257 | 20 | 23621136 | -3.00E-03 | 1.60E-04 | 0.00E+00 | 33450751 | Whole blood | 2338 |
| Clear cell renal carcinoma | cg23057648 | 21 | 40984763 |  |  | 4.58E-21 | 23526956 | Clear cell renal carcinoma tumour cells, adjacent healthy cells | 129 |
| Tissue | cg23057648 | 21 | 40984763 | 3.50E-02 | 3.06E-03 | 1.80E-23 | 30602389 | Buccal cells and peripheral blood mononuclear cells | 215 |
| Smoking | cg23057648 | 21 | 40984763 | 1.20E-01 | 2.13E-02 | 1.70E-08 | 31552803 | Whole blood | 1851 |
| Age | cg23057648 | 21 | 40984763 | 5.20E-04 | 6.60E-05 | 3.10E-15 | 33450751 | Whole blood | 2338 |
| Age | cg23057648 | 21 | 40984763 | 1.10E-03 |  | 1.20E-148 | 33450751 | Whole blood | 2338 |
| Age | cg23057648 | 21 | 40984763 | 2.60E-03 |  | 8.40E-32 | 33450751 | Whole blood | 2338 |
| Incident COPD | cg23057648 | 21 | 40984763 | 3.00E-02 | 4.37E-03 | 8.00E-12 |  | Whole blood | 17902 |
| Fetal vs adult liver | cg26646411 | 22 | 19841374 | 1.50E+00 | 9.78E-02 | 1.58E-26 | 25282492 | Liver | 195 |
| Sex | cg26646411 | 22 | 19841374 |  |  | 2.36E-12 | 26500701 | Whole blood | 1799 |
| Tissue | cg26646411 | 22 | 19841374 | 4.47E-01 | 7.68E-03 | 3.20E-124 | 30602389 | Buccal cells and peripheral blood mononuclear cells | 215 |
| Age | cg26646411 | 22 | 19841374 | -4.50E-03 | 2.10E-04 | 0.00E+00 | 33450751 | Whole blood | 2338 |
| Age | cg26646411 | 22 | 19841374 | 9.30E-03 | 1.40E-03 | 6.60E-11 | 33450751 | Whole blood | 2338 |
| Sex | cg26646411 | 22 | 19841374 | 1.20E-02 | 2.00E-03 | 1.90E-09 | 33450751 | Whole blood | 2338 |
| Incident COPD | cg26646411 | 22 | 19841374 | -3.90E-02 | 6.04E-03 | 1.90E-10 |  | Whole blood | 17902 |
| C-reactive protein (CRP) levels | cg26646411 | 22 | 19841374 | -1.30E+00 | 2.15E-01 | 5.80E-10 |  | Whole blood | 17936 |
| Clear cell renal carcinoma | cg05007163 | 22 | 39266069 |  |  | 7.34E-33 | 23526956 | Clear cell renal carcinoma tumour cells, adjacent healthy cells | 129 |
| Maternal body mass index | cg05007163 | 22 | 39266069 | -4.50E-04 | 7.10E-05 | 1.70E-10 | 29016858 | Cord blood | 7523 |
| Maternal overweight/obesity | cg05007163 | 22 | 39266069 | -3.74E-03 | 6.90E-04 | 6.60E-08 | 29016858 | Cord blood | 7523 |
| Tissue | cg05007163 | 22 | 39266069 | 3.56E-02 | 3.99E-03 | 4.00E-16 | 30602389 | Buccal cells and peripheral blood mononuclear cells | 215 |
| Age | cg05007163 | 22 | 39266069 | -5.30E-03 | 1.80E-04 | 0.00E+00 | 33450751 | Whole blood | 2338 |
| Age | cg05007163 | 22 | 39266069 | 7.40E-03 | 1.30E-03 | 1.90E-08 | 33450751 | Whole blood | 2338 |
| Incident COPD | cg05007163 | 22 | 39266069 | -6.80E-02 | 1.09E-02 | 3.30E-10 |  | Whole blood | 17902 |
| Incident Type 2 Diabetes | cg05007163 | 22 | 39266069 | -7.00E-02 | 1.29E-02 | 4.70E-08 |  | Whole blood | 17749 |
| C-reactive protein (CRP) levels | cg05007163 | 22 | 39266069 | -4.40E+00 | 4.14E-01 | 9.70E-27 |  | Whole blood | 17936 |
