# Supplementary material for: Methylation profile of individuals with sickle cell trait
Source: Epigenetics. 2025 Aug 4;20(1):2539234. doi: 10.1080/15592294.2025.2539234 (PMC12323419; doi:10.1080/15592294.2025.2539234)
Supplement: Supplemental Material [file KEPI_A_2539234_SM0609.zip › Supplementary files/SupplementaryTables.zip!/SupplementalTable9.docx]

| **cpg** | **chr** | **Position CpG (GRCh37)** | **SNP rsID** | **Position SNP (GRCh37)** | **Mapped Gene** | **Trait Association** |
| --- | --- | --- | --- | --- | --- | --- |
| cg23718924 | 2 | 60778617 | rs1397318 | 60788481 | BCL11A - RN7SL361P | Mean corpuscular hemoglobin |
| cg23718924 | 2 | 60778617 | rs2665668 | 60768978 | BCL11A | Red blood cell count |
| cg04391064 | 7 | 43797839 | rs1181602 | 43795677 | COA1 - BLVRA | Mean corpuscular volume |
| cg19250571 | 11 | 4869963 | rs77554180 | 4872287 | MMP26 | Red blood cell count |
| cg07570010 | 11 | 5249270 | rs11549407 | 5248004 | HBB | Red blood cell count; Hemoglobin levels; Red cell distribution width; Hemoglobin; Hematocrit; Mean sphered cell volume; Mean corpuscular hemoglobin concentration |
| cg07570010 | 11 | 5249270 | rs334 | 5248232 | HBB | Mean corpuscular volume; Red cell distribution width; hemolysis of donated blood (osmotic); Hematocrit; Mean corpuscular hemoglobin concentration |
| cg07570010 | 11 | 5249270 | rs33930165 | 5248233 | HBB | Red blood cell count; Mean corpuscular volume; Mean corpuscular hemoglobin concentration; Red cell distribution width; Mean corpuscular hemoglobin |
| cg07570010 | 11 | 5249270 | rs33946267 | 5246908 | HBB | Mean corpuscular volume |
| cg07570010 | 11 | 5249270 | rs34029390 | 5246732 | HBB | Mean corpuscular hemoglobin |
| cg07570010 | 11 | 5249270 | rs35152987 | 5255582 | HBD | Hemoglobin levels |
| cg07570010 | 11 | 5249270 | rs4402323 | 5258592 | HBD | Red cell distribution width |
| cg07570010 | 11 | 5249270 | rs4910735 | 5258852 | HBD | Red cell distribution width |
| cg24803687 | 11 | 5462916 | rs1809863 | 5471541 | HBE1, OR51B5, HBG2, OR51I2 | Red blood cell count |
| cg24803687 | 11 | 5462916 | rs551956132 | 5464316 | OR51B5, HBE1, HBG2 | Mean corpuscular hemoglobin; Mean corpuscular volume; Red blood cell count |
| cg24803687 | 11 | 5462916 | rs7948471 | 5471746 | OR51B5, OR51I2, HBG2, HBE1 | Sickle cell anemia (haemolysis) |
| cg22694445 | 11 | 5570731 | rs763972480 | 5565898 | OR52H1 | Mean corpuscular hemoglobin; Mean corpuscular volume |
| cg22694445 | 11 | 5570731 | rs11038031 | 5563548 | HBG2 | Hematocrit; Red blood cell count; Hemoglobin |
| cg17614165 | 11 | 47468663 | rs3740685 | 47468791 | RAPSN | Mean corpuscular hemoglobin; Red cell distribution width |
| cg17614165 | 11 | 47468663 | rs3824867 | 47468569 | RAPSN | Mean corpuscular hemoglobin |
| cg10381071 | 15 | 70391035 | rs12441129 | 70387560 | TLE3 | Red blood cell count |
| cg00089783 | 16 | 16261857 | rs41278174 | 16259596 | ABCC6 | Red blood cell count; Hematocrit; Hemoglobin concentration; Hemoglobin levels |
| cg06459984 | 17 | 79381472 | rs34386017 | 79373962 | BAHCC1 | Red blood cell count |
| cg17929770 | 19 | 46318514 | rs16980027 | 46313105 | RSPH6A | Red blood cell count |
| cg17929770 | 19 | 46318514 | rs139063750 | 46312068 | RSPH6A | Mean corpuscular volume |
| cg17929770 | 19 | 46318514 | rs74450753 | 46317709 | RSPH6A | Mean corpuscular volume |
