# Supplementary material for: Methylation profile of individuals with sickle cell trait
Source: Epigenetics. 2025 Aug 4;20(1):2539234. doi: 10.1080/15592294.2025.2539234 (PMC12323419; doi:10.1080/15592294.2025.2539234)
Supplement: Supplemental Material [file KEPI_A_2539234_SM0609.zip › Supplementary files/SupplementaryTables.zip!/SupplementalTable10.docx]

|  | **Chronological Age Correlation** | **Sample average (SD)** | | **Unadjusted** | | **Adjusted no PC** | | **Adjusted** | | **Adjusted + SES** | |
| --- | --- | --- | --- | --- | --- | --- | --- | --- | --- | --- | --- |
|  |  | **SCT** | **No SCT** | **Estimate (CI)** | **pvalue** | **Estimate (CI)** | **pvalue** | **Estimate (CI)** | **pvalue** | **Estimate (CI)** | **pvalue** |
| **WHI** | | | | | | | | | | | |
| **Horvath2** | 0.824 | 58.85 (5.92) | 58.44 (5.92) | 0.41 (-0.11, 0.94) | 0.125 | 0.02 (-0.27, 0.31) | 0.893 | 0.07 (-0.24, 0.38) | 0.664 | 0.11 (-0.22, 0.44) | 0.508 |
| **PC Horvath2** | 0.634 | 57.1 (6.25) | 57.66 (6.25) | -0.56 (-1.12, -0.00) | **0.048** | -0.58 (-0.99, -0.17) | **0.006** | -0.37 (-0.81, 0.07) | 0.096 | -0.43 (-0.89, 0.03) | 0.068 |
| **Hannum** | 0.716 | 44.44 (6.59) | 44.4 (6.59) | 0.04 (-0.56, 0.64) | 0.892 | -0.53 (-0.92, -0.14) | **0.008** | -0.37 (-0.79, 0.05) | 0.085 | -0.35 (-0.79, 0.09) | 0.116 |
| **PC Hannum** | 0.704 | 61.14 (6.53) | 61.23 (6.53) | -0.10 (-0.68, 0.48) | 0.747 | -0.42 (-0.80, -0.04) | **0.030** | -0.19 (-0.60, 0.21) | 0.346 | -0.21 (-0.64, 0.21) | 0.326 |
| **PhenoAge** | 0.653 | 51.15 (7.89) | 49.99 (7.89) | 1.15 (0.42, 1.88) | **0.002** | 0.50 (-0.01, 1.02) | 0.056 | 0.54 (-0.01, 1.09) | 0.055 | 0.36 (-0.21, 0.93) | 0.211 |
| **PC PhenoAge** | 0.693 | 58.14 (7.78) | 57.09 (7.78) | 1.05 (0.35, 1.76) | **0.003** | 0.57 (0.19, 0.96) | **0.003** | 0.56 (0.16, 0.97) | **0.007** | 0.43 (0.01, 0.85) | **0.047** |
| **GrimAge PC** | 0.876 | 72.18 (5.9) | 71.59 (5.9) | 0.59 (0.05, 1.13) | **0.032** | 0.32 (0.09, 0.55) | **0.006** | 0.37 (0.13, 0.62) | **0.003** | 0.29 (0.04, 0.54) | **0.025** |
| **DunedinPACE** | 0.122 | 1.01 (0.11) | 0.99 (0.11) | 0.02 (0.01, 0.03) | **0.000** | 0.02 (0.01, 0.03) | **0.000** | 0.02 (0.01, 0.03) | **0.001** | 0.01 (0.00, 0.02) | **0.020** |
| **Horvath2 residuals** | - | 0.06 (3.37) | -0.06 (3.37) | 0.12 (-0.18, 0.42) | 0.431 | 0.03 (-0.26, 0.32) | 0.858 | 0.07 (-0.24, 0.38) | 0.665 | 0.12 (-0.21, 0.45) | 0.472 |
| **PC Horvath2 residuals** | - | -0.42 (4.89) | 0.38 (4.89) | -0.80 (-1.23, -0.37) | **0.000** | -0.57 (-0.98, -0.17) | **0.006** | -0.37 (-0.81, 0.07) | 0.096 | -0.43 (-0.89, 0.04) | 0.071 |
| **Hannum residuals** | - | -0.13 (4.63) | 0.12 (4.63) | -0.25 (-0.66, 0.17) | 0.246 | -0.52 (-0.91, -0.13) | **0.010** | -0.37 (-0.79, 0.05) | 0.086 | -0.33 (-0.77, 0.11) | 0.139 |
| **PC Hannum residuals** | - | -0.2 (4.63) | 0.17 (4.63) | -0.37 (-0.78, 0.04) | **0.077** | -0.41 (-0.79, -0.03) | **0.034** | -0.19 (-0.60, 0.21) | 0.347 | -0.20 (-0.63, 0.22) | 0.353 |
| **PhenoAge residuals** | - | 0.44 (6.08) | -0.39 (6.08) | 0.83 (0.28, 1.38) | **0.003** | 0.52 (0.00, 1.04) | **0.05** | 0.54 (-0.01, 1.09) | 0.056 | 0.40 (-0.18, 0.97) | 0.173 |
| **PC PhenoAge residuals** | - | 0.38 (5.65) | -0.34 (5.65) | 0.72 (0.22, 1.23) | **0.005** | 0.59 (0.20, 0.98) | **0.003** | 0.56 (0.15, 0.98) | **0.007** | 0.46 (0.03, 0.89) | **0.035** |
| **GrimAge residuals** | - | 0.14 (2.87) | -0.13 (2.87) | 0.27 (0.01, 0.53) | **0.041** | 0.33 (0.10, 0.55) | **0.005** | 0.37 (0.13, 0.62) | **0.003** | 0.30 (0.04, 0.55) | **0.022** |
| **JHS** | | | | | | | | | | | |
| **Horvath2** | 0.905 | 50.96 (9.37) | 50.31 (9.37) | 0.65 (-0.90, 2.20) | 0.413 | -0.17 (-0.84, 0.50) | 0.613 | -0.19 (-0.86, 0.48) | 0.577 | 0.09 (-0.63, 0.81) | 0.810 |
| **PC Horvath2** | 0.813 | 50.7 (7.85) | 50.48 (7.85) | 0.21 (-1.08, 1.51) | 0.747 | -0.00 (-0.69, 0.69) | 0.994 | -0.03 (-0.73, 0.66) | 0.924 | 0.23 (-0.52, 0.97) | 0.553 |
| **Hannum** | 0.87 | 47.42 (9.07) | 46.97 (9.07) | 0.45 (-1.05, 1.95) | 0.558 | -0.32 (-1.03, 0.39) | 0.382 | -0.36 (-1.07, 0.35) | 0.322 | -0.10 (-0.88, 0.68) | 0.795 |
| **PC Hannum** | 0.852 | 57.17 (8.53) | 56.45 (8.53) | 0.72 (-0.69, 2.13) | 0.315 | -0.10 (-0.79, 0.59) | 0.776 | -0.13 (-0.83, 0.56) | 0.705 | 0.09 (-0.66, 0.85) | 0.807 |
| **PhenoAge** | 0.824 | 44.97 (10.25) | 43.92 (10.25) | 1.05 (-0.64, 2.74) | 0.223 | -0.10 (-1.05, 0.84) | 0.830 | -0.20 (-1.15, 0.75) | 0.677 | -0.13 (-1.16, 0.90) | 0.805 |
| **PC PhenoAge** | 0.831 | 60.05 (10.38) | 58.15 (10.38) | 1.90 (0.18, 3.62) | **0.030** | 0.35 (-0.48, 1.17) | 0.412 | 0.26 (-0.56, 1.09) | 0.534 | 0.49 (-0.40, 1.38) | 0.282 |
| **GrimAge PC** | 0.939 | 69.15 (9.71) | 67.72 (9.71) | 1.43 (-0.17, 3.03) | 0.079 | 0.07 (-0.40, 0.54) | 0.775 | 0.02 (-0.45, 0.49) | 0.940 | 0.01 (-0.49, 0.52) | 0.954 |
| **DunedinPACE** | 0.128 | 1.06 (0.12) | 1.03 (0.12) | 0.02 (0.00, 0.04) | **0.018** | 0.01 (-0.01, 0.03) | 0.404 | 0.01 (-0.01, 0.02) | 0.592 | 0.00 (-0.02, 0.02) | 0.725 |
| **Horvath2 residuals** | - | -0.1 (4) | 0.01 (4) | -0.11 (-0.77, 0.55) | 0.746 | -0.17 (-0.84, 0.49) | 0.608 | -0.20 (-0.87, 0.47) | 0.566 | 0.09 (-0.63, 0.81) | 0.800 |
| **PC Horvath2 residuals** | - | -0.32 (4.61) | 0.03 (4.61) | -0.36 (-1.11, 0.40) | 0.357 | -0.01 (-0.70, 0.69) | 0.986 | -0.04 (-0.73, 0.65) | 0.906 | 0.24 (-0.51, 0.98) | 0.537 |
| **Hannum residuals** | - | -0.23 (4.49) | 0.02 (4.49) | -0.26 (-1.00, 0.48) | 0.498 | -0.32 (-1.04, 0.39) | 0.375 | -0.37 (-1.09, 0.34) | 0.308 | -0.09 (-0.87, 0.70) | 0.826 |
| **PC Hannum residuals** | - | 0.07 (4.51) | -0.01 (4.51) | 0.08 (-0.66, 0.81) | 0.842 | -0.11 (-0.80, 0.59) | 0.766 | -0.14 (-0.84, 0.55) | 0.685 | 0.10 (-0.65, 0.86) | 0.787 |
| **PhenoAge residuals** | - | 0.27 (5.83) | -0.03 (5.83) | 0.30 (-0.66, 1.26) | 0.541 | -0.11 (-1.06, 0.84) | 0.822 | -0.21 (-1.16, 0.74) | 0.664 | -0.12 (-1.15, 0.91) | 0.819 |
| **PC PhenoAge residuals** | - | 1.03 (5.8) | -0.1 (5.8) | 1.13 (0.18, 2.08) | **0.020** | 0.34 (-0.50, 1.17) | 0.429 | 0.24 (-0.59, 1.08) | 0.565 | 0.51 (-0.39, 1.41) | 0.268 |
| **GrimAge residuals** | - | 0.57 (3.33) | -0.06 (3.33) | 0.62 (0.08, 1.17) | **0.026** | 0.07 (-0.40, 0.54) | 0.77 | 0.02 (-0.45, 0.49) | 0.926 | 0.02 (-0.49, 0.52) | 0.953 |
| **Meta analysis** | | | | | | | | | | | |
| **Horvath2** |  | 56.60 (5.00) | 56.12 (5.00) | 0.44 (-0.06, 0.94) | 0.086 | 0.02 (-0.27, 0.30) | 0.914 | 0.03 (-0.26, 0.31) | 0.857 | 0.11 (-0.19, 0.40) | 0.483 |
| **PC Horvath2** |  | 54.61 (4.89) | 54.87 (4.89) | -0.40 (-1.01, 0.20) | 0.192 | -0.46 (-0.95, 0.03) | **0.065** | -0.27 (-0.64, 0.10) | 0.158 | -0.17 (-0.80, 0.46) | 0.595 |
| **Hannum** |  | 45.47 (5.33) | 45.29 (5.33) | 0.10 (-0.46, 0.65) | 0.732 | -0.50 (-0.88, -0.13) | **0.008** | -0.35 (-0.71, 0.01) | 0.054 | -0.29 (-0.67, 0.09) | 0.134 |
| **PC Hannum** |  | 59.67 (5.19) | 59.46 (5.19) | 0.05 (-0.56, 0.67) | 0.871 | -0.38 (-0.74, -0.02) | **0.037** | -0.17 (-0.52, 0.18) | 0.339 | -0.14 (-0.51, 0.23) | 0.462 |
| **PhenoAge** |  | 48.85 (6.25) | 47.73 (6.25) | 1.14 (0.47, 1.80) | **0.001** | 0.45 (-0.04, 0.93) | 0.072 | 0.34 (-0.25, 0.92) | 0.256 | 0.25 (-0.25, 0.75) | 0.329 |
| **PC PhenoAge** |  | 58.83 (6.22) | 57.47 (6.22) | 1.17 (0.53, 1.82) | **<0.001** | 0.56 (0.19, 0.93) | **0.003** | 0.52 (0.16, 0.89) | **0.005** | 0.44 (0.06, 0.82) | **0.024** |
| **GrimAge PC** |  | 71.36 (5.04) | 70.54 (5.04) | 0.68 (0.17, 1.19) | **0.010** | 0.32 (0.09, 0.55) | **0.006** | 0.29 (0.02, 0.56) | **0.033** | 0.23 (0.01, 0.46) | **0.043** |
| **DunedinPACE** |  | 1.03 (0.08) | 1.01 (0.08) | 0.02 (0.01, 0.03) | **<0.001** | 0.02 (0.01, 0.03) | **<0.001** | 0.02 (0.01, 0.02) | **0.001** | 0.01 (0.00, 0.02) | **0.027** |
| **Horvath2 residuals** |  | -0.00 (2.58) | -0.03 (2.58) | 0.08 (-0.19, 0.35) | 0.56 | -0.01 (-0.27, 0.26) | 0.967 | 0.03 (-0.26, 0.31) | 0.861 | 0.12 (-0.18, 0.41) | 0.448 |
| **PC Horvath2 residuals** |  | -0.37 (3.35) | 0.19 (3.35) | -0.69 (-1.06, -0.32) | **<0.001** | -0.36 (-0.90, 0.18) | 0.189 | -0.27 (-0.64, 0.10) | 0.156 | -0.16 (-0.80, 0.47) | 0.614 |
| **Hannum residuals** |  | -0.18 (3.23) | 0.07 (3.23) | -0.25 (-0.61, 0.11) | 0.179 | -0.47 (-0.82, -0.13) | **0.007** | -0.36 (-0.72, 0.01) | 0.054 | -0.27 (-0.66, 0.11) | 0.162 |
| **PC Hannum residuals** |  | -0.06 (3.23) | 0.08 (3.23) | -0.26 (-0.64, 0.12) | 0.184 | -0.34 (-0.67, -0.01) | **0.044** | -0.17 (-0.52, 0.18) | 0.336 | -0.13 (-0.50, 0.24) | 0.499 |
| **PhenoAge residuals** |  | 0.35 (4.21) | -0.20 (4.21) | 0.70 (0.22, 1.18) | **0.004** | 0.34 (-0.22, 0.89) | 0.240 | 0.33 (-0.25, 0.92) | 0.266 | 0.28 (-0.22, 0.78) | 0.280 |
| **PC PhenoAge residuals** |  | 0.70 (4.04) | -0.22 (4.04) | 0.81 (0.37, 1.26) | **<0.001** | 0.55 (0.19, 0.90) | **0.002** | 0.52 (0.15, 0.89) | **0.006** | 0.47 (0.08, 0.85) | **0.017** |
| **GrimAge residuals** |  | 0.32 (2.17) | -0.10 (2.17) | 0.36 (0.06, 0.66) | **0.019** | 0.28 (0.07, 0.48) | **0.008** | 0.29 (0.03, 0.55) | **0.030** | 0.24 (0.01, 0.47) | **0.038** |
